# Synthesis and Biological Evaluation of Some New 3-Aryl-2-thioxo-2,3-dihydroquinazolin-4(1*H*)-ones and 3-Aryl-2-(benzylthio)quinazolin-4(3*H*)-ones as Antioxidants; COX-2, LDHA, α-Glucosidase and α-Amylase Inhibitors; and Anti-Colon Carcinoma and Apoptosis-Inducing Agents

**DOI:** 10.3390/ph16101392

**Published:** 2023-10-01

**Authors:** Nahed Nasser Eid El-Sayed, Taghreed M. Al-Otaibi, Assem Barakat, Zainab M. Almarhoon, Mohd. Zaheen Hassan, Maha I. Al-Zaben, Najeh Krayem, Vijay H. Masand, Abir Ben Bacha

**Affiliations:** 1 Egyptian Drug Authority (EDA), 51 Wezaret El-Zeraa St., Giza 35521, Egypt; 2 Department of Chemistry, College of Sciences, King Saud University, P.O. Box 2455, Riyadh 11451, Saudi Arabia; chemst222@gmail.com (T.M.A.-O.); ambarakat@ksu.edu.sa (A.B.); mzaben@ksu.edu.sa (M.I.A.-Z.); 3 Department of Pharmaceutical Chemistry, College of Pharmacy, King Khalid University, Abha 62529, Saudi Arabia; zaheen@kku.edu.sa; 4 Laboratoire de Biochimie et de Génie Enzymatique des Lipases, ENIS, Université de Sfax, Route de Soukra 3038, Sfax BP 1173, Tunisia; krayemnajeh@yahoo.fr; 5 Department of Chemistry, Vidya Bharati College, Camp, Amravati, Maharashtra 444602, India; vijamasand@gmail.com; 6 Biochemistry Department, College of Sciences, King Saud University, P.O. Box 22452, Riyadh 11495, Saudi Arabia; aalghanouchi@ksu.edu.sa

**Keywords:** colorectal cancer, oxidative stress, inflammation, COX-2, Warburg effect, LDHA, post prandial hyperglycemia, apoptosis, quinazolinone, ADMET

## Abstract

Oxidative stress, COX-2, LDHA and hyperglycemia are interlinked contributing pathways in the etiology, progression and metastasis of colon cancer. Additionally, dysregulated apoptosis in cells with genetic alternations leads to their progression in malignant transformation. Therefore, quinazolinones **3a**–**3h** and **5a**–**5h** were synthesized and evaluated as antioxidants, enzymes inhibitors and cytotoxic agents against LoVo and HCT-116 cells. Moreover, the most active cytotoxic derivatives were evaluated as apoptosis inducers. The results indicated that **3a**, **3g** and **5a** were efficiently scavenged DPPH radicals with lowered IC_50_ values (mM) ranging from 0.165 ± 0.0057 to 0.191 ± 0.0099, as compared to 0.245 ± 0.0257 by BHT. Derivatives **3h**, **5a** and **5h** were recognized as more potent dual inhibitors than quercetin against α-amylase and α-glucosidase, in addition to **3a**, **3c**, **3f** and **5b**–**5f** against α-amylase. Although none of the compounds demonstrated a higher efficiency than the reference inhibitors against COX-2 and LDHA, **3a** and **3g** were identified as the most active derivatives. Molecular docking studies were used to elucidate the binding affinities and binding interactions between the inhibitors and their target proteins. Compounds **3a** and **3f** showed cytotoxic activities, with IC_50_ values (µM) of 294.32 ± 8.41 and 383.5 ± 8.99 (LoVo), as well as 298.05 ± 13.26 and 323.59 ± 3.00 (HCT-116). The cytotoxicity mechanism of **3a** and **3f** could be attributed to the modulation of apoptosis regulators (Bax and Bcl-2), the activation of intrinsic and extrinsic apoptosis pathways via the upregulation of initiator caspases-8 and -9 as well as executioner caspase-3, and the arrest of LoVo and HCT-116 cell cycles in the G2/M and G1 phases, respectively. Lastly, the physicochemical, medicinal chemistry and ADMET properties of all compounds were predicted.

## 1. Introduction

Colorectal cancer (CRC) was ranked third and second in terms of incidence and mortality, respectively, accounting for 1.9 million of all new cancer cases and 900,000 deaths in 2020 worldwide. Notably, the incidence of CRC has shown a rising trend among individuals aged less than 50 years [1].

CRC neo-tumorigenesis is a complex and multistep process, linked to factors of oncogenesis such as oxidative stress (OS), chronic inflammation, reprogrammed glucose metabolism and diabetes mellitus (DM). In addition, defective signaling or changes in the expression of proteins regulating programmed cell death ‘’apoptosis’’ play crucial roles in the evolution of normal mucosa cells towards neoplastic transformations and the acquisition of new characteristics, among which evading cell death [2].

OS is a pathological state featuring the over-generation of cellular reactive oxygen and/or nitrogen species (RO/N S) as a result of a dysregulated intracellular metabolism, tissue inflammation or exposure to exogenous stimuli at an extent that exceeds the cellular antioxidant defenses [3]. In fact, OS is considered as a hallmark of carcinogenesis [3].

Regarding CRC, OS on intestinal mucosal cells is proven to damage phospholipid biological membranes via lipid peroxidation by free radicals [4]. Therefore, it causes rupture of the phospholipid bilayer of the cytoplasmic cellular membrane, with the loss of cellular homeostasis. It promotes the destruction of the two lipid bilayers of the endoplasmic reticulum (ER), the dynamic reservoir of Ca^2+^, leading to disruption of ER-Ca^2+^ homeostasis, which is implicated in the initiation and propagation of cancer [5]. Also, it causes oxidative damage to the mitochondrial membranes, which disrupts the cell energy metabolism [6].

Another aspect of involvement of OS in CRC pathogenesis is through production of malondialdehyde (MDA, one of the end products of lipid peroxidation), which promotes oxidative DNA damage via formation of adducts with DNA bases; deoxyguanosine and deoxyadenosine. s, thus initiating mutations in tumor suppressor genes and proto-oncogenes [6,7].

Furthermore, free radicals may modify proteins, thus diminishing or enhancing their catalytic activity. Also, free radicals may stimulate cell and tissue lesions by oxidizing carbohydrates [8], and they may exert pro-inflammatory and pro-proliferative roles via oxidizing cholesterol to oxysterols [9]. Interestingly, cancerous cells can maintain certain levels of ROS to promote their proliferation and invasion [4]. Moreover, ROS are key constituents of cancer cells’ survival via the generation of resistance to chemo- and radiotherapies [10]. Accordingly, several studies have indicated that antioxidant compounds were supplemented or tested as adjuvants in cancer therapy to reverse resistance mechanisms, to reduce systemic toxicity during chemo- or radiotherapy [11] and to prevent cancer cell invasion [12].

Chronic inflammation is another principal effector in colorectal carcinogenesis. Inflammation can be induced by different stimuli, including pathogens, toxic compounds and OS-damaged cells. This inflammation enhances the affected tissues to overexpress the inducible cyclooxygenase isoform-2 (COX-2), which is typically absent or rarely expressed in healthy tissues. COX-2 catalyzes the conversion of arachidonic acid (AA) to prostaglandins (PGs) H_2_, which are further metabolized by prostaglandin synthases to PGD_2_, E_2_, I_2_, F_2α_ and thromboxane A_2_. Extensive clinical and experimental studies conducted over the last few decades have provided convincing data indicating that high levels of COX-2 and PGE_2_ are implicated in the pathogenesis of colorectal carcinoma [13,14,15], and that they are associated with poorer survival outcomes [16]. The key roles of the COX-2/PGE2 pathway in CRC pathogenesis include generating an immunosuppressive tumor microenvironment [17], participating in tumor initiation, promoting sustained angiogenesis and thus favoring tumor growth, inducing tumor cell invasion, encouraging migration, reducing apoptotic rate and enhancing chemotherapeutic resistance [18,19]. Therefore, inhibition of COX-2 to reduce PGE2 synthesis is considered an important therapeutic approach in prevention and modulation of CRC progression [20]. Consistent with these findings, a number of meta-analyses and in vitro studies have reported that classical non-steroidal anti-inflammatory drugs (NSAIDs) [21] and selective COX-2 inhibitors [22] provided protection against CRC incidence, reduced mortality and improved chemosensitivity and the efficacy of anticancer drugs when co-administrated [23].

Furthermore, the reprogrammed energy metabolism or Warburg effect [24] is a prominent metabolic characteristic of malignant cells [25]. The common features of this abnormal, less-energy-efficient metabolic pathway include increased glucose uptake, production of two moles of pyruvate and less ATP (2 moles/ glucose molecule) and rapid cytoplasmic fermentation of pyruvate to lactate under catalysis of lactate dehydrogenase A (LDHA). Lactate is then exported to the extracellular environment, which, in the presence of hydrogen ion, generate an acidic tumor microenvironment (TME). Lactic acidosis has been observed in different cancer types and it is linked to tumor aggressiveness [26]. In this regard, several clinical studies have revealed that hyperlactatemia is involved not only in tumor growth but also in the evasion of immune surveillance [27], local invasion, metastasis, chemoresistance and patient survival [28]. Consequently, targeting glycolysis via inhibiting LDHA would suppress hyperlactatemia and prevent its protumorigenic effects, in addition to depriving cancerous cells of nutrients, thereby killing them [29]. Indeed, several studies have shown that inhibition of LDHA activity impedes tumor progression, induces significant oxidative stress and necrosis [30], induces G2/M cell cycle arrest, increases sensitivity to ionizing radiation and 5-Fluorouracil (5-FU) [31,32] and attenuates cell invasion and migration [33].

DM is a multifactorial chronic metabolic disorder affecting millions of people worldwide. DM is categorized into Type 1 (T1DM, caused by the autoimmune destruction of the pancreatic β-cells leading to little, or the absolute lack of, insulin production), Type 2 (T2DM, arises due to complex interactions between genes and some risk factors, mainly obesity, smoking and hypertension, leading to insulin resistance) and gestational (GDM, occurs in pregnant women). T2DM is the most prevalent type, accounting for about 90% of all diabetic cases [34]. Besides its deadly cardiovascular complications, T2DM has also been associated with an increased risk for the development of certain types of cancers [35]. In particular, the positive correlation between CRC and T2DM is attributed to sharing some common risk factors including obesity, alcohol consumption, cigarette smoking and a Western-pattern diet. In addition, genome-wide association studies have identified transcription factor 7-like 2 (TCF7L2), gremlin 1 (GREM1) and tumor protein p53 inducible nuclear protein 1 (TP53INP1), among other genes, as being pleiotropically related to T2DM as well as CRC prognosis and tumorigenesis [36]. Moreover, T2DM-related metabolic changes, such as hyperinsulinemia, hyperglycemia, OS, inflammation induced by adipose tissue dysfunction, impaired immunological surveillance and gastrointestinal motility disorder have been implicated in carcinogenesis [37].

With regard to hyperglycemia, it influences the neoplastic transformations by providing glucose that is essential for the growth and proliferation of cancerous cells under the Warburg state [38]. It leads to the irreversible formation of advanced glycated end-products (AGEs) with proteins, lipids and nucleic acids, as well as their buildup, resulting in the development of OS and chronic inflammations, which are intrinsically linked to induction and development of cancer. Furthermore, elevated expressions of AGEs and their receptors contribute to the invasion and metastasis of CRC [39]. Also, the activations of the polyol, hexosamine and protein kinase C metabolic pathways [40] are other putative mechanisms by which hyperglycemia contributes to the cell proliferation, invasion and tumor progression of colon cancer. In addition, hyperglycemia may negatively affect CRC patients’ quality of life by reducing the chemotherapeutic efficacy and inducing adverse effects [36].

Thus, over the years, researchers have worked on various targets to moderate the onset or worsening of diabetes, from which some antihyperglycemic therapies have been approved by the US FDA and other regulatory agencies, which exhibit different mechanisms of action via targeting single or multiple established molecular pathways involved in the pathogenesis of the disease [41]. Amongst these validated pathways is the breakdown of complex carbohydrates into absorbable monosaccharides, which takes place under the catalysis of alpha-glucosidase (AG) and alpha-amylase (AA). Thus, inhibitors (Is) of these enzymes would manage T2DM by delaying the carbohydrate metabolism and turning down the rate of glucose absorption to reduce the post postprandial blood glucose level (PPBGL). Indeed, carbohydrate mimics [34], including voglibose (natural sugar), miglitol (semisynthetic iminosugar) and 1-deoxynojirimycin (DNJ), all of which inhibit AG, in addition to acarbose (an oligosaccharide), which can also inhibit AA [42], are marketed as antidiabetic drugs. Despite the helpful impacts of these glucose-lowering drugs, morbidity and mortality remain significant in T2DM patients. In addition, these medications have many drawbacks, such as poor compliance with treatment, expense and adverse effects [43,44]. Therefore, there are a lot of synthetic efforts towards the development of new inhibitors, particularly annulated sugars [45], iminosugars [46,47] and novel classes of drugs derived from the structural hybrids of different key molecules and heterocycles [48], with the aim to develop new effective inhibitors that are escorted by better compliance, socioeconomic benefits and safety [49].

Additionally, apoptosis is a well-characterized form of programmed cell death (PCD), which occurs normally for regulating tissue homeostasis and regeneration during development. However, it is also activated as an immune defense mechanism against damaged or stressed cells in response to stimuli such as reactive oxygen species (produced by some chemotherapeutic agents) or DNA damage (from irradiation or anticancer drugs) to remove these potentially harmful cells [50]. If apoptosis in not controlled properly (insufficient apoptosis/extreme cell proliferation), mutations will potentially accumulate in the unwanted cells, which eventually could lead to the induction of cancer. Indeed, resisting apoptosis is considered as one of the hallmarks of cancer [51].

Apoptosis can be triggered by two different pathways: the extrinsic (death receptor) and the intrinsic (mitochondrial) pathways. The extrinsic pathway is initiated via the binding of certain extracellular ligands (members of the tumor necrosis factor super family, TNF) to their cognate cell surface death receptors, resulting in caspase 8 activation. This activation can directly induce apoptosis, or activate effector caspase 3 or Bid, which lead to apoptosis. While the intrinsic apoptotic pathway is initiated via mitochondrial outer membrane permeabilization (MOMP), a process controlled by the members of the B-cell lymphoma (BCL-2) family and p53 proteins [52]. MOMP is considered as the point of no return in this apoptotic cascade, since it leads to the release of the apoptogenic proteins cytochrome c and Smac from the mitochondria into the cell cytoplasm, which activate caspases directly or indirectly, respectively [53,54]. According to the direct mode, cytochrome c interacts with apoptotic protease activating factor-1 (APAF1), thus transforming it into an apoptosome complex, which activates pro-caspase-9 to initiator caspase 9, which in turn activates caspase-3, leading to the activation of the final cascade and, consequently, the fragmentation of the nucleus after its membrane rupture [55]. After the activation of all signaling cascade mediators, the two pathways meet up at the final caspases’ activation (caspases 3, 7), resulting in the cleavage of different integrated cell proteins and the execution of the apoptotic steps [55].

Furthermore, the BCL-2 family of proteins [56], which tightly regulate the intrinsic pathway, is subclassified into three subgroups; the first comprises the pro-apoptotic proteins or apoptosis effectors that positively regulate apoptosis, and they include Bcl-2 antagonist killer 1 (Bak) and Bcl-2-associated X protein (Bax), which can directly execute MOMP when activated.

The second comprises the pro-survival members that negatively regulate apoptosis, and they include B cell lymphoma-2 (Bcl-2) itself, B cell lymphoma extra-large (Bcl-xL), B cell lymphoma-w (Bcl-W), B cell lymphoma-like protein 2 (Bcl-B), myeloid leukemia cell 1 (MCL-1) and Bcl-2-related protein A1/Bcl-2-related isolated from fetal liver-11 (A1/BFL-1).

The third group is the pro-apoptotic BH3-only proteins, which include p53-upregulated modulator of apoptosis (Puma), Bcl-2-interacting mediator of cell death (Bim) and the Bcl-2 Homology 3 interacting death agonist (Bid), which act as activators to Bax and Bak. This group comprises also other proteins, called sensitizers, which do not associate with Bax and Bak, but antagonize the pro-survival members, including the Bcl-2 associated agonist of cell death (Bad), Bcl-2 interacting t killer 1 (Bik), Noxa, BMF and HRK.

In healthy cells, the pro- and anti-apoptotic proteins are held in a fine, delicate balance. Conversely, during cancer development, the malignant cells alter this balance, by overexpressing various anti-apoptotic proteins and/or abnormally reduce the expression levels of pro-apoptotic members, and thus they become unresponsive to stimuli that otherwise trigger apoptosis in sensitive cells, which leads to cellular proliferation and cancer progression, in addition to therapeutic resistance [57]. Therefore, inhibiting the anti-apoptotic [58,59] and activating pro-apoptotic members [60] of the Bcl-2 family have been suggested as plausible selective strategies for cancer therapy and to overcome drug resistance.

**Figure 1:**
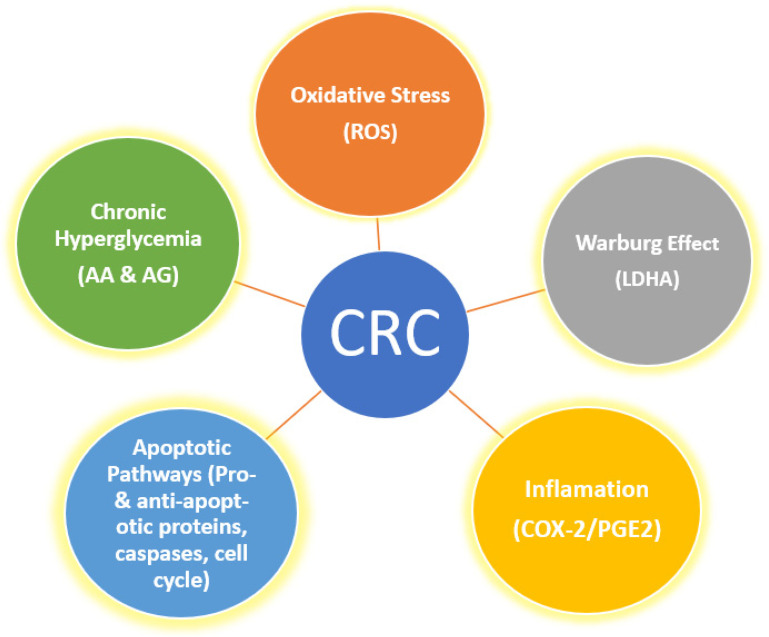
Various risk factors and pathways implicated in cancer, which serve as targets for multi-potent agents.

Taken together, the complexity of CRC and the manifold and heterogeneous association of this malignancy with OS, inflammation, Warburg effect and hyperglycemia highlight the need for the development of new pharmaceuticals capable of the simultaneous targeting of these risk factors as well as the apoptotic pathways (Figure 1). 

**Figure 2:**
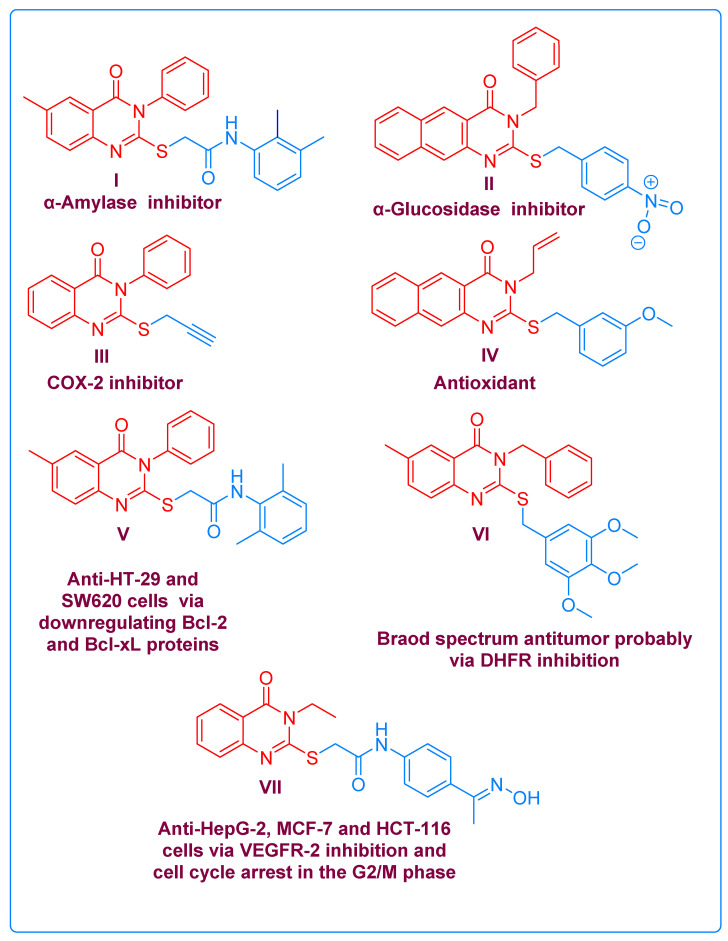
Selected examples of biologically active compounds containing quinazolinone cores.

The innovation of multi-targeted therapies represents a promising and prevailing anticancer drug discovery paradigm among medicinal chemists [61]. In line with this principle, quinazolinone scaffolds have proven to be easily accessible pharmacophores, possessing multipotent pharmacological activities both in vitro and in vivo [62]. They have been presented as core structures in inhibitors for; AA [63] **I**, AG [64] **II**, and COX-2 [65] **III**, as well as antioxidant agent [66] **IV**, and anti-cancer candidates (**V**, **VI** and **VII**), which produce their cytotoxic effects through different molecular mechanisms [63,67,68], as portrayed in Figure 2.

In light of the above, in this study, we designed a number of new quinazolinone derivatives bearing 4-nitro-phenyl, in addition to fluorine containing 4- or 3-(trifluoromethyl)phenyl moiety at position 3, due to the significant impact of fluorine atoms in enhancing the binding affinity to the targeted protein, as well as improving pharmacokinetic and physicochemical properties including metabolic stability and membrane permeation [69]. Also, various substituents such as Br, Cl and OCH_3_ were located at the 6 and/or 6,7 positions on the quinazolinone core, to span different electronic, steric and lipophilic characters that would produce different pharmacological responses, which would increase the probability of the discovery of active agents. Subsequently, these compounds were evaluated in vitro as antioxidants as well as AA, AG, COX-2 and LDHA inhibitors. Structure–activity relationships regarding the antioxidant activity were discussed. Additionally, molecular docking simulations were performed to confirm the results of the in vitro enzymatic inhibitory assays and to predict the conformations of the potential inhibitors upon binding to the target proteins, as well as to calculate the binding affinities between them.

Moreover, all compounds were screened for their cytotoxic effects on LoVo and HCT-116 human colon carcinoma cells at 200 µg/mL, and then the IC_50_ values of the most active cytotoxic candidates were calculated form the concentration–response curves and compared with that of 5-FU. Afterwards, they were evaluated against non-tumoral human umbilical vein endothelial cells (HUVEC) to explore their safety profiles. Furthermore, the capability of the cytotoxic candidates to modulate apoptosis regulators (Bax and BcL2), to activate caspases-8, 9 and 3 and to disturb the cell cycles of LoVo and HCT-116 cells were investigated. Finally, the physicochemical, medicinal chemistry and ADMT properties of all compounds were predicted and discussed in detail for some active compounds as model examples.

## 2. Results and Discussion

### 2.1. Chemistry

**Scheme 1:**
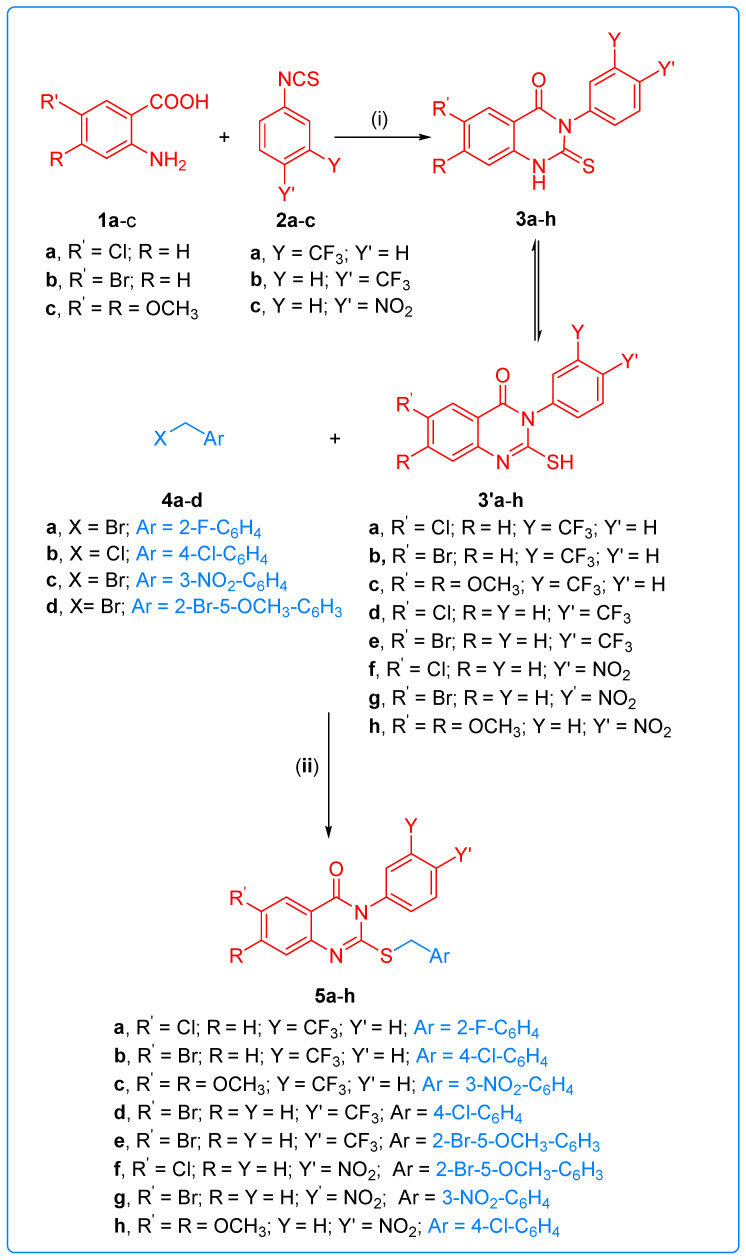
Reagents and conditions: (i) Glacial AcOH, reflux 10 h. (ii) Dry acetone, K_2_CO_3_, reflux 6 h.

The target compounds were synthesized as depicted in Scheme 1.

Initially, refluxing mixtures of 5-substituted or 4,5-disubstituted anthranilic acid derivatives **1a**-**c** and 3-substituted or /4-substituted phenyl isothiocyanate derivatives **2a**-**c** in glacial acetic for 10 h [70] yielded the new 6-substituted or 6,7-disubstituted-3-aryl-2-thioxo-2,3-dihydro-1*H*-quinazolin-4-one derivatives **3a**-**h**, which can potentially also exist in their tautomeric forms **3′a**-**h** due to thiocarbonyl–thiol tautomerization. Subsequently, the arylation of the acidic thiol groups using various aryl halides **4a**–**d** under basic catalysis with potassium carbonate in refluxing dry acetone for 6 h [50] produced eight new 2-(benzylsulfanyl)-3-aryl-3*H*-quinazolin-4-one derivatives, **5a**–**h**. The structures of the synthesized derivatives were elucidated based on IR, ^1^H-NMR, ^13^C-NMR and mass spectrometric analyses.

All the spectroscopic data indicated the predominance of the thiocarbonyl tautomeric form for compounds **3a**–**h** in the solid state (IR) and in the DMSO solution (NMR), which is in agreement with the reported data [71]. Thus, their IR spectra showed stretching absorption bands for the NH groups at a ν_max_ ranging from 3155 to 3245 cm^−1^. In addition, the ^1^HNMR spectra demonstrated the NH protons as singlet peaks at δ_H_ = 12.91–13.24 ppm, while the ^13^CNMR spectra exhibited characteristic signals for C= S at a δ_C_/ppm ranging from 174.38 to 176.28.

Contrarily, the IR and ^1^HNMR spectra of derivatives **5a**–**h** indicted the disappearance of the NH protons, and their ^13^CNMR spectra confirmed the absence of the peaks corresponding to the C=S groups and the presence of the benzylic methylene groups.

As representative examples, the IR spectrum of compound **3c** showed the stretching absorption bands at ν_max_ = 3178 and 1698 cm^−1^, corresponding to the NH and C=O groups, respectively. The ^1^H-NMR spectrum of this compound (500 MHz; DMSO-d_6_) showed a characteristic one-proton singlet peak at δ_H_ = 12.91 ppm for the NH proton. Additionally, the four aromatic protons of the 3-trifluoromethylphenyl ring exhibited a one-proton doublet peak (coupling constant *J* value = 7.5 Hz), a one-proton singlet peak, an apparent triplet peak (*J* value = 8.05 Hz) and a doublet peak ((*J* value = 8.5 Hz) at chemical shift values of δ_H_ = 7.73, 7.69, 7.66, 7.24 and 7.56 ppm, respectively. The two aromatic protons of the dimethoxyphenyl ring were detected as two singlet peaks at δ = 7.24 and 6.95 ppm.

Lastly, the two methoxy groups exhibited two singlet peaks, each integrating to three protons at δ_H_ = 3.83 and 3.77 ppm. With regard to its ^13^C-NMR spectrum (125 MHz; DMSO-d_6_), it was characterized by a distinctive peak at δ_C_/ppm = 174.66 attributed to C=S group, in addition to seventeen peaks, as expected, at δ_C_/ppm = 159.34 (C=O), 155.41, 146.58, 140.23, 135.63, 133.62, 130.03, 129.80, 126.38, 125.03, 124.89, 122.87, 108.66, 106.94, 97.98 (6 × CH-aromatic, 6 × C_q_-aromatic and C_q_F_3_), 56.01 and 55.84 (2 × OCH_3_). Finally, the MS spectrum of this compound showed a molecular ion peak [M^+^ + 1] at *m*/*z* = 383.17 (27.26%) for C_17_H_13_F_3_N_2_O_3_S, and the base peak was detected at *m*/*z* = 382.16, which corresponds to [M^+^].

On the other hand, the IR spectrum (KBr) of compound **5c** indicated the disappearance of the stretching absorption band due to NH group of its precursor **3c**. The ^1^H-NMR spectrum (300 MHz, DMSO-d_6_) endorsed the absence of NH proton and the presence of the characteristic benzylic protons as an AB quartet signal at δ_H_ = 4.53 ppm, with a coupling constant of *J* = 13.5 Hz. In addition, it displayed eight peaks corresponding to ten aromatic protons as follows: a one-proton apparent doublet of the doublet peak (*J* values = 6.3, 2.1 Hz); an apparent one-proton doublet of the doublet peak (*J* values = 8.4, 2.4 Hz); a one-proton singlet; a two-proton doublet peak (*J* = 7.8 Hz); a two-proton multiplet peak; a one-proton triplet peak (*J* = 7.8 Hz); a one-proton singlet peak and a one-proton singlet peak, which were resonating at δ_H_ = 8.52, 8.09, 7.98, 7.93, 7.83–7.77, 7.60, 7.37 and 7.24 ppm, respectively, confirming the installation of the 3-nitrophenyl moiety on the sulfur atoms. The ^13^C-NMR spectrum (150 MHz; DMSO-d_6_) revealed the absence of the peak due to the C=S group and the presence a new characteristic CH_2_-benzylic peak at δ_C_ = 35.12 ppm. The mass spectrum of this compound indicated the presence of the molecular ion peaks [M^+^ + 2] and [M^+^ + 1] at *m*/*z* (%) = 519.10 (6.53) and 518.19 (29.95), respectively, for C_24_H_18_F_3_N_3_O_5_S. The base peak, which represents [M^+^] ion was recorded at *m*/*z* = 517.18.

### 2.2. Biological Evaluation

The synthesized compounds were subjected to multiple in vitro biological evaluations as described in the following sections. 

#### 2.2.1. Antioxidant Evaluation via 2,2-Diphenyl-1-picryl-hydrazyl-hydrate (DPPH) Assay

The DPPH free radical scavenging method is used for the determination of the antioxidant potency of compounds **3a**-**h** and **5a**-**h**, expressed as mean IC_50_ values; half maximal inhibitory concentrations. The results are summarized in Table 1.

In principle, the antioxidant power of a compound in this assay is correlated to its tendency to reduce the stable, purple-colored DPPH radical through a hydrogen atom transfer (HAT) mechanism and/or a single electron transfer mechanism [72], thereby converting it to the yellow-colored 2,2-diphenyl-1-picrylhydrazine, whereby this reduction is accompanied by a depletion in the amount of the initial DPPH radicals and a change in the absorbance of the test solution accordingly, which can be detected relative to the blank solution at λ = 517 nm.

The antioxidant potency of a compound depends on its structural characteristics due to the electronegativity, position and number of substituents. Therefore, among the tested compounds (Table 1), the quinazolinone derivatives **3a**, **3g** and **5a** demonstrated the lowest mean half maximal concentrations for scavenging DPPH radicals with IC_50_ values (mM) of 0.191 ± 0.011, 0.165 ± 0.005 and 0.172 ± 0.004, respectively, which were lower than 0.245 ± 0.027, the value of the reference antioxidant drug, butylated hydroxy toluene (BHT), indicating that these quinazolinones are more potent antioxidants than BHT. The rest of the compounds exhibited IC_50_ (mM) values ranging from 0.262 ± 0.037 to 1.520 ± 0.039.

For quinazolinones **3a**–**3h** and **5a**–**5h**, the IC_50_ (mM) values spanned from 0.191 ± 0.011 to 1.520 ± 0.039, and from 0.172 ± 0.004 to 0.752 ± 0.041, respectively. Generally, it was observed that all *S*-arylated derivatives were more efficient than their thioxo precursors, except for derivatives **5c** and **5g**, which were less active than their counter substrates **3a** and **3g**, respectively; this may be attributed to the arylation of the S-atom by the 3-nitrobenzyl group.

Interestingly, among the derivatives of the **3a**–**h** series, the highest and the poorest scavenging efficiencies were associated with derivatives **3g** and **3b**, which share the 6-bromo-2-thioxo-2,3-dihydroquinazolin-4(1*H*)-one core, but differ in the type and location of the substituents on the 3-phenyl groups, where the former possessed 3-(4-nitrophenyl), whereas the latter possessed 3-(3-(trifluoromethyl)phenyl moiety. Along this line, comparing the efficiencies of compounds **3a**, **3c** and **3b** having the 3-(3-(trifluoromethyl)phenyl group and different substituents on the 2-thioxo-2,3-dihydroquinazolin-4(1*H*)-one core including 6-Cl, 6,7-di-MeO and 6-Br, respectively, revealed the superiority of compounds **3a** (8.0-fold) and **3c** (5.8-fold) to derivative **3b**. Furthermore, comparing the antiradical potencies of analogs **3b** and **3e** having exactly the same structure, but with the trifluoromethyl(CF_3_) substituent located at positions 3 and 4 on the 3-phenyl group, respectively, indicated that introducing CF_3_ at position 4 enhanced the activity by 2.1-fold.

Additionally, comparing derivatives **3a**, **3d** and **3f**, which possessed the 6-chloro-2-thioxo-2,3-dihydroquinazolin-4(1*H*)-one nucleus, revealed that the 3-(3-CF_3_)phenyl group and the 3-(4-CF_3_)phenyl group enhanced the antioxidant potency by 3.6- and 1.4-fold, respectively, as compared to the 3-(4-NO_2_)phenyl substituent.

All of these observations indicated that in series **3a**–**h**, the combination of the 3-(3-(trifluoromethyl)phenyl group with a 6-chloro-2-thioxo-2,3-dihydroquinazolin-4(1*H*)-one core improved the antiradical efficiency (as in case of **3a**). Contrarily, its combination with 6-bromo-2-thioxo-2,3-dihydroquinazolin-4(1*H*)-one core (as in case of **3b**) greatly reduced the activity, which was improved upon tethering the 4-chlorobenzyl moiety to the S-atom by 4.8-fold (as in case of **5b**). The weakest antioxidant in the **5a**-**h** series was **5h**, which possessed the 6,7-dimethoxy-3-(4-nitrophenyl)quinazolin-4(3*H*)-one core and lacked substituents at the *o* or *m*-positions on the 3-phenyl and the *S*-benzyl groups.

#### 2.2.2. In Vitro Enzyme Inhibition Assays

##### Cyclooxygenase-2 (COX-2) Assay

The inhibitory potential of compounds **3a**-**h** and **5a–h** towards the inducible pro-inflammatory COX-2 enzyme was examined at 0.100 and 0.200 mg/mL concentrations and the results are presented in Table 2.

Although the obtained data indicated that the inhibitory efficiency increased by duplicating the concentration of the tested compounds by 1.41- (**5c**) up to 2.58 (**5h**)-fold, only **3a** and **3g** exhibited potent inhibitory efficiencies of 97.050 ± 1.344 and 98.900 ± 1.556%, respectively, at 0.200 mg/mL (i.e., at 0.561 mM and 0.529 mM, respectively) as compared with the 100.00 ± 0.00% inhibitory effectiveness of celecoxib at 0.10 mg/mL (0.262 mM). Moreover, at 0.200 mg/mL concentration, compound **5a** produced an inhibitory efficiency equal to 63.150 ± 5.869% and the rest of the compounds exhibited poor inhibitory capabilities ranging from 10.750 ± 2.475 to 52.000 ± 2.828%.

##### Lactate Dehydrogenase A (LDHA) Assay

Furthermore, the studied compounds were assessed for their LDHA inhibitory potency (Table 3) at 0.100 and 0.200 mg/mL concentrations using sodium oxamate as the reference inhibitor. Again, the inhibitory efficiencies of the tested compounds at 0.200 mg/mL were increased by 0.20- (**3c**) up to 1.84 (**3a**)-fold than at the 0.100 mg/mL concentration. Also, each compound in the **3a**–**h** series was found to be more active than its counterpart in the **5a**–**h** series at both concentrations, except for derivative **3f** (34.250 ± 1.768%) and its *S*-alkylated product **5f** (51.050 ± 3.606%), which may indicate the importance of the NH group for LDHA inhibition.

Overall, derivatives **3g** and **3a** were recognized as the most active derivatives, with inhibitory efficiencies of 100.000 ± 0.000 and 98.450 ± 1.344 at 0.200 mg/mL (0.561 mM and 0.529 mM), respectively, as compared with the 100.000 ± 0.000% inhibitory efficiency of sodium oxamate at 0.111 mg/mL (1 mM). Moreover, derivatives **3d** and **5f** demonstrated inhibitory effectiveness of 53.500 ± 2.121% and 51.050 ± 3.606% at 0.200 mg/mL (0.561 and 0.375 mM, respectively), whereas the rest of the compounds exerted inhibitory efficiencies ranging from 14.300 ± 1.556 to 43.900 ± 1.556%.

Although analogs **3a** and **3d** as well as **3b** and **3e** have exactly the same structure, except for locating the CF_3_ group on the 3-phenyl moiety in each pair at positions 3 and 4, respectively, it was observed that the structures with 3-CF_3_ were more potent than those with 4-CF_3_, implying the importance of this substituent at the position 3 for LDHA inhibition.

In the view of the COX-2 and LDHA inhibitory assays, the IC_50_ values in µg/mL and (µM) for the most active compounds, **3a** and **3g**, were determined to be 281.374 ± 10.545 and 251.780 ± 22.023 against COX-2, respectively, as compared with 0.136 × 10^−3^ ± 0.006 × 10^−3^ for celecoxib. Likewise, the IC_50_ values against LDHA were calculated to be 273.345 ± 16.087 and 242.279 ± 31.298, respectively, compared to 140.503 ± 7.647 by sodium oxamate, as presented in Table 4.

##### Alpha-Glucosidase (AG) and Alpha-Amylase (AA) Assays

The studied compounds were evaluated in vitro for their AG and AA inhibitory activities via the determination of their IC_50_ values in µg/mL and µM, which were compared with quercetin as the reference drug (Table 5).

The results showed that the tested compounds exhibited IC_50_ values (µM) ranging from 12.548 ± 0.542 to 104.275 ± 1.012 against AG, and ranging from 186.437 ± 9.700 to 940.903 ± 8.978.563 against AA, compared with 13.126 ± 0.688 and 402.566 ± 10.108 for quercetin, respectively. Accordingly, derivatives **3h**, **5a** and **5h** were proven to be more potent AG inhibitors than quercetin. Congruently, eleven derivatives, **3a**, **3c**, **3f**, **3h** and **5a**-**5f**, as well as **5h**, exhibited superior inhibitory efficiencies (lowered IC_50_ in µM ± SD, ranging from 186.437 ± 9.700 by **5a** to 381.335 ± 8.713 by **5c**) to that of quercetin against AA. In addition, compound **3b** exhibited a modest potency against AA, with an IC_50_ value of 427.051 ± 10.376 µM.

Overall, compounds **3h**, **5a** and **5h** were recognized as dual inhibitors for AG and AA; thus, they would have the potential as hypoglycemic and anti-DM-type 2 agents.

It is noticeable that the dual inhibitors **3h** and **5h** possessed the same 6,7-dimethoxy-3-(4-nitrophenyl)quinazolin-4(3*H*)-one core, implying the importance of this moiety for the inhibition of both enzymes.

With regard to derivatives **3a** and **5a**, which possessed the 6-chloro-3-(3-(trifluoromethyl)phenyl)quinazolin-4(3*H*)-one core, only **5a** was found to be efficient against AG, which implies the importance of the 2-fluorobenzyl group for the inhibition of the AG enzyme.

Moreover, the dual inhibitor **5a** is also a potent radical scavenger; therefore, it may contribute to antidiabetic activities both directly, through inhibiting AA and AG, and indirectly, via its antioxidant properties.

#### 2.2.3. Cytotoxic Activity

The anticancer activities of all the synthesized compounds were assessed against two human colon carcinoma cell lines, namely LoVo and HCT-116 (Table 6), via the determination of the mean percents of the viable cancerous cells remaining after being incubated with 200 µg/mL of each test compound for 48 h using LDH assay [73]. 

The results shown in Table 6 indicated that **3a** and **3f** were the most active derivatives against both cell lines by reducing their viability percents to 23.500 ± 1.500 and 22.667 ± 2.082% (LoVo) and 26.833 ± 1.258 and 4.667 ± 0.577% (HCT-116), respectively. Three other compounds, namely **3g** (against HCT-116) and **3c** and **5b** (against LoVo) showed moderate antiproliferative effects with viability % of 42.333 ± 2.517, 47.333 ± 2.517 and 37.667 ± 2.517, respectively. The rest of the compounds demonstrated poor cytotoxic profiles, with viability percentages ranging from 55.667 ± 3.055 to 90.667 ± 3.512 (LoVo cells) and from 60.333 ± 2.517to 96.000 ± 1.732 (HCT-116 cells).

Accordingly, the cytotoxic effects of the compounds **3a**, **3c, 3f, 3g** and **5b**, which showed growth inhibitory efficiencies of less than 50% on one or both tumoral cells, were determined at the same test concentration against non-tumoral human umbilical vein endothelial cells (HUVEC) and were found to be 97.000 ± 2.646, 99.667 ± 0.577, 99.667 ± 0.577, 98.667 ± 1.155 and 96.333 ± 0.577, respectively, as compared to 57.333 ± 2.517% exerted by 5-flurouracil (**5-FU**), the reference anticancer drug (Table 6).

Based on the results of the viability assays, the IC_50_ values for the most active cytotoxic candidates, **3a**, **3c**, **3f**, **3g** and 5**b**, in µg/mL (µM) were calculated from the concentration–response curves and compared with that of **5-FU**, as shown in Table 7.

It is noteworthy to indicate that the viability of the normal HUVEC upon being treated with 200 µg/mL (1537.515 µM) of **5-FU** was reduced to 57.333 ± 2.517% and its IC_50_ was determined to be 298.500 ± 19.092 µg/mL (2294 ± 146.77 µM). On the other hand, the IC_50_ values (µg/mL) of **5-FU** were calculated to be 2.910 ± 0.028 (LoVo) and 11.850 ± 0.354 (HCT-116), implying its higher selectivity towards the examined cancerous cells, as indicated by the calculated selectivity indices (SIs, represent the ratio of IC_50 normal cell_ to IC_50 cell line_) [74], which were equal to 102.577 (LoVo) and 25.190 (HCT-116), respectively.

#### 2.2.4. Investigation of the Apoptosis-Inducing Properties as a Potential Molecular Basis for the Observed Cytotoxicity for Derivatives **3a** and **3f**

##### Effects of **3a** and **3f** on the Expressional Levels of Bcl-2 and Bax, Regulators of the Intrinsic Pathway

Collectively, the viability assays showed that derivatives **3a** and **3f** were the most active cytotoxic derivatives against both tested colon cell lines. However, the enzymatic inhibitory assays showed that only **3a** was active against COX-2 and LDHA; in addition, it is documented that HCT-116 cells are COX-2 negative [75]. Consequently, these observations imply that molecular targets other than COX-2 and LDHA could be responsible for the observed cytotoxicity on the tested cancerous cells.

Impaired apoptosis plays a main role in cancer cell survival, tumor progression and resistance to chemotherapy., In particular, the inhibition of the intrinsic apoptotic pathway depends partly on disturbing the balance between the expression of the Bcl-2 and Bax genes. Bcl-2 protein is a member of the anti-apoptotic (pro-survival) subgroup, and its expressional level in tumor cells is much higher than in normal cells; therefore, molecules targeting this protein would have little effect on normal cells [56]. On the other hand, Bax is a proapoptotic protein, whose overexpression induced by several agents enhances apoptosis, but its loss contributes to drug resistance in human cancers [76].

Bcl-2 inhibits apoptosis by forming a heterodimer with Bax or by inhibiting the activities of caspase-9, 3, 6, and 7, resulting in a prolonged survival time of tumor cells, thus causing their malignant transformation [56]. On the other hand, the activated Bax levels promote cell apoptosis by binding to the mitochondrial membranes, and increasing their permeability and cytochrome c releasing [77].

Considering these facts, enhancing apoptosis has been extensively studied as a potential drug target in cancer therapy [78], therefore, in this study, we investigated the effects of the most cytotoxic compounds, **3a** and **3f**, (at 10 µg/mL, 24 h incubation) on the expressional levels of these apoptotic regulator genes (Bax and Bcl-2) using quantitative reverse transcription–polymerase chain reactions (qRT-PCR).

**Figure 3:**
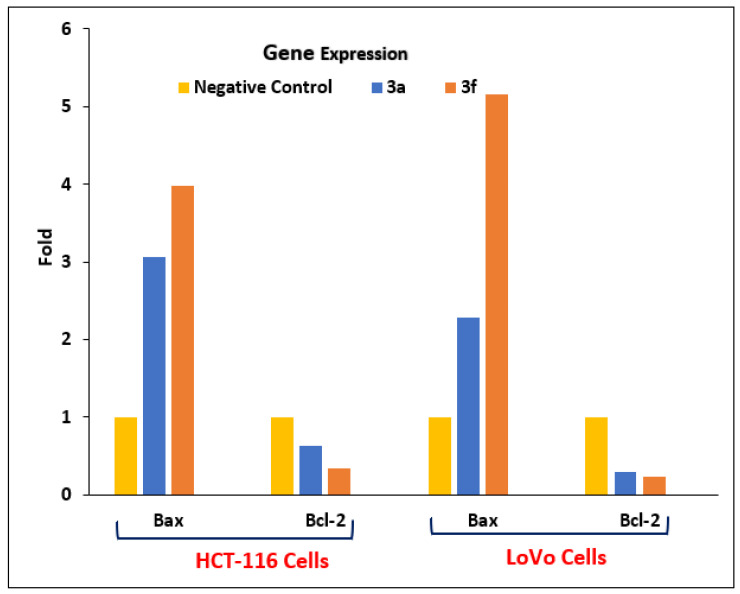
qRT-PCR analysis of the expressional levels of Bax and Bcl2 genes in cultures of LoVo and HCT-116 treated with 10 µg/mL of the cytotoxic candidates **3a** and **3f** for 24 h, as compared to negative control (untreated cells).

The results were compared with the negative control (untreated cells) cultures. The recorded results (Figure 3) in the expressional level of anti-apoptotic gene Bcl-2 (by 0.635- and 0.345-fold) in the **3a**- and **3f**-treated HCT-116 cell cultures, respectively, as compared to negative control culture.

##### Caspases Activation Induced by Derivatives **3a** and **3f**

The two distinctive apoptotic pathways converge on caspases activation. The extrinsic apoptosis pathway is mediated by the death receptor, while the intrinsic mitochondrial pathway is triggered via the interaction of cytochrome c with apoptotic protease activating factor-1 (APAF1) resulting activation of caspases [79]. In general, caspase -9 is activated by cytochrome c, released by the mitochondria in the intrinsic pathway, while the extrinsic pathway activates caspase-8. After the activation of these initiator caspases (-8 and -9), the effector step of apoptosis is then triggered by the activation of caspases -3, -6 and -7. The activities of these effectors induce observed morphological changes in the apoptotic cells, especially DNA fragmentation, after chromatin condensation and the apoptotic bodies’ formation [80].

**Figure 4:**
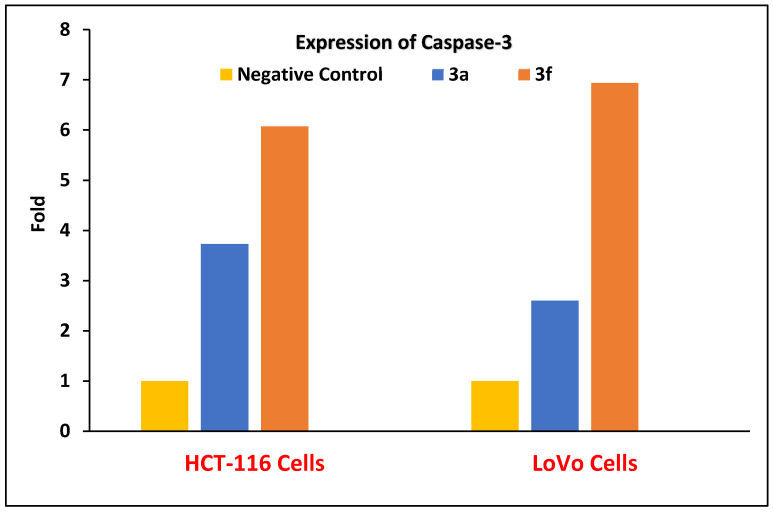
qRT-PCR analysis of the expressional levels of caspase-3 gene in cultures of LoVo and HCT-116 treated with 10 µg/mL of the cytotoxic candidates **3a** and **3f** for 24 h, as compared to negative control (untreated cells).

Therefore, in this study the expressional levels of caspase-3 as a major apoptosis effector were evaluated in **3a**- and **3f**-treated HCT-116 and LoVo cell cultures (10 µg/mL, for 24 h incubation), using qRT-PCR analysis. As shown in Figure 4, the expression levels of this gene were upregulated by 3.731- and 6.071-fold (in HCT-116), and by 2.6026- and 6.9340-fold (in LoVo) after treatment by **3a** and **3f**, respectively, as compared to the negative control culture.

Furthermore, in order to confirm the implication of these compounds in the apoptotic pathways trigged by caspases, the proteins levels of caspases 8 and 9 were evaluated using Western blot analysis for the HCT-116 and LoVo carcinoma cells lysates after treatment with compounds **3a** and **3f** (50 µg/mL for 48 h); the untreated cells were used as a negative control.

HCT-116 and LoVo carcinoma cells lysates were prepared for the Western blot analysis of caspase-8 and caspase-9.

**Figure 5:**
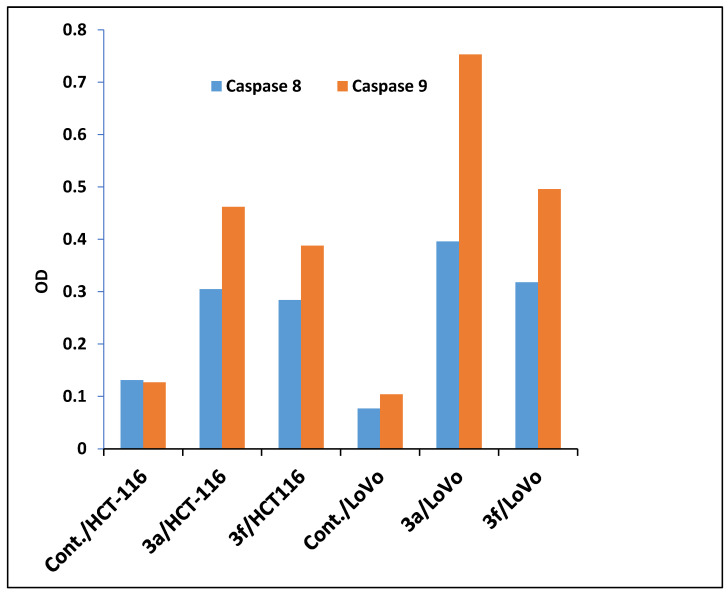
Expressional levels of caspase-8 and caspase-9 in lysates of HCT-116 and LoVo carcinoma cells following treatment with 50 µg/mL of the compounds **3a** and **3f** for 48 h.

**Figure 6:**
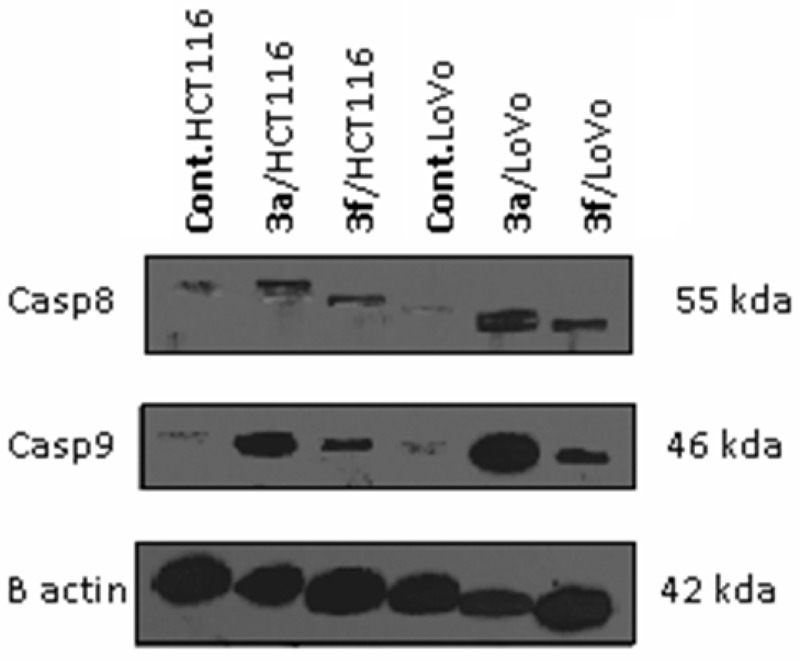
Effects of **3a** and **3f** compounds on protein expressional levels of caspases-8 and 9 after a 48 h treatment of the cells with 50 µg/mL of each compound via immunoblotting, using polyclonal antibodies against caspases -8 and -9. β-Actin was used as the protein loading control.

Compared to the negative control (untreated cells), compounds **3a** and **3f** induced higher expressional levels of caspase-9 with the HCT-116 and LoVo cells than that of caspase-8 in both cultures. The increase in the expressional level of caspase 8 ranged between 2.3 and 2.2 times in the lysates of HCT-116, and between 5.1 and 4.1 times in the lysates of the LoVo cells following a 48 h treatment with **3a** and **3f**, respectively, whereas the expression levels of caspase-9 increased by 3.6 and 3.1 times in the lysates of HCT-116, and between 7.2 and 4.8 times in the lysates of the LoVo cells (Figure 5 and Figure 6). Thus, both proteins were more activated in the cultures of LoVo cells than those of the HCT-116 cells. These results clearly demonstrate that compounds **3a** and **3f** are apoptogenic molecules that are able to induce apoptosis in HCT-116 and LoVo colon cancer cells, predominantly through the intrinsic pathway. Indeed, the caspase-8 levels confirmed that apoptotic cell death caused by **3a** and **3f** was triggered through the extrinsic-mediated pathway, whereby the ligand–receptor binding activated caspase-8. The mitochondrial intrinsic-mediated pathway of apoptosis is also activated in HCT-116 and LoVo-treated cells through the release of high levels of caspase-9 [81]. Both pathways eventually meet up and lead to the hierarchical activation of caspases -3, 6 and 7, which are responsible for the morphological changes in apoptotic cells.

##### Annexin-V Externalization Induced by Compounds **3a** and **3f**

**Figure 7:**
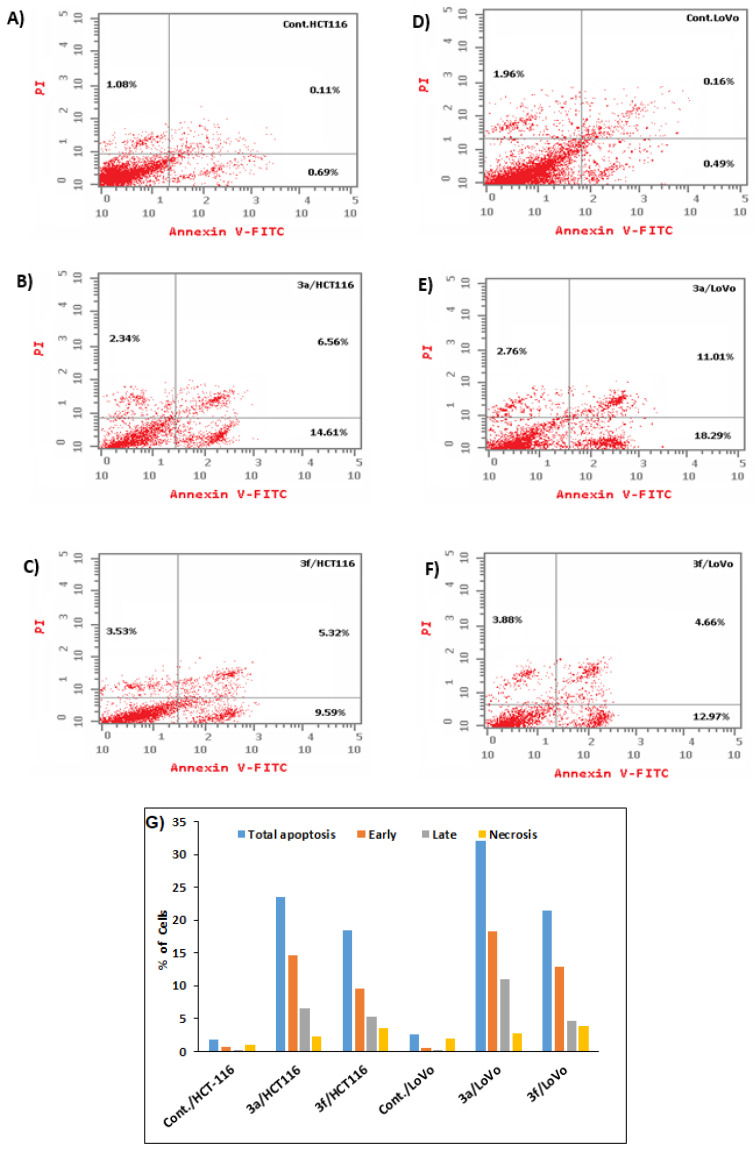
Apoptosis rates of HCT-116 and LoVo cells treated with 50 µg/mL of compounds **3a** and **3f,** detected via flow cytometry, after 48 h treatment (**A**) Untreated HCT-116 cells; (**B**,**C**) treated HCT-116 cells with 50 µg/mL of compounds **3a** and **3f**, respectively. (**D**) Non treated LoVo cells; (**E**,**F**) LoVo cells treated with 50 µg/mL of compounds **3a** and **3f**, respectively. (**G**) Quantification of the apoptosis rate in HCT-116 and LoVo cells undergoing early and late apoptosis detected via Annexin V-FITC/PI dual-staining flow cytometry.

In order to examine the effect of compounds **3a** and **3f** on the apoptosis of HCT-116 and LoVo carcinoma cells, the Annexin-V-FITC probe was used and flow cytometry analyses were performed (Figure 7).

The percentages of the early apoptotic cells (Annexin-V positive and propidium iodide, PI negative) and late apoptotic cells (both Annexin-V and PI positive) were then calculated. Annexin V-PI staining was also performed to distinguish apoptotic and necrotic cell deaths. The data showed that compounds **3a** and **3f** in each cell line caused more apoptotic cell death rather than necrosis (Figure 7G), since necrotic cells represent less than 5% of the total cells’ population. The late apoptotic percentages of the HCT-116 cells was increased to 6.56% (Figure 7B) and 5.32% (Figure 7C) after treatment with **3a** and **3f**, respectively, for 48 h, compared with the untreated cells (Figure 7A).

Similarly, the late apoptotic percentages of the LoVo cells treated with **3a** and **3f** were increased to 11.01% (Figure 7E) and 4.66% (Figure 7F), respectively, while the percentage of the untreated LoVo cells was only 0.16% (Figure 7D). The early apoptotic populations were augmented to 14.6% (Figure 7B) and 18.29% (Figure 7E) for the HCT-116 and LoVo cells treated with compound **3a**, respectively. After the 48 h treatment with **3f**, the percentages of the early apoptotic population of the HCT-116 and LoVo cells reached 9.59% (Figure 7C) and 12.97% (Figure 7F), respectively. These observations clearly imply that compounds **3a** and **3f** effectively induced apoptosis in both tested colon carcinoma cell lines.

##### Compounds **3a** and **3f** Induced Apoptosis in HCT-116 and LoVo Carcinoma Cells Associated with Cell Cycle Arrest

The viability of the cells is maintained by a complete cell cycle life, which is generally developed in the order of the G1-S-G2-M phases. When DNA integrity is impaired by certain factors, cells cannot pass through the G1/S phase and/or the G2/M detection points, and cell proliferation is eventually blocked [82].

**Figure 8:**
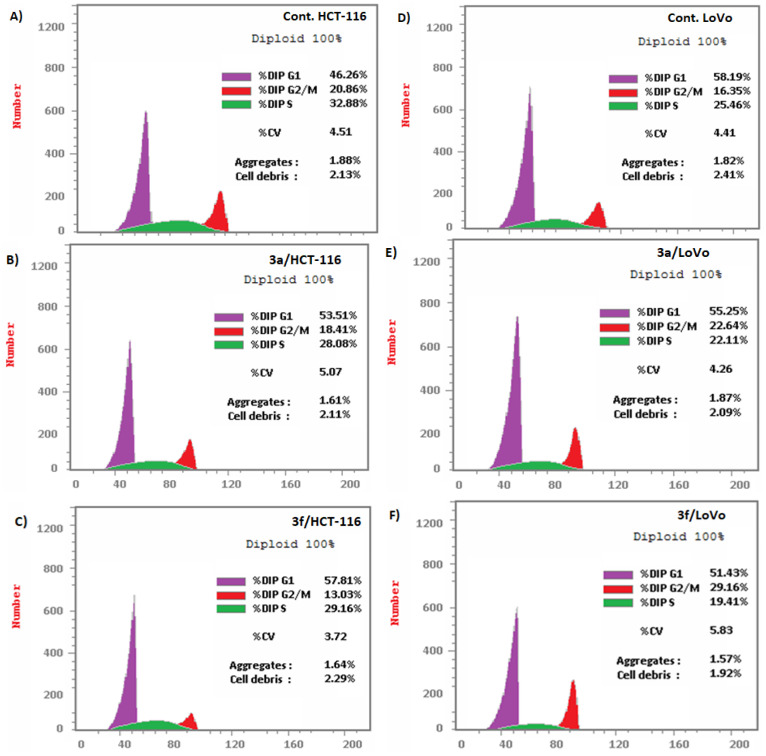
Flow cytometry analysis of cell cycle distribution changes of HCT-116 and LoVo cells treated with 50 µg/mL of compounds **3a** and **3f** over 48 h. (**A**) Control HCT-116; (**B**) treated HCT-116 with **3a**; (**C**) treated HCT-116 with **3f**; (**D**) control LoVo; (**E**) treated LoVo with **3a**; (**F**) treated LoVo with **3f**.

**Figure 9:**
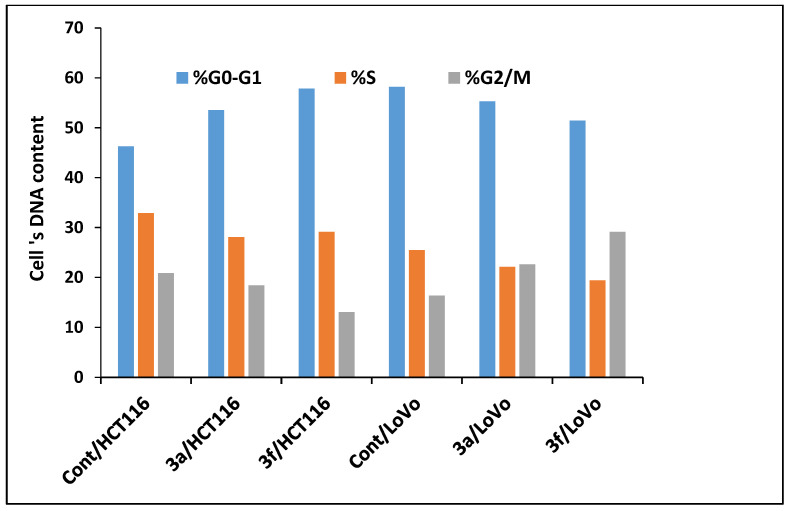
Quantification of the cell cycle distribution of HCT-116 and LoVo cells.

In the current study, the cell cycle distribution of the treated HCT-116 and LoVo carcinoma cells with compounds **3a** and **3f** was assessed by labeling the cell’s DNA with PI stain in order to determine if the induction of apoptosis in carcinoma cells could be related to the cell cycle arrest (Figure 8A–F). The intensity of the PI stain is proportional to the cell’s DNA content (Figure 9). As shown in Figure 8, there was a significant G1 phase arrest in the HCT-116 cells after the 48 h treatment with **3a** and **3f**, where the percentage of the cells at this phase significantly increased from 46.26% (untreated negative control, Figure 8A) to 53.51% and 57.81%, respectively, as shown in Figure 8B,C. Likewise, for the LoVo cell derivatives, **3a** and **3f** induced cell cycle arrest in the G2/M phase, wherein the percentage of the cells’ population in the G2/M phase increased to 22.64% (Figure 8E) and 29.16% (Figure 8F), respectively, compared to the untreated cells (16.35%). The ability of the tested compounds (**3a** and **3f**) to block the cell cycle progression in LoVo and HCT-116 cells confirmed their anticancer potential against these colon carcinoma cell lines and could explain the mechanism of the observed cytotoxicity.

### 2.3. Molecular Docking Simulations

In order to confirm the results concluded from the in vitro enzymatic inhibitory assays, molecular docking studies were performed. Thus, the relative binding affinities, binding interactions and conformations of the most active candidates within the active sites of their target proteins were identified.

#### 2.3.1. Docking against Human Pancreatic Alpha-Amylase (HPA; PDB ID: 3BAJ)

Representative examples for the most active AA inhibitors from the **3a**–**h** and **5a**–**h** series, namely **3c**, **3f**, **3h**, **5a**, **5f** and **5h**, as well as the reference inhibitor quercetin and the co-crystal ligand acarbose, were docked against human pancreatic alpha-amylase (PDB ID: 3BAJ) [83] using AutoDock 4.2.2 with a Lamarckian genetic algorithm-implemented program suite.

**Figure 10:**
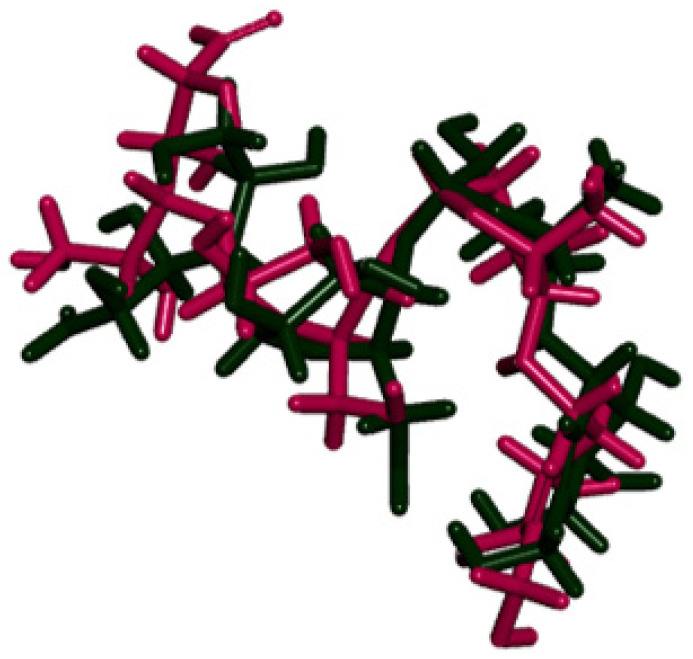
The validation of the accuracy and performance of the docking protocol by redocking **acarbose** ligands in pink and green within the active site of HPA.

Initially, the docking protocol was validated by redocking the co-crystal ligand, **acarbose**, within the active site, with a cutoff of root mean square positional deviation (RMSD) < 2 Å (Figure 10) [84].

**Figure 11:**
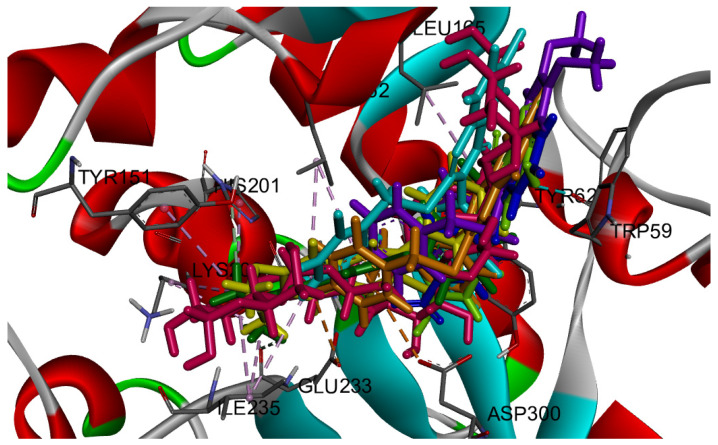
Three-dimensional docked poses of **3c** (green), **3f** (blue), **3h** (yellow), **5a** (orange), **5f** (cyan), **5h** (purple), quercetin (lime) and acarbose (pink) within the active site of HPA (PDB ID: 3BAJ).

The analysis of the results of the docking simulations clearly showed that the studied compounds fitted nicely within the active site and formed hydrogen bonds, Van der Waals, π–π stacking and alkyl and π–alkyl interactions with the active site amino acid residues (Figure 11). Their binding free energies (Table 8) were ranged from −8.4 to −9.1 kcal/mol as compared to −9.3 and –5.4 kcal/mol for quercetin and acarbose, respectively, indicating a considerable affinity between the docked quinazolinones and the enzyme alpha-amylase.

**Figure 12:**
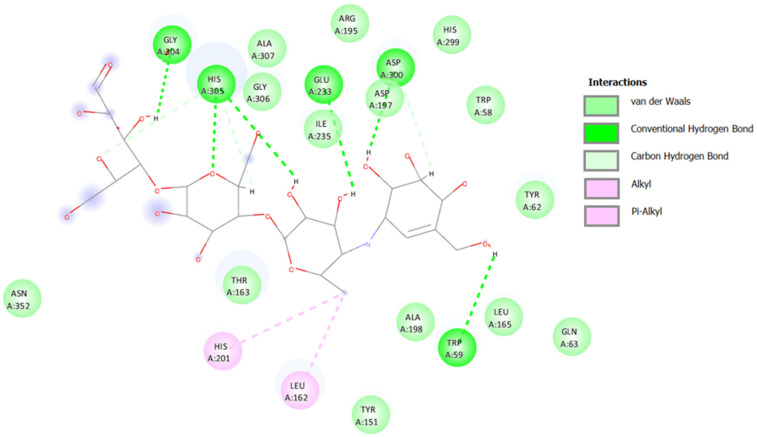
Two-dimensional docked conformation of acarbose at the active site of HPA (PDB ID: 3BAJ).

As shown in Figure 12, acarbose is stabilized in the active catalytic site by six H-bonds, which were formed with the amino acid residues Trp59, Glu233, Asp300, Gly304 and His305 (two interactions). Additionally, acarbose exhibited alkyl interactions with Leu162 and His201.

**Figure 13:**
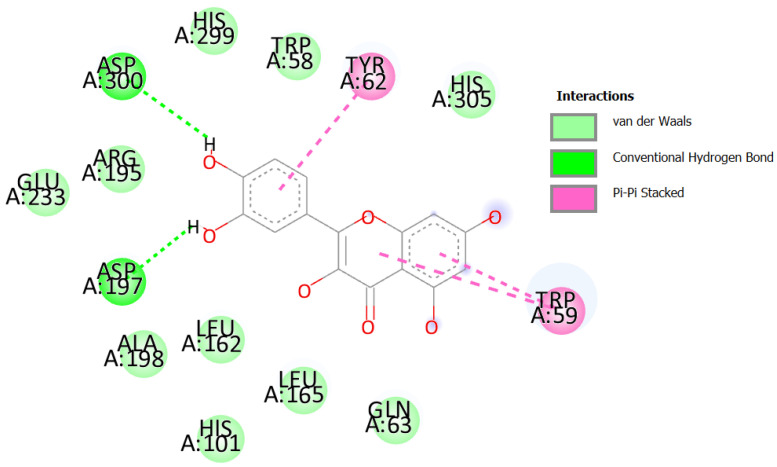
Two-dimensional docked conformation of **quercetin** and the main interactions stabilizing it at the active site of HPA (PDB ID: 3BAJ).

With regard to quercetin, its 3,4-dihydroxyl groups at the terminal phenyl ring formed two hydrogen bonds with the active site residues Asp197 and Asp 300; additionally, the aromatic rings formed π–π stacking with the Trp59 and Tyr62 residues (Figure 13).

**Figure 14:**
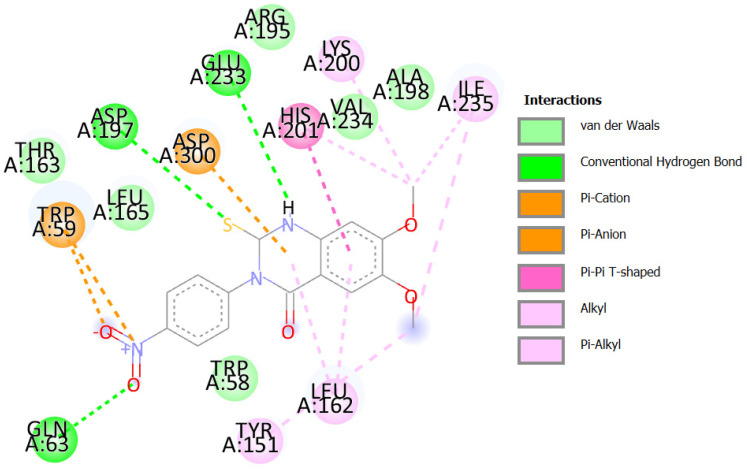
Two-dimensional docked conformation of the best compound, **3h**, and the main interactions stabilizing it at the active site of HPA (PDB ID: 3BAJ).

Among the docked ligands, **3h** manifested the strongest binding affinity to the target enzyme, with the highest binding free energy value. Compound **3h** established π-π stacking interactions with the His201 residue, π–anion and π–cation interactions with Trp59, as well as three hydrogen bonds with amino acid residues Gln63, Asp197 and Glu233. Further stabilizing interactions include the alkyl interactions with amino acid residues Lys200, His 201 and Ile235, as well as the π–alkyl interactions with Leu162 (Figure 14).

Altogether, it can be concluded that these docking simulations affirmed the results of the in vitro AA inhibitory assay, and thereby both studies showed the promising nature of the studied quinazolinone derivatives for alpha-amylase inhibitory activity.

#### 2.3.2. Docking against Recombinant Human Lysosomal Acid-Alpha-Glucosidase (rhGAA, PDB ID: 5NN5)

Molecular docking simulations were performed against the recombinant human lysosomal acid-alpha-glucosidase (rhGAA, PDB ID: 5NN5) [85] using derivatives **5a**, **3h** and **5h**, which exerted lower IC_50_ values, as well as compounds **3c** and **3f**, which showed slightly higher IC_50_ values as compared to quercetin.

**Figure 15:**
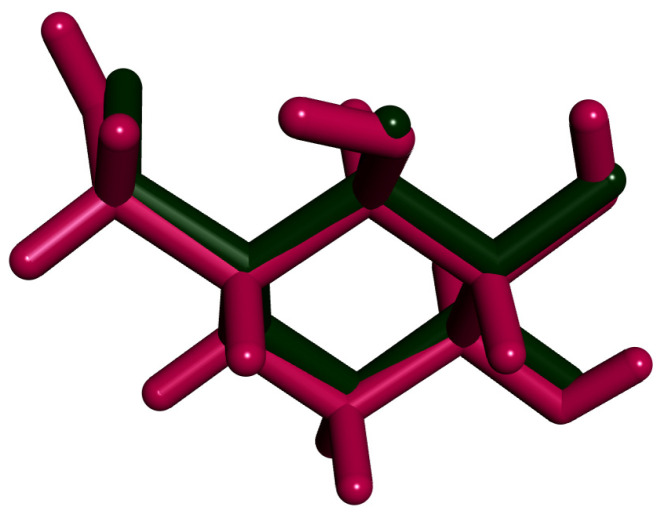
The validation of the accuracy and performance of the docking protocol by redocking 1-deoxynojirimycin (pink) in the active pocket of rhGAA, and the calculation of the RMSD value as compared to the native conformation (green).

First, the docking protocol was validated by redocking the native ligand 1-deoxynojirimycin (DNJ) at the active pocket with the RMSD deviation < 2 Å, implying the accuracy of the docking procedures (Figure 15).

**Figure 16:**
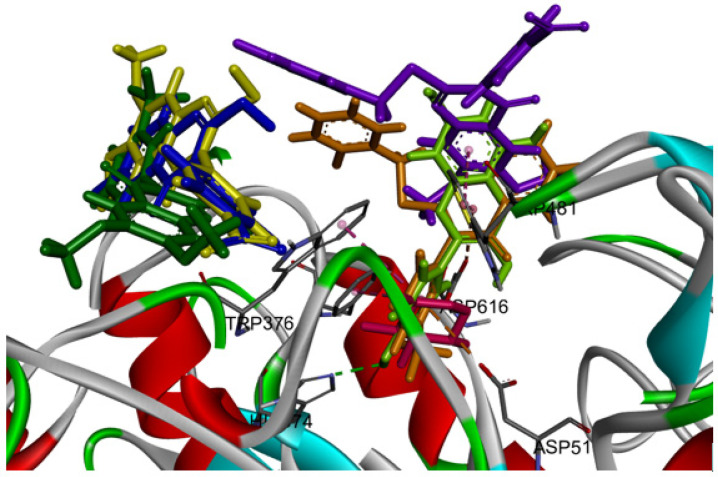
Three-dimensional docked poses of ligands **3c** (green), **3f** (blue), **3h** (yellow), **5a** (orange), **5h** (purple), quercetin (lime) and 1-deoxynojirimycin (pink) within the active site of rhGAA (PDB ID: 5NN5).

The docking results showed that ligands **3c**, **3f** and **3h** bound differently from **5a**, **5h**, quercetin and DNJ (Figure 16).

**Figure 17:**
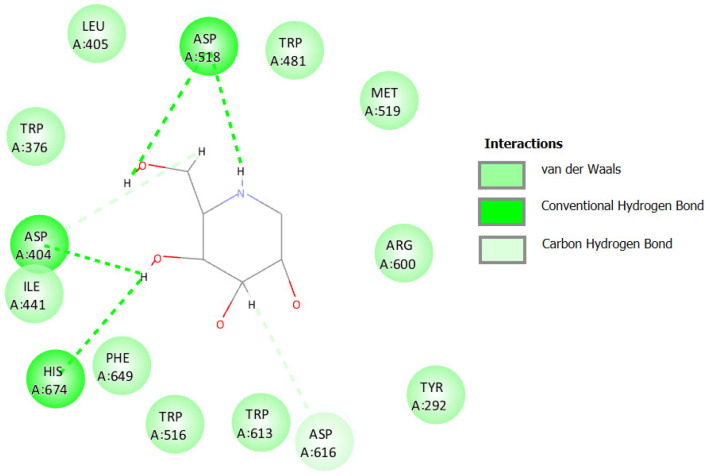
Two-dimensional docked conformation of 1-deoxynojirimycin (DNJ) at the active site of rhGAA (PDB ID: 5NN5).

Thus, the analysis of the 2D docked conformation of the native ligand DNJ within the active pocket of rhGAA (binding free energy = −5.7 Kcal/mol) revealed the establishment of four H-bonds with amino acid residues Asp404, Asp518 (two interactions) and His674. Further stabilizing interactions were provided via Van der Waals forces with Asp404 and Asp616 (Figure 17).

**Figure 18:**
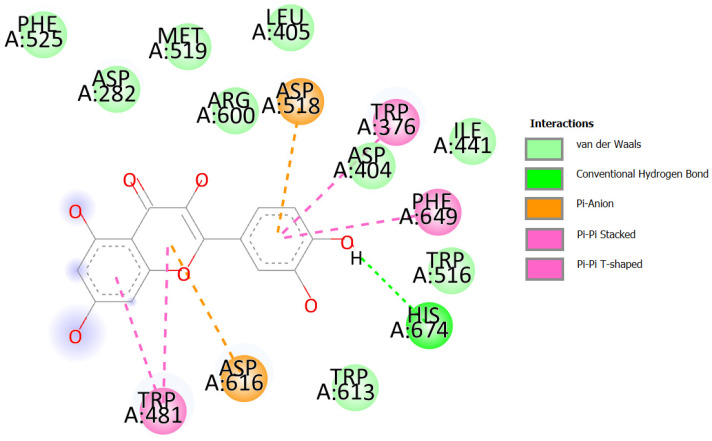
Two-dimensional docked conformation of **quercetin** and the main interactions stabilizing it at the active site of rhGAA (PDB ID: 5NN5).

Quercetin showed strong interactions and fitted inside the active pocket, with a binding energy of −7.0 kcal/mol (Table 8). The para-hydroxyl group at the 2-phenyl substituent formed one hydrogen bond with His674. Moreover, the aromatic rings of quercetin showed π–π stacking with the Trp376, Trp481 and Phe649 amino acid residues (Figure 18).

**Figure 19:**
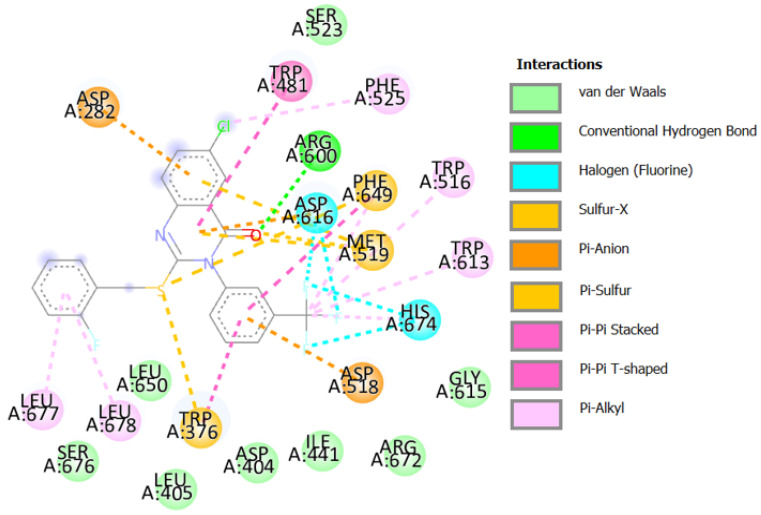
Two-dimensional docked conformation of compound **5a** and the main interactions stabilizing it at the active site of rhGAA (PDB ID: 5NN5).

Among the studied quinazolinones, derivative **5a** showed a strong affinity to the active site of the enzyme and fitted properly, with the highest binding energy value of −9.0 kcal/mol (Table 8). Its oxo group formed a hydrogen bonding interaction with amino acid residue Arg600. Other stabilizing interactions were provided via the π–π stacking with Trp376, Trp481 and Phe649 residues, in addition to several π–S, halogen and π–alkyl interactions (Figure 19).

Collectively, the findings from the docking simulations correlated well to the results of the in vitro AA and AG inhibition experiments.

#### 2.3.3. Docking against Lactate Dehydrogenase A (LDHA, PDB code: 1I10)

The 3D structure of LDHA was modelled using the known structure of human muscle LDHA (PDB code: 1I10), which comprises four subunits [86].

**Figure 20:**
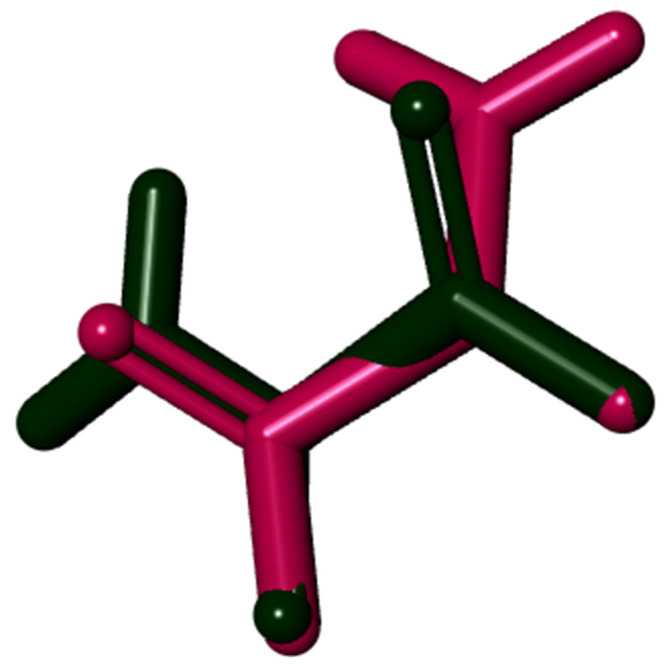
The validation of the accuracy and performance of the docking protocol by redocking oxamate (pink), and the calculation of the RMSD value as compared to native conformation (green).

The docking method was validated by removing and redocking the co-crystallized ligand (oxamate) in order to determine the ability of the AutoDock v. 4.2.2 program to reproduce the orientation and position of the native ligand observed in the experimentally crystallized protein structure. As shown in Figure 20, the redocked conformation of the ligand oxamate is superimposed on the co-crystallized one and the RMSD deviation is <2 Å, which ensures the accuracy and reliability of the docking results.

**Figure 21:**
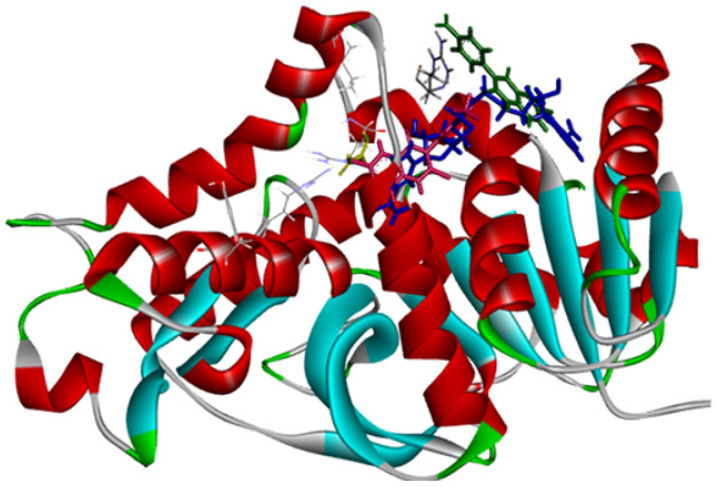
Three-dimensional docked conformation of compounds **3a** (pink), **3g** (green), oxamate (yellow) and NADH (blue) at the active site of LDHA (PDB code: 1I10).

Thereafter, the most promising LDHA inhibitors, **3a** and **3g**, as well as the reference inhibitor, oxamate, were docked inside the active site of lactate dehydrogenase A (PDB code: 1I10) [86] using the Autodock v. 4.2.2 program (Figure 21).

**Figure 22:**
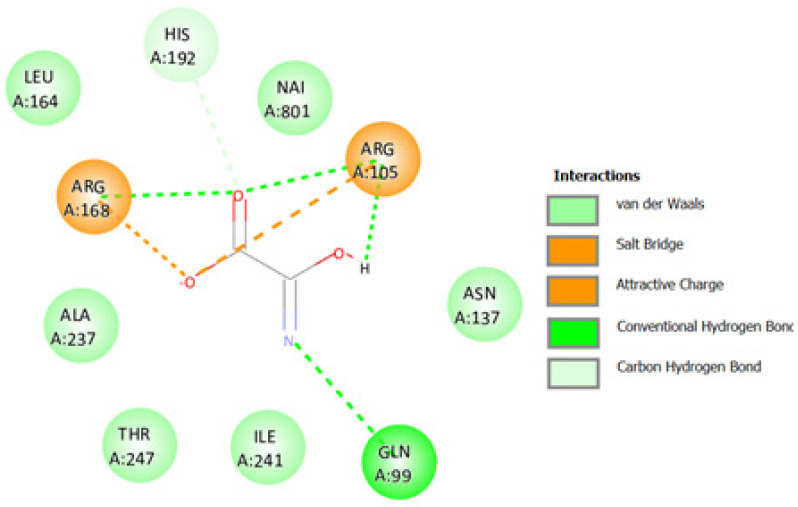
Two-dimensional docked conformation of **oxamate** at the active site of LDHA (PDB code: 1I10).

Previous studies have shown that oxamate, a competitive analogue of pyruvate, formed hydrogen bonds with the Arg105 guanidinium group, the 3′-OH-group of the NADH nicotinamide and the backbone oxygen of Ala97 [87]; thus, this region was identified as the active site of the LDHA (Figure 21). Among these, the interaction with Arg105 in the loop plays an essential role in stabilizing the transition state during the substrate conversion. Our results also showed three hydrogen bonding interactions of oxamate with Gln99, Arg105 and Arg168 (Figure 22).

When docked into the same active site, compounds **3a** and **3g** occupied the same pocket in proximity with the NADH, with different interactions.

**Figure 23:**
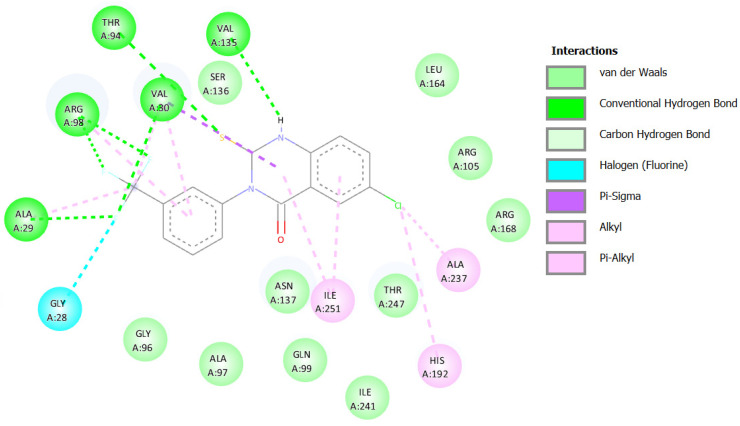
Two-dimensional docked conformation of compound **3a** at the active site of LDHA (PDB code: 1I10).

Regarding compound **3a**, it was stabilized in the active site by establishing six hydrogen bonding interactions; two of them were between the thioxo and NH groups with Thr94 and Val135, respectively. Additionally, the trifluoromethyl group formed four hydrogen bonds with the Ala29, Val30 and Arg98 (two interactions) amino acid residues. The distal phenyl ring fitted onto the lipophilic pocket, forming a π–alkyl stacking interaction (Figure 23). These interactions are different from those observed in previous studies [87], which may account for its reduced inhibitory efficiency as compared to oxamate. However, the binding free energy of compound **3a** was −9.2 kcal/mol, indicating its significant affinity to the enzyme.

**Figure 24:**
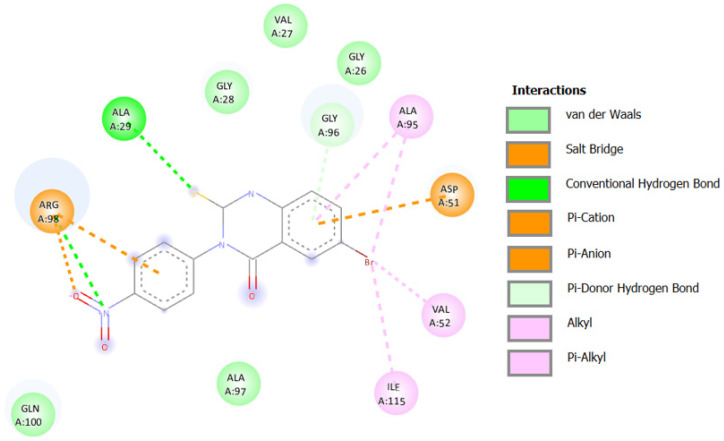
Two-dimensional docked conformation of compound **3g** at the active site of LDHA (PDB code: 1I10).

On the other hand, compound **3g** fitted within the catalytic active site, with a binding free energy of −5.9 kcal/mol, by forming only two hydrogen bonds with the Ala29 and Arg98 residues (Figure 24).

#### 2.3.4. Docking against COX-2 (PDB ID: 3LN1)

**Figure 25:**
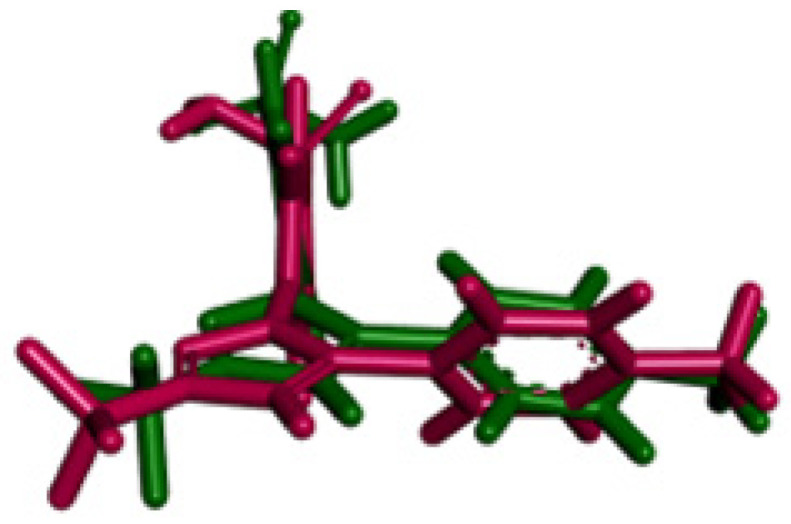
The validation of the accuracy and performance of the docking protocol by redocking celecoxib (pink), and the calculation of the RMSD value as compared to the native conformation (green).

Moreover, molecular docking studies of compounds **3a** and **3g** were carried out in order to identify their selectivity and interactions with the target enzyme COX-2 (PDB ID: 3LN1) [88] after reproducing the conformation of celecoxib (shown in pink) with the RMSD deviation < 2 Å, as compared to the native conformation (shown in green) (Figure 25).

**Figure 26:**
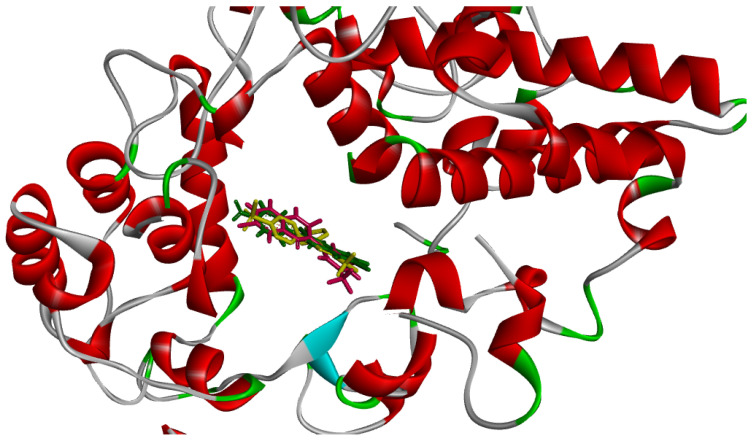
Three-dimensional docked conformation of compounds **3a** (pink), **3g** (green) and celecoxib (yellow) at the active site of COX-2 (PDB code: 3LN1).

The molecular docking studies revealed that both the quinazolinone derivatives, **3a** and **3g**, showed good interactions with the amino acid residues in the enzyme active site, with binding energies of −9.4 and −9.0 kcal/mol, respectively, as compared with the standard drug celecoxib (−7.1 kcal/mol) (Figure 26).

**Figure 27:**
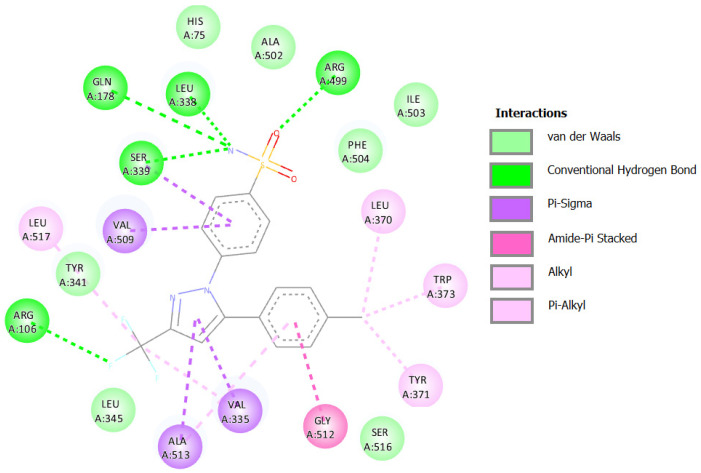
Two-dimensional docked conformation of celecoxib at the active site of COX-2 (PDB ID: 3LN1).

The sulfonamide group of celecoxib showed hydrogen bonding with Gln 178, Leu 338, Ser 339 and Arg 499. The pyrazole ring and phenyl ring showed π–sigma interactions with Ala513, Val335 and Val509 Arg 106 (Figure 27).

**Figure 28:**
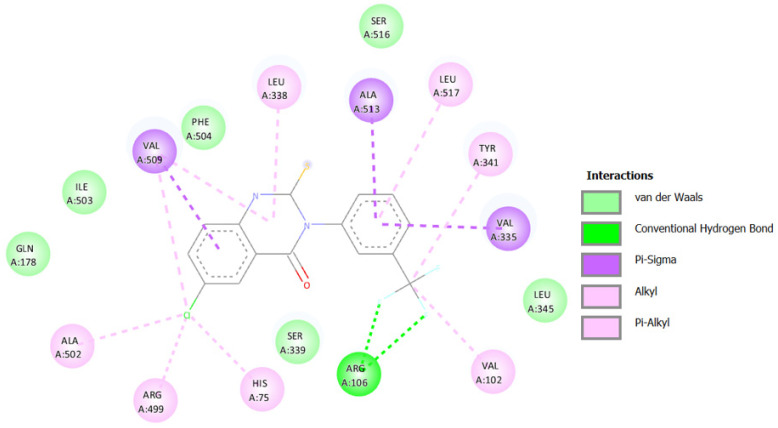
Two-dimensional docked conformation of compound **3a** at the active site of COX-2 (PDB ID: 3LN1).

**Figure 29:**
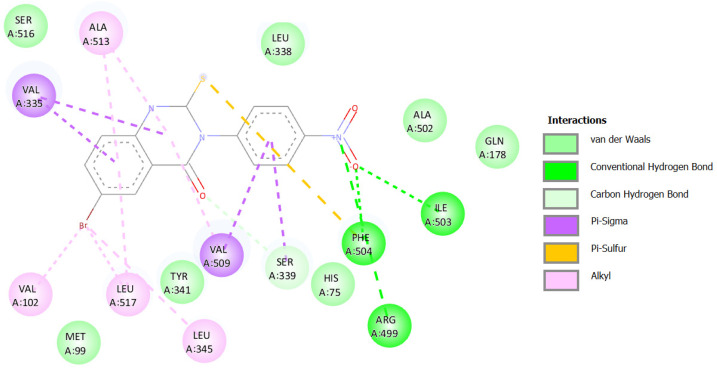
Two-dimensional docked conformation of compound **3g** at the active site of COX-2 (PDB ID: 3LN1).

Compounds **3a** and **3g** occupied the same pocket, but with different interactions. Derivative **3a** showed two hydrogen bonds with Arg106 (Figure 28), whereas **3g** showed three hydrogen bonds with Arg499, Ile503 and Phe504 (Figure 29). Thus, it can be concluded that these interactions contributed to the measured inhibitory activity of these quinazolinone analogues.

### 2.4. In Silico Physicochemical, Medicinal and ADMET Predictions

The data for all the synthesized compounds are available in the Supplementary Materials.

#### 2.4.1. Physicochemical Properties of the Most Biologically Active Candidates

The physicochemical, medicinal chemistry and ADMET properties of all synthesized compounds were predicted (Supplementary Materials) using ADMETLab2.0 [89].

Considering derivatives **3a** (antioxidant, COX-2 inhibitor, LDHA inhibitor, AA inhibitor, cytotoxic agent), **3c** (AA inhibitor), **3f** (AA inhibitor, cytotoxic agent), **3g** (antioxidant, COX-2 inhibitor, LDHA inhibitor), **3h** (AA inhibitor, AG inhibitor), **5a** (antioxidant, AA inhibitor, AG inhibitor), **5b** (AA inhibitor), **5c** (AA inhibitor), **5d** (AA inhibitor), **5e** (AA inhibitor), **5f** (AA inhibitor) and **5h** (AA inhibitor, AG inhibitor), which exhibited promising biological activities, their molecular descriptors were predicted as presented in Table 9.

#### 2.4.2. Medicinal Chemistry of the Most Biologically Active Candidates

The same set of compounds were further evaluated for their suitability in medicinal chemistry using a number of well-known measures, rules and filters.

##### The Quantitative Estimate of Drug-likeness (QED) Score

This is a measure of drug-likeness based on the concept of desirability, estimated from eight properties, including MW, LogP, nHA, nHD, TPSA, nRot, the number of aromatic rings (NAr) and the number of alerts for undesirability. This descriptor ranks compounds having a mean QED score of ≥ 0.67 as attractive; on the other hand, unattractive compounds would have a QED score of 0.67 > QED ≥ 0.34. Moreover, unattractive and too complex compounds would have a mean QED score of < 0.34 [90]. The predicted data indicated that **3a** (0.641), **3c** (0.64), **3f** (0.441), **3g** (0.419) and **3h** (0.437) would be ranked as unattractive, whereas **5a** (0.197), **5b** (0.21), **5c** (0.136), **5d** (0.233)**, 5e** (0.179), **5f** (0.131), and **5h** (0.153) could be categorized as unattractive and too complex compounds.

##### Lipinski’s Rule of Five Filter

Lipinski’s rule of five [91] is a widely used filter to evaluate a compound’s drug-likeness physicochemical characteristics and its suitability for oral administration. The four parameters of this rule, which are associated with absorption and/or permeability, include the molecular weight, the number of H-bond acceptors (HBAs, expressed as sum of Os and Ns), the number of hydrogen bond donors (HBDs expressed as the sum of HOs and HNs) and the LogP value, which is a measure of a compound’s hydrophilicity and it represents the logarithm of its partition coefficient between *n*-octanol and water (log(c_octanol_/c_water_))were predicted using ADMTLab 2.0.

As presented in Table 9, all the studied compounds were found to have molecular weights with a range of 333.00 ~ 483.07 Da, which are within the permissible limit (≤ 500), except for compounds **5b** (523.96), **5c** (517.09), **5e** (597.92) and **5f** (530.97).

The number of HBAs ranged from three to eight; thus, they do not exceed the limit (≤ 10). Similarly, the number of HBDs ranged from zero to one, which complies with the acceptance value (≤ 5). The LogP values were predicted to span from 2.011 to 6.228 (limit ≤ 5); thus, all studied compounds would possess desirable lipophilic characteristics, except for derivatives **5a** (5.561), **5b** (6.153), **5d** (6.039), **5e** (6.228) and **5f** (5.3438).

Overall, derivatives **3a**, **3c**, **3f**, **3g**, **3h** and **5h**, which fulfilled the thresholds of the Lipinski Ro5, as well as quinazolinones **5a**, **5c** and **5d**, which exhibited one violation, would be expected to be absorbed by the intestinal walls, whereas derivatives **5b**, **5e** and **5f**, which showed two violations (Table 10), would be poorly absorbed by the intestinal walls, as indicated by the rule [91].

##### Pfizer Rule

According to this rule, the combination of a high LogP with a low TPSA increases the likelihood of adverse outcomes; this is due to a low polar surface area (TPSA < 75), and a higher LogP (LogP > 3) increases the ability of a compound to cross biological membranes and distribute widely into off-target tissue compartments [92]. Therefore, the high/low thresholds for LogP and TPSA have cutoffs of 3.0 and 75, and when both risk factors are present, the compound is likely to be toxic. Thus, according to data presented in Table 9, only compounds **3c**, **3f**, **3g**, **3h, 5c**, **5f** and **5h** complied with the Pfizer rule (Table 10).

##### GlaxoSmithKline (GSK) Rule

This rule highlights the importance of a lower molecular weight and LogP physicochemical properties to obtain improved ADMET parameters. The cutoff limits are MW ≤ 400 and LogP ≤ 4 [93]. Thus, **3a**, **3c**, **3f**, **3g** and **3h**, satisfied this rule.

##### Golden Triangle

This visualization tool [94] was developed to aid in identifying metabolically stable, permeable and potent drug candidates, which would have values of 200 ≤ MW ≤ 500, and −2 ≤ LogD ≤ 5. As per the data in Table 9, compounds **5b**, **5c**, **5e** and **5f** violate this rule (Table 10); thus, they are expected to have unfavorable ADMET profiles.

The ADMET profiles of the dual AA and AAG inhibitors, **3h**, **5a** and **5h**, are discussed in detail in Section 2.4.3.

##### Pan-Assay Interference Compounds (PAINS) Filter

This filter aids in detecting substructural features that may interfere with bioactivity detection technology that are frequently found in problematic compounds, making them give false positive results in bioactivity assays for a number of reasons [95,96,97]. Also, compounds bearing interference moieties tend to have therapeutic limitations due to these moieties often being associated with reactivity, toxicity or metabolic liability [97]. Consequently, the screening results showed that none of studied compounds were flagged to contain PAINS alters.

#### 2.4.3. Drug Metabolism and Pharmacokinetic (DMPK) Analysis for the AA and AG Dual Inhibitors: **3h**, **5a** and **5h**

Because undesirable ADMET profiles, Absorption, Distribution, Metabolism, Excretion and Toxicity, are the main reasons for high attrition rates in drug development, computer-aided models are being relied on for the prediction of these properties for drug candidates in the early stages of drug discovery programs, thus saving extensive efforts and reducing cost and duration as compared to in vivo models [98]. Therefore, the ADMET parameters were predicted for the AA and AG dual inhibitors, **3h**, **5a** and **5h**, using the ADMETlab 2.0 server [89], which was freely accessed on 22^nd^ August 2023 at https://admetmesh.scbdd.com/.

For simplification, the computed values for all descriptors will be listed in the order of **3h**, **5a** and **5h**, respectively.

##### Absorption Prediction

The absorption profile of a drug is influenced by specific biochemical characteristics, including the water/lipid solubility, bioavailability and permeability across barriers—including the gastrointestinal tract lining, which can be predicted as human intestinal absorption (HIA)—as well as the interaction with transporters and metabolizing enzymes in the gut wall.

Solubility (LogS)

The computed descriptor used to assess the aqueous solubility is LogS; the logarithm of molar solubility is expressed as Log mol/L, and the empirical decision indicated that compounds with values falling within the range from −6 to 0.5 log mol/L would be considered proper; thus, derivative **3h** is expected to show a good water solubility, with a LogS value of −3.934 (Table 9), whereas derivatives **5a** and **5h** would be insoluble, with LogS values of −7.104, and −6.875, respectively; accordingly, they might not be orally bioactive.

Solubility is essentially influenced by the presence of molecular entities (NH_2_, OH, NO_2_ and OCH_3_) that are capable of H-bond formation with water molecules or those conferring lipid solubility (aromatic groups (Cl, Br and I; the effect of F can vary). Thus, the order of solubility based on the computed LogS values is as follows: **3h** > **5h** > **5a**, which is consistent with the type of substituents and functional groups incorporated in each of them, as **3h** possessed NO_2_, OCH_3_ and NH groups, **5h** possessed NO_2_, OCH_3_ and Cl, and **5a** possessed Cl, CF_3_ and F.

Human Intestinal Absorption (HIA)

The HIA of an oral drug is an essential prerequisite for its apparent efficacy; in addition, it can be used as an alternative indicator for oral bioavailability. The molecules are classified as category 0 (HIA−) with a HIA of > 30%, or as category 1 (HIA+) with a HIA of < 30%, with the output value indicating the probability of being of HIA+ within the range of 0 to 1. The estimated scores were 0.008, 0.003 and 0.004, respectively, implying that they are not expected to belong to HIA+ category.

The Human Oral Bioavailability (HOB)

Human oral bioavailability (HOB) is an important parameter used to measure the amount of a drug that actually enters circulation after ingestion. The high oral bioavailability of a drug would reduce the amount of administration needed to achieve the expected pharmacological action, which can reduce the side effects and toxicity risks brought by the drug.

**Figure 30:**
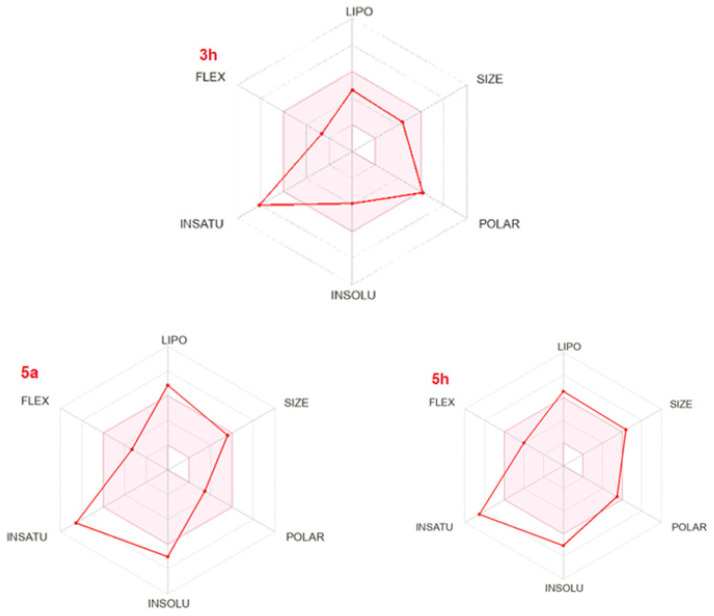
The bioavailability radar hexagons for derivatives **3h**, **5a** and **5h**, predicted using the SwissADME web tool. The pink area represents the optimal range for each property and the red lines define the oral bioavailability properties of the studied compounds.

The oral bioavailability radar plots shown in Figure 30 were predicted with the SwissADMET web tool [95]. This graph is based on six physicochemical descriptors arranged on the corners of a hexagon, including insolubility (LogS), polarity (TPSA), size (MW), flexibility (FLEX, based on number of nRot/nRig), lipophilicity (Lipo, based on LogPoctanol/water) and unsaturation. The colored zone is the suitable physicochemical space for oral bioavailability.

As shown in the bioavailability radar hexagons (Figure 30), the three compounds possessed suitable sizes, flexibilities and polarities. However, all of them demonstrated high unsaturation fractions, indicated by the off-shoot of the INSATU vertex, which may affect their solubility since it was hypothesized that increasing the sp^3^ fraction might increase the complexity of the molecules, improving their solubility and providing a three-dimensionality that could contribute to greater selectivity and fewer off-target effects [99].

Additionally, compounds **5a** and **5h** possessed unsuitable lipophilicity and solubility characteristics. The link between solubility and lipophilicity is well established; as the LogP increases, solubility decreases on average [93]. Therefore, the order of LogP among the three compounds is as follows: **5a** > **5h** > **3h**, which is the reverse order of solubility.

The Human Oral Bioavailability Factors 20% (F_20%_) and 30% (F_30%_)

Moreover, two cutoffs can be used to assess the HOB; the first is, F_20%_, wherein the molecules with a bioavailability of ≥ 20% are classified as category 0 (F_20%−_), whereas molecules with a bioavailability of < 20% are classified as category 1 (F_20%+_). The output value is the probability of being F_20%+_ within the range of 0 to 1. The estimated scores for the studied compounds were 0.002, 0.002 and 0.001, implying that the compounds have negligible probabilities for being F_20%+_.

The second is F_30%_, whereby the molecules with a bioavailability of ≥ 30% are classified as category 0 (F_30%−_), whereas molecules with a bioavailability of < 30% are classified as category 1 (F_30%+_). The output value is the probability of being F_30%+_ within the range of 0 to 1. The calculated scores were 0.002, 0.003 and 0.004, respectively, implying that the three compounds have negligible probabilities of being F_30%+_.

Permeability

After an orally administered drug is dissolved in the gastro-intestinal tract, it must then be sufficiently permeable through the biological membranes present to enter the systemic circulation. Permeation is often mimicked in laboratories using artificial membrane assays such as the Caucasian colon adenocarcinoma cell line (Caco-2) and the Madin Darby Canine Kidney cell line (MDCK).

With regard to Caco-2 permeability descriptor, the computed values expressed in log cm/s of the studied compounds were −4.737, −5.065, and −4.771, respectively; thus, all of them comply with the respected range for this property (value > −5.15 log cm/s).

Respecting the MDCK permeability as another tool, which is assessed using the apparent permeability coefficient Papp in cm/s, the predicted values were 5.25 × 10^−5^, 1.13 × 10^−5^ and 2.43 × 10^−5^, respectively, whereby the standard limits for Papp in cm/s and their significance are a high permeability of > 20 × 10^−6^, medium permeability of 20–2 × 10^−6^, and low permeability of < 2 × 10^−6^. Thus, all derivatives have a high passive MDCK permeability.

P-glycoprotein (P-gp) Efflux

The P-glycoprotein (P-gp) efflux is considered one of the determinants of oral bioavailability as well as the rate and amount of a drug that diffuses across the basolateral membrane to enter the general circulation. Therefore, P-gp efflux screening is a key step in the early drug discovery stage. Compounds are classified as non-inhibitors (category 0) and inhibitors (category 1), with the output value representing the probability of being an inhibitor within the range of 0 to 1. With regard to **3h**, **5a** and **5h**, they are anticipated to be P-gp inhibitors as per their scores of 0.978, 0.991 and 0.994, respectively. So, these derivatives are expected to show drug–drug interactions (DDIs) with co-administrated P-gp substrates.

With respect to P-gp substrate properties, the compounds may belong to category 0 if they are non-substrates, or to category 1 if they are substrates, with the output value indicating the probability of being a substrate within the range of 0 to 1. The predicted value for the three compounds was 0.001, implying that they have a low probability for being substrates, which is beneficial, since it is known that therapeutic agents which are substrates of P-gp usually exhibit a poor bioavailability due to P-gp blocking their absorption [100]. In addition, they can be considered as safe from having significant DDIs when co-administrated with other Pgp inhibitors.

##### Distribution

After the drug is absorbed, it enters the systematic circulation to be distributed throughout the body, where it is reversibly transferred between various tissues, organs and cells. Thus, this property is essential for the prediction of pharmacodynamic and toxicodynamic properties [101].

Four descriptors were predicted to assess the efficiency of the drug distribution throughout the body: plasma protein binding (PPB), the volume of distribution (VD), the fraction unbound in plasma (fu), and uptake by the blood–brain barrier (BBB).

The Degree of Plasma Protein Binding (PPB)

This refers to the binding of the drug to plasma proteins such as albumin, which significantly influences the distribution of the drug between the plasma and the tissue of interest; hence, it determines the efficacy of a given drug as well as its clearance (Cl).

Generally, only the free or unbound fraction of a drug is active; therefore, if there is little inclination that a drug molecule will bind with plasma proteins (predicted PPB value ≤ 90%), as a consequence, this compound is considered to have a high therapeutic index, meaning that it can circulate more efficiently and freely within the blood stream and hence has access to the target site. In contrast, a compound with a PPB value of > 90 is expected to be poorly distributed. For the studied compounds, the PPB values were calculated to be 84.49, 100.92 and 100.40, respectively; as a consequence, only **3h** is expected to demonstrate a high therapeutic efficiency. It is noteworthy to indicate that many marketed drugs have PPB values of > 90% [102]; thus, for **5a** and **5h**, their high values can be tolerated if they have a high rate of dissociation. Also, this high tendency for PPB can be advantageous, as the plasma–drug complex is considered as a reservoir for free drug concentration whenever it is eliminated from the body, thus prolonging the duration of the drug’s action [102].

The Fraction Unbound in Plasma (Fu)

The Fu is an indicator of the free amount of a drug in the plasma, and it determines a compound’s capability to traverse biological barriers and gain access to target tissues and organs. As a consequence, the more plasma-bound a compound is, the less efficiently it can traverse biological barriers. The standard scores for high, medium and low fractions unbound in plasma are as follows: > 20%; 5–20%; and < 5%, respectively. With regard to the predicated values of the investigated compounds, they are equal to 13.85, 0.66 and 0.55, respectively; thus, again, only derivative **3h** is expected to have a moderate therapeutic efficiency.

Volume of Distribution (VD)

As a drug is absorbed, its distribution throughout the body must be considered, since it determines if the compound will produce a pharmacological response or not. The VD value (L/Kg) can be used to predict the distribution characters for an unknown drug, including its conditions of binding to plasma proteins, its distribution amount in the body’s fluid and its uptake amount in tissues. The optimal range of the VD for proper distribution is between 0.04 and 20 L/Kg, otherwise it is poor. For the studied compounds, the computed VD values were 0.497, 0.686 and 0.132, respectively, implying that they are likely to be well distributed throughout the body’s tissues and will not confined to the plasma.

Penetration of Blood–Brain Barrier (BBB)

This is an important descriptor to anticipate the capability of a compound to distribute into the brain. It is measured as LogBB in cm/s, and if a compound has a LogBB > −1, it will penetrate the BBB and thus, it is classified as category 1 (BBB+); however, it will be poorly distributed to the brain if it has a LogBB of ≤ −1, and will be classified as category 0 (BBB−). The output value is the probability of being BBB+, within the range of 0–1. The calculated values for the specified inhibitors were 0.18, 0.298 and 0.212, respectively; thus, all of them are not expected to penetrate the BBB, implying that they are not associated with CNS toxicity.

##### Metabolism

Drug metabolism is a very complex process, which begins once a compound penetrates the gastrointestinal tract and passes through the portal vein to the liver. Two phases may be involved in metabolizing drugs: phase I, in which reactions such as oxidations, reductions, hydrolysis and dealkylations take place; and phase II, in which conjugation reactions with endogenous substances occur.

Cytochrome P-450 (CYP450) is a superfamily of isoenzymes that catalyze the phase I metabolism. For an orally delivered drug to be distributed beyond the liver to exert its pharmacological action on the target organ, some fraction of this drug must survive the hepatic metabolism. Therefore, the property of being a substrate or an inhibitor to these hepatic enzymes is curial. Also, it may induce DDIs, whereby one drug may enhance the toxicity or reduce the therapeutic effect of another drug. The major CYP450 isoforms, which are responsible for most of the metabolic reactions, include CYP1A2, CYP2C19, CYP2C9, CYP2D6 and CYP3A4.

According to the results presented in the Supplementary Materials, the compounds showed varied probabilities of being substates or inhibitors for these cytochromes. The utilized database classified the compounds in two categories: category 0 for non-substrates/non-inhibitors; and category 1 for substrates/inhibitors. The output values indicate the probability of being a substrate/inhibitor, within the range of 0 to 1.

With respect to CYP1A2, the computed probabilities for **3h** of being an inhibitor and a substrate were 0.124 and 0.97, respectively, indicating that it is a possible substrate for this enzyme. The probabilities for **5a** were anticipated to be 0.885 and 0.238, respectively, indicating that it is a possible inhibitor. Lastly, the computed probabilities for **5h** were 0.245 and 0.826, respectively; thus, it appeared to be a substrate for CYP1A2.

The predicted probabilities of being an inhibitor and a substrate for CYP2C19 were 0.325 and 0.553 (**3h**), 0.062 and 0.929 (**5a**), and 0.894 and 0.177 (**5h**), respectively. Thus, **5a** and **5h** are highly expected to be a substrate and an inhibitor for the CYP2C19 isoform, respectively.

As regards CYP2C9, the measured inhibitor/substrate probabilities were 0.163/0.832 (**3h**), 0.929/0.858 (**5a**), and 0.901/0.904 (**5h**), respectively. Accordingly, all of them have a high probability of being substrates; in addition, **5a** and **5h** appeared to be possible inhibitors for this cytochrome.

Concerning CYP2D6, the measured inhibitor/substrate probabilities were 0.01/0.468 (**3h**), 0.347/0.396 (**5a**), and 0.046/0.69 (**5h**), respectively. Therefore, there is no expected incidence of 2D6 isoenzyme inhibition by **3h** and **5h,** but medium probability for inhibition by **5a**. In addition, there are moderate probabilities for all of them for being 2D6 substates.

Lastly, regarding the inhibitor/substrate probabilities for CYP3A4, they were predicted as follows: 0.277/0.826 (**3h**), 0.567/0.641 (**5a**) and 0.727/0.929 (**5h**), respectively, implying that very high probabilities exist for **3h** and **5h** as substrates and moderate probabilities for **5a** and **5h** as inhibitors.

##### Excretion

Total clearance parameters (CLs), which are expressed as log mL/min/kg, are used to predict the removal rate of a compound from the systemic circulation by all methods: “renal clearance; hepatic clearance via metabolic loss; and loss into milk, sweat and saliva”.

The CLs are inversely proportional to a compound’s in vivo half-life time; thus, a higher CL value implies that a compound is more rapidly removed from the body by any method, which may impact its potency. Consequently, CLs define, together with the volume of distribution, the dosing frequency of a drug [103]. The standard scores in mL/min/kg for high, moderate and low clearance are >15, 5–15 and < 5, respectively.

The calculated CL (ml/min/kg) results were 6.455, 3.838 and 4.054, implying that the clearance for **3h** is moderate, whereas it is expected to be low for **5a** and **5h**.

For the half-life of a drug (T_1/2_ in hour), the database subdivided molecules as category 0 (T_1/2−_) with a T_1/2_ of > 3, and category 1 (T_1/2+_) for molecules with a T_1/2_ of ≤ 3 h. The output value is the probability of being T_1/2+_ with score ranges of 0–0.3, 0.3–0.7 and 0.7–1.0 for excellent, medium and poor, respectively. The calculated values for the studied compounds were 0.692, 0.025 and 0.145, respectively, indicating that the probability for having a T_1/2_ of ≤ 3 h is low for **5a** and **5h**, whereas it is moderate for **3h**.

##### Toxicity

ADMETlab2.0 provides several descriptors to identify potential toxicities which could associate drugs and drug candidates. The most important properties will be discussed in the following section.

- The human ether-a-go-go-related gene (hERG) blockers

The hERG encodes a voltage-gated potassium channel, which is involved in the regulation of the exchange of cardiac action potential and resting potential. A blockade of hERG may result in long QT syndrome (LQTS), arrhythmia, and Torsade de Pointes (TdP), which may lead to palpitations, fainting, or even sudden death. Therefore, the molecules are classified for their inhibitory potential against hERG as category 0 (hERG-), which represents molecules exerting an IC_50_ of more than 10 μM or demonstrate less than 50% inhibition at 10 μM, and category 1 (hERG+), referring to molecules with an IC_50_ of less than 10 μM or more than 50% inhibition at 10 μM. The output value reflects the probability of being hERG^+^ blockers, within the range of 0–1. The compounds’ scores for being hERG^+^ blockers were predicted to be 0.01, 0.279 and 0.549, respectively, implying that **3h** and **5a** are weak hERG^+^ blockers, and can accordingly be regarded as non-cardiotoxic, whereas **5h** is expected to be a medium hERG^+^ blocker, indicating its hazard as a cardiotoxic agent.

- The human hepatotoxicity (H-HT)

The compounds were further screened for their hepatotoxicity. The ADMETLab 2.0 categorizes the molecules as category 0 if they are H-HT negative (−) or category 1 if they are H-HT positive (+), with the output value being the probability of being toxic, within the range of 0 to 1. The reported scores for the studied derivatives were 0.882, 0.851 and 0.228, respectively. Thus, only **5h** is not expected to cause hepatic injuries, whereas **3h** and **5a** might induce adverse hepatic effects.

- Drug-induced liver injury (DILI)

Over the past 50 years, DILI has become the most common safety issue causing drug withdrawal from the market. Therefore, the molecules are classified as category 0 if they are DILI negative (−), or category 1 if they are DILI positive (+) and the output value is the probability of being toxic, within the range of 0 to 1. As the predicted scores of the investigated compounds were 0.963, 0.923 and 0.971, thus all of them are expected to cause DILI.

- AMES Toxicity for mutagenicity

This assay is a widely used method to test chemicals for mutagenicity, which is positively correlated to carcinogenicity. According to the used platform, the compounds are identified as category 0 (AMES negative (−, nontoxic)) or category 1 (AMES positive (+, toxic)), with the output value reflecting the probability of being toxic, within the range of 0 to 1. The recorded AMES scores were 0.984, 0.504 and 0.986, respectively, indicating that **3h** and **5h** are strong mutagenic agents, while **5a** is a medium agent. The predicted scores were 0.606, 0.025 and 0.038, respectively; thus, **5a and 5h** are considered nontoxic, whereas **3h** is likely to cause toxicity.

- Rat oral acute toxicity (ROA)

The used web interface categorizes compounds according to the risk of causing acute toxicity in mammals upon administration into category 0 (low-toxicity, >500 mg/kg) and category 1 (high-toxicity, <500 mg/kg). The output value is the probability of being toxic, within the range of 0 to 1. The predicted scores were 0.606, 0.025 and 0.038, respectively, which indicates that **5a** and **5h** are expected to be nontoxic, whereas **3h** has a medium probability of being a toxic molecule.

- FDA Maximum Recommended Daily Dose (FDAMDD)

This descriptor estimates the toxic dose threshold of chemicals in humans. The compounds are categorized as category 1 (FDAMDD positive (+, toxic), ≤ 0.011 mmol/kg of body weight (bw)/day); or category 0 (FDAMDD negative (−, nontoxic), > 0.011 mmol/kg of bw/day). The output value is the probability of being toxic, within the range of 0 to 1. The computed scores were 0.881, 0.909 and 0.891, respectively, indicating that all of the compounds would be toxic substances.

- Carcinogenicity

This endpoint is assessed via the perdition of the TD_50_: the dose required to produce a toxic effect in 50% of the population. Molecules are designated as inactive (category 0; non-carcinogens) or active (category 1; carcinogens) according to their TD_50_ values. The output value is the probability of being toxic, within the range of 0 to 1. According to the computed values of 0.937, 0.425 and 0.866, respectively, only **5a** would be a moderately carcinogenic compound, whereas **3h** and **5h** are expected to be highly carcinogenic.

- Respiratory Toxicity

Herin, the compounds are labeled as category 1 (respiratory toxicants) or category 0 (non-respiratory toxicants), with the output value is the probability of being toxic, within the range of 0 to 1. As per the computed scores of 0.944, 0.647 and 0.864, respectively, only **5a** is accompanied by a medium risk of being a respiratory toxicant, whereas **3h** and **5h** would be strong respiratory toxicants.

- Eye irritation/eye corrosion (EI/EC)

The chemical may be classified as category 1 (irritant or corrosive) or category 0 (non-irritant/non-corrosive) with the output value is the probability of being toxic, within the range of 0 to 1. The computed values for the toxic probabilities were 0.03, 0.157 and 0.148 for eye irritation, and 0.003 for all of them as corrosives; thus, none of the compounds is likely to cause eye irritation or corrosion.

- Predicting the toxicology in the 21st century (Tox21) program

Furthermore, the toxicity of the compounds was evaluated according to the Tox21 dataset, which consists of two major panels: a nuclear receptor (NR) signaling panel and a stress response (SR) panel, which together comprise 12 in vitro assays.

*Nuclear receptor pathway toxicity*

Seven assays were used to evaluate the compounds for their capabilities of interacting with nuclear receptors (either as inhibitors or activators) that may cause the disruption of normal endocrine function, as well as interfere with metabolic homeostasis, reproduction, and developmental and behavioral functions.

1. Nuclear receptor–Androgen receptor (NR-AR)

The androgen receptor (AR) is a nuclear hormone receptor that plays a critical role in androgen-related diseases such as AR-dependent prostate cancer. Therefore, the used web tool categorizes the compounds either as category 1 (activator or agonist) or category 0 (inactivator). The output value is the probability of being AR agonists, within the range of 0 to 1. The predicted probability values were 0.134, 0.338 and 0.607, respectively. Accordingly, **3h** is not a possible agonist, whereas **5a** and **5h** have medium probabilities of being AR agonists.

2. Nuclear receptor–Androgen receptor ligand binding domain (NR-AR-LBD)

This receptor is also implicated in androgen-related diseases; thus, in this bioassay, chemicals which are capable of binding to the AR-LBD belong to category 1 (active) and the output value is the probability of being active, within the range of 0 to 1. The predicted probability values were 0.554, 0.025 and 0.225, respectively, indicating that **3h** and **5h** may bind with this receptor, whereas **5a** may not bind with it.

3. Nuclear receptor–Aryl hydrocarbon Receptor (NR-AhR)

The AhR is pivotal for adaptive responses against environmental pollutants such as aromatic hydrocarbons and environmental changes through the induction of phase I and II enzymes; in addition, it interacts with other nuclear receptor signaling pathways. The molecules may belong to category 0, or category 1, wherein molecules labeled as category 1 act to activate the aryl hydrocarbon receptor signaling pathway, with the output value is the probability of being an activator, within the range of 0 to 1. The computed values were 0.885, 0.511 and 0.846, respectively; thus, **3h** and **5h** have a high probability of being AhR activators, whereas **5a** can be considered as a moderate activator.

4. NR-Aromatase

Aromatase catalyzes the conversion of androgen to estrogen and plays a crucial role in maintaining the balance between the two hormones in many of the endocrine disrupting chemical (EDC)-sensitive organs. The compounds may be labelled as category 1 (active) or category 0 (inactive). The label of category 1 designates aromatase inhibitors, with the output probability value being within the range of 0 to 1. The predicted values were 0.885, 0.902 and 0.924, respectively. Thus, all the compounds have a high probability of being aromatase inhibitors and could cause an imbalance between androgen and estrogen.

5. Nuclear receptor–Estrogen receptor (NR-ER)

Certain chemicals may interact with steroid hormone receptors like the ER, thus causing the disruption of normal endocrine function; these chemicals are called endocrine disrupting chemicals (EDCs). Therefore, chemicals and drugs are assessed for their effect on the ER signaling pathway as being either category 1 (active) or category 0 (inactive), with the output value indicating the probability of being active within the range of 0 to 1. The computed values were 0.552, 0.474 and 0.519, implying that the three compounds have medium probabilities for interfering with the ER signaling pathway.

6. Nuclear receptor–Estrogen receptor α ligand binding domain (NR-ER-LBD)

Likewise, compounds may be labeled as active or inactive towards the NR-ER-LBD. The output value is the probability of being active, within the range of 0 to 1. The predicted values were 0.491, 0.102 and 0.362; as a consequence, the probability that **5a** interferes with the ER-LBD pathway is negligible, whereas **3h** and **5h** have medium probabilities for interference with this pathway.

7. Nuclear receptor–peroxisome proliferator-activated receptors gamma (NR-PPARg)

Furthermore, the compounds may be categorized as active (category 1) or inactive (category 0) for their interference with the PPARg receptor (also known as the glitazone reverse insulin resistance receptor*)*, which is involved in the regulation of the glucose and lipid metabolism. The output value is the probability of being active within the range of 0 to 1. The estimated values were 0.446, 0.933 and 0.807, respectively; thus, **5a** and **5h** have medium probabilities, whereas **3h** has a moderate probability for interference with PPARg.

*Stress response (SR) panel*

Toxicity may also lead to cellular oxidative stress, which in turn leads to apoptosis. Thus, the compounds were also screened with regard to five stress response assays, as follows:

1. The antioxidant response element signaling pathway (SR-ARE)

The ARE directly reacts to cellular stress; therefore, the chemicals are labelled as category 1 (active) or category 0 (inactive), with the output value indicating the probability of being active within the range of 0 to 1. According to the computed values of 0.816, 0.912 and 0.912, all compounds have a high probability for being active in ARE signaling.

2. ATPase family AAA domain-containing protein 5 (SR-ATAD5)

According to this assay, compounds which are expected to cause DNA damage, thus enhancing the levels of genome instability gene 1 (ELG1; human ATAD5) protein, are labeled as category 1 (active), while those would not cause DNA damage are labeled as category 0 (inactive), with the output value is the probability of being active within the range of 0 to 1. The predicted values were 0.749, 0.012 and 0.542, respectively, indicating the safety of **5a** with regard to this indicator, whereas **3h** and **5h** have medium probabilities for being inducers to genomic instability.

3. Heat shock factor response element (SR-HSE)

This pathway is activated in response to stresses (such as elevated temperature, oxidant accumulation and abrupt alterations in osmolarity) that damage cellular proteins. Therefore, the compounds were evaluated as being activators (category 1) or non-activators (category 0) for the HSE, with the output value indicating the probability of being active within the range of 0 to 1. The computed values were 0.593, 0.314 and 0.091, respectively, implying that **3h** has a medium probability of being a stressor, whereas **5a** and **5h** have negligible probabilities as stressors; accordingly, they are safer.

4. Mitochondrial membrane potential (SR-MMP, ΔΨm)

Compounds may cause mitochondrial toxicity via different mechanisms of action, one of them being influencing the MMP. Compounds that disrupt mitochondrial function may induce adverse effects such as liver injury. Therefore, the compounds were evaluated as being active (category 1) or inactive (category 0) with regard to disrupting the MMP, with the output value indicating the probability of being active within the range of 0 to 1. The predicted values were 0.875, 0.898 and 0.926; thus, all of the compounds are expected to cause MMP.

5. p53, a tumor suppressor protein (SR- p53)

This pathway is a well-known cancer pathway, which is activated following DNA damage or other cellular stressors. Thus, compounds which do not activate p53 are labelled (category 0), while those activate it are labeled category 1 and they are considered as toxic molecules. The output value is the probability of being an activator within the range of 0 to 1. The estimated values were 0.893, 0.909 and 0.916, respectively; accordingly, all of them are potential toxic molecules via activating the p53 pathway.

- The prediction of the existence or absence of toxicophores and compliance with toxicity rules:
  1. The number of toxic substructures (toxicophores) present in the studied compounds were 5, 1 and 3, respectively;
  2. Based on the genotoxic carcinogenicity rule, the molecules possessed 5, 0 and 5 substructures, respectively, which would cause carcinogenicity or mutagenicity through genotoxic mechanisms;
  3. Based on the non-genotoxic carcinogenicity rule, the molecules possessed 0, 1 and 1 substructures, respectively, which would cause carcinogenicity through nongenotoxic mechanisms;
  4. Based on the skin sensitization rule, the molecules possessed 1, 1 and 2 substructures, respectively, which would cause skin irritation;
  5. Based on the non-biodegradable rule, the molecules possessed 2, 2 and 3 substructures, respectively, which would make them non-biodegradable;
  6. Based on the SureChEMBL rule, the molecules did not have any structural alerts to have a MedChem unfriendly status.

- Predictions of environmental toxicity
  1. Bioconcentration Factor (BCF)
    The BCF is used to reflect on secondary poisoning potential and evaluate risks to human health via the food chain. The unit of BCF is log10(L/kg) and it is calculated based on the ratio of a chemical concentration in the organism as a result of absorption via the respiratory and dermal surfaces to that in water at a steady state. Substances are considered highly bioaccumulative (should be severely restricted) with a BCF value of ≥ 3.7, accumulative with BCF ranging between 3 and 3.3, and non-accumulative having BCF values of < 3, according to the US Environmental Protection Agency under the Toxic Substances Control Act [104]. For the studied compounds the computed values were 0.741, 1.7 and 2.212; thus, all of them would be non-bioaccumulative, and so they are considered to be non-ecotoxic.
  2. The 50% growth inhibitory concentration (IGC_50_) for *Tetrahymena pyriformis*
    This value indicates aquatic toxicity by estimating the concentration of a chemical in water in mg/L that causes 50% growth inhibition to *Tetrahymena pyriformis* after 48 h. The predicted values in units of [−log10[(mg/L)/(1000 × MW)] were 4.771, 5.339 and 5.222, respectively.
  3. The median lethal concentration values against flathead minnow (LC_50_FM)
    This value refers to the concentration of a molecule in water in mg/L that causes 50% of fathead minnow to die after 96 h, expressed in [−log10[(mg/L)/(1000 × MW)]. The computed values were 5.083, 6.709 and 6.738 unit.
  4. The median lethal concentration values against Daphnia magna (LC_50_DM)
    This value is defined as the concentration of a compound in water in mg/L which causes death to 50% of a population of Daphnia magna after 48 h. The recorded values for the compounds were 5.959, 6.99 and 6.865 [−log10[(mg/L)/(1000 × MW], respectively.

## 3. Materials and Methods

### 3.1. Chemistry

#### 3.1.1. General

All reagents were supplied commercially and they were used without further purification. The melting points (°C) were determined using an electronic melting point apparatus (Electrothermal, Essex, UK) and they were uncorrected. IR spectra were recorded on the Perkin Elmer FT-IR system, spectrum BX, as wave numbers (cm^−1^) using KBr discs. The NMR analyses were performed in deuterated dimethyl sulfoxide (DMSO-*d*_6_) and they were recorded on different Spectrometers (Tokyo, Japan) including, an Eclipse 300 FT NMR Spectrometer at 300 MHz and on a JNM-Ecx500II FT NMR system spectrometer at 500 MHz for the ^1^HNMR analysis, whereas the ^13^CNMR on the Agilent NMR-600, was working at 150 MHz. Chemical shifts (*δ*) were expressed in ppm relative to tetramethylsilane (TMS) as the internal standard; the coupling constant values (*J*) were expressed in Hz and the splitting patterns of ^1^HNMR were designated as s (singlet), d (doublet), t (triplet), dd (double-doublet), app. (apparent), m (multiplet) and q (quartet). Mass spectra were carried out on a direct probe controller inlet part of a single quadrupole mass analyzer in the Thermo Scientific GCMS model (ISO Lt) using Thermo X-Calibur Software 3.0.

#### 3.1.2. General Procedures for the Synthesis of 6-substituted or 6,7-disubstituted-3-aryl-2-thioxo-2,3-dihydro-1*H*-quinazolin-4-one **3a**–**h**

To a suspension of anthranilic acid derivative **1a**-**c** (0.054 mol) namely; 5-chloro anthranilic acid, 5-bromo anthranilic or 4,5-dimethoxy anthranilic acid in glacial acetic acid (50 mL), the appropriate phenyl isothiocyanate derivative **2a**-**c** (0.054 mol) namely; 3-(trifluoromethyl)phenyl isothiocyanate, 4-(trifluoromethyl)phenyl isothiocyanate or 4-nitrophenyl isothiocyanate was added. After refluxing the resulting reaction mixture for 10 h [70], the formed solid in each case was collected via filtration, washed with an excess of water, air-dried and recrystallized from ethanol to give the pure compound. The obtained products would exist in two tautomeric forms, **3a**-**h** and **3’a**-**h** (6,7-disubstituted-2-mercapto-3-aryl-3*H*-quinazolin-4-one derivatives). The IR as well as the ^1^H and ^13^CNMR data proved the predominance of the **3a**-**h** tautomeric forms.

*6-Chloro-2-thioxo-3-(3-trifluoromethyl-phenyl)-2,3-dihydro-1H-quinazolin-4-one* (**3a**), off- white, yield (80%), m.p. 275–276 °C; ν_max_ (KBr)/cm^−1^ 3245 (NH), 3108 and 3038 (CH- aromatic), 1888, 1838, 1664 (C=O), 1619; 1523 and 1484 (C=C), 1450, 1389, 1332, 1269, 1212, 1174, 1129, 1069, 1003, 919, 864, 833, 800, 761, 724, 694, 663, 615, 563, 540, 474; ^1^H-NMR (500 MH_Z_; DMSO-*d*_6_) *δ*_H_: 13.19 (1H, s, NH), 7.84 (1H, d, *J* = 1.5 Hz, CH-aromatic), 7.81 (1H, app. dd, *J* = 8.5, 1.5 Hz, CH-aromatic), 7.78–7.75 (2H, m, 2 × CH- aromatic), 7.70 (1H, t, *J* = 7.5 Hz, CH-aromatic), 7.58 (1H, d, *J* = 7.5 Hz, CH-aromatic), 7.42 (1H, d, *J* = 8.5 Hz, CH-aromatic); ^13^C-NMR (150 MH_Z_; DMSO-*d*_6_) *δ*_C_: 176.24 (C=S), 159.39 (C=O), 140.31, 138.86, 136.00, 133.93, 130.61, 130.31, 130.09, 128.70, 126.67, 125.57, 125.27, 123.44, 118.45, 118.24 (7 × CH-aromatic, 5 × C_q_-aromatic, and CF_3_); MS (EI) *m/z* (%) [M^+^ + 2, ^37^Cl] 360.11 (2.11), [M^+^ + 1, ^37^Cl] 359.05 (5.22), [M^+^, ^37^Cl] 358.08 (16.11), [M^+^ +1, ^35^Cl] 357.07 (43.94), [M^+^, ^35^Cl] 356.10 (60.87) for C_15_H_8_ClF_3_N_2_OS, 355.04 (100.00), 337.42 (3.36), 322.97 (8.39), 287.11 (6.40), 287.03 (7.52), 259.98 (4.42), 195.89 (1.34), 180.93 (2.94), 168.23 (2.13), 153.01 (4.97), 145.28 (4.29), 132.31 (4.01), 125.20 (14.72), 107.94 (5.36), 90.19 (11.34), 75.04 (16.95), 74.10 (17.68), 69.03 (59.01), 50.24 (5.78), 43.14 (5.14).

*6-Bromo-2-thioxo-3-(3-trifluoromethyl-phenyl)-2,3-dihydro-1H-quinazolin-4-one* (**3b**), off- white powder, yield (72%), m.p. 282–283 °C; ν_max_ (KBr)/cm^−1^ 3164 (NH), 3094 (CH-aromatic), 2996, 2935, 1726 (C=O), 1616; 1532 and 1475 (C=C), 1452, 1432, 1389, 1334, 1264, 1211, 1170, 1115, 1071, 998, 910, 855, 810, 757, 693, 612, 549, 436; ^1^H-NMR (500 MH_Z_; DMSO-*d*_6_) *δ*_H_: 13.18 (1H, s, NH), 7.97 (1H, d, *J* = 2.0 Hz, CH-aromatic), 7.92 (1H, app. dd, *J* = 8.5, 2.0 Hz, CH-aromatic), 7.75–7.73 (2H, m, 2 × CH- aromatic), 7.68 (1H, t, *J* = 7.5 Hz, CH-aromatic), 7.58 (1H, d, *J* = 7.5 Hz, CH-aromatic), 7.35 (1H, d, *J* = 8.5 Hz, CH-aromatic); ^13^C-NMR (150 MH_Z_; DMSO-*d*_6_) *δ*_C_: 176.28 (C=S), 159.27 (C=O), 140.30, 139.17, 138.69, 133.93, 130.63, 130.30, 130.09, 129.68, 126.66, 125.58, 125.26, 123.46, 118.59, 116.41 (7 × CH-aromatic, 5 × C_q_-aromatic, and CF_3_); MS (EI) *m/z* (%) [M^+^ + 1, ^81^Br] 403.06 (4.16), [M^+^, ^81^Br] 402.04 (11.42), [M^+^ + 1, ^79^Br] 401.06 (15.01), [M^+^, ^79^Br] 400.05 (16.06) for C_15_H_8_BrF_3_N_2_OS, 197.12 (1.48), 333.14 (1.50), 169.05 (2.24), 145.10 (4.86), 133.13 (2.55), 125.19 (4.29), 106.23 (1.66), 90.15 (13.78), 75.04 (28.55), 74.11 (14.98), 69.08 (100.00), 63.12 (43.07), 50.07 (6.06), 43.17 (2.07).

*6,7-Dimethoxy-2-thioxo-3-(3-trifluoromethyl-phenyl)-2,3-dihydro-1H-quinazolin-4-one* (**3c**), off-white solid, yield (68%), m.p. 304–305 °C; ν_max_ (KBr)/cm^−1^ 3178 (NH), 3115 and 3035 (CH-aromatic), 2973 (CH-aliphatic), 2279, 1698 (C=O), 1626; 1548 and 1512 (C=C), 1459, 1429, 1399, 1332, 1290, 1248, 1203, 1174, 1113, 1032, 992, 977, 819, 761, 698, 654, 597, 556, 456; ^1^H-NMR (500 MH_Z_; DMSO-*d*_6_) *δ*_H_: 12.91 (1H, s, NH), 7.73 (1H, d, *J* = 7.5 Hz, CH-aromatic), 7.69 (1H, s, CH-aromatic), 7.66 (1H, app t, *J* = 8.0 Hz, CH- aromatic), 7.56 (1H, d, *J* = 8.5 Hz, CH-aromatic), 7.24 (1H, s, CH-aromatic), 6.95 (1H, s, CH-aromatic), 3.83 (3H, s, OCH_3_), 3.77 (3H, s, OCH_3_); ^13^C-NMR (125 MH_Z_; DMSO-*d*_6_) *δ*_C_: 174.66 (C=S), 159.34 (C=O), 155.41, 146.58, 140.23, 135.63, 133.62, 130.03, 129.80, 126.38, 125.03, 124.89, 122.87, 108.66, 106.94, 97.98 (6 × CH-aromatic, 6 × C_q_-aromatic, and CF_3_), 56.01 (OCH_3_), 55.84 (OCH_3_); MS (EI) *m/z* (%) [M^+^ + 2] 384.11 (4.47), [M^+^ + 1] 383.17 (27.26), [M ^+^ ] 382.16 (100.00) for C_17_H_13_F_3_N_2_O_3_S, 381.18 (49.46), 367.08 (21.32), 349.00 (8.35), 336.13 (17.89), 321.03 (1.88%), 308.23 (5.29), 280.19 (1.52), 267.24 (1.27), 145.23 (1.91), 69.15 (19.97).

*6-Chloro-2-thioxo-3-(4-trifluoromethyl-phenyl)-2,3-dihydro-1H-quinazolin-4-one* (**3d**), white powder, yield (84%), m.p. 375–377 °C; ν_max_ (KBr)/cm^−1^ 3164 (NH), 3095 and 3001 (CH-aromatic), 2341, 1925, 1840, 1800, 1702 (C=O), 1618; 1534 and 1482 (C=C), 1435, 1395, 1330, 1262, 1214, 1162, 1111, 1066, 1021, 990, 956, 913, 822, 760, 715, 680, 650, 596, 536, 475, 420; ^1^H-NMR (500 MH_Z_; DMSO-*d*_6_) *δ*_H_: 13.20 (1H, s, NH), 7.85–7.79 (4H, m, 4 × CH-aromatic), 7.52 (2H, d, *J* = 8.5 Hz, 2 × CH-aromatic), 7.42 (1H, d, *J* = 9.0 Hz, CH-aromatic); ^13^C-NMR (150 MH_Z_; DMSO-*d*_6_) *δ*_C_: 176.00 (C=S), 159.25 (C=O), 143.20, 138.88, 136.00, 130.64, 129.26, 128.70, 126.56, 125.41, 123.59, 118.45, 118.14 (7 × CH-aromatic, 5 × C_q_-aromatic, and CF_3_); MS (EI) *m/z* (%) [M^+^ + 1, ^37^Cl] 358.91 (1.63), [M^+^, ^37^Cl] 358.03 (28.37), [M^+^ + 1, ^35^Cl] 357.01 (18.30), [M^+^, ^35^Cl] 356.09 (64.18) for C_15_H_8_ClF_3_N_2_OS, 355.09 (100.00), 337.03 (6.02), 322.95 (10.78), 297.27 (7.64), 285.99 (9.01), 277.45 (4.42), 261.29 (6.04), 236.61 (2.79), 228.19 (3.05), 195.87 (5.98), 179.29 (3.94), 153.18 (11.88), 133.17 (5.08), 126.15 (25.96), 114.32 (4.67), 90.21 (15.36), 75.09 (41.28), 69.16 (56.28%), 50.16 (14.81), 43.28 (6.46).

*6-Bromo-2-thioxo-3-(4-trifluoromethyl-phenyl)-2,3-dihydro-1H-quinazolin-4-one* (**3e**), white powder, yield (67%), m.p. 359–360 °C; ν_max_ (KBr)/cm^−1^ 3161 (NH), 3089 (CH-aromatic), 2996 2938, 2373, 2342, 1921, 1850, 1798, 1705 (C=O), 1616; 1533 and 1478 (C=C), 1432, 1393, 1329, 1261, 1213, 1163, 1067, 1022, 989, 957, 927, 901, 821, 761, 710, 651, 595, 531, 440; ^1^H-NMR (500 MH_Z_; DMSO-*d*_6_) *δ*_H_: 13.19 (1H, s, NH), 7.97 (1H, d, *J* = 1.5 Hz, CH-aromatic), 7.92 (1H, app. dd, *J* = 8.5, 2.0 Hz, CH-aromatic), 7.82 (2H, d, *J* = 9.0 Hz, CH-aromatic), 7.51 (2H, app. d, *J* = 8.5 Hz, 2 × CH-aromatic), 7.35 (1H, app. d, *J* = 8.5 Hz, CH-aromatic); ^13^C-NMR (150 MH_Z_; DMSO-*d*_6_) *δ*_C_: 176.04 (C=S), 159.16 (C=O), 143.24, 139.25, 138.69, 130.63, 129.64, 126.56, 118.65, 118.54, 116.38 (7 × CH-aromatic, 5 × C_q_-aromatic, and CF_3_); MS (EI) *m/z* (%) [M^+^ + 1, ^81^Br] 402.94 (10.26), [M^+^, ^81^Br] 401.98 (29.26), [M^+^ + 1, ^79^Br] 401.09 (39.11), [M^+^, ^79^Br] 400.04 (39.62) for C_15_H_8_BrF_3_N_2_OS, 399.06 (22.17), 381.07 (3.47), 369.02 (3.38), 330.94 (36.62), 320.92 (3.04), 292.29 (2.57), 239.87 (1.76), 182.97 (1.57), 145.09 (2.87), 89.23 (2.30), 75.01 (5.70), 69.10 (100.00%), 50.2 (1.54).

*6-Chloro-3-(4-nitro-phenyl)-2-thioxo-2,3-dihydro-1H-quinazolin-4-one* (**3f**), yellow powder, yield (68%), m.p. 343–344 °C; ν_max_ (KBr)/cm^−1^ 3157 (NH), 3088, (CH-aromatic), 2995, 1698 (C=O), 1616; 1519 and 1475 (C=C), 1432, 1392, 1348, 1256, 1211, 1085, 1017, 990, 912, 823, 760, 694, 647, 587, 535, 475, 426; ^1^H-NMR (500 MH_Z_; DMSO-*d*_6_) *δ*_H_: 13.24 (1H, s, NH), 8.34–8.27 (2H, m, 2 × CH-aromatic), 7.85 (1H, d, *J* = 3.0 Hz, CH-aromatic), 7.81 (1H, app. dd, *J* = 8.5, 3.0 Hz, CH-aromatic), 7.62–7.57 (2H, m, 2 × CH of 4-NO_2_-C_6_H_4_), 7.42 (1H, d, *J* = 9.0 Hz, CH-aromatic); ^13^C-NMR (75 MH_Z_; DMSO-*d*_6_) *δ*_C_: 175.63 (C=S), 159.04 (C=O), 147.38, 145.24, 138.69, 135.86, 131.07, 128.64, 126.46, 124.56, 118.28, 117.94 (7 × CH-aromatic and 5 × C_q_-aromatic); MS (EI) *m/z* (%) [M^+^, ^37^Cl] 335.05 (26.91), [M^+^ + 1, ^35^Cl] 334.10 (33.29), [M^+^, ^35^Cl] 333.06 (57.07) for C_14_H_8_ClN_3_O_3_S, 332.07 (44.01), 302.82 (3.742), 285.97 (24.99), 257.94 (1.91), 231.34 (2.62), 215.77 (2.31), 183.85 (5.07), 153.03 (3.33), 133.00 (5.69), 124.26 (20.51), 110.11 (5.45), 90.08 (18.18), 75.22 (55.60), 63.18 (100.00), (50.19 (22.63), 46.12 (15.38).

*6-Bromo-3-(4-nitro-phenyl)-2-thioxo-2,3-dihydro-1H-quinazolin-4-one* (**3g**), yellow powder, yield (65%), m.p. 340–341 °C; ν_max_ (KBr)/cm^−1^ 3155 (NH), 3084 (CH-aromatic), 2992, 1698 (C=O), 1614; 1517 and 1471 (C=C), 1429, 1390, 1346, 1256, 1210, 1071, 989, 906, 852, 821, 760, 690, 646, 587, 526, 444, 421; ^1^H-NMR (500 MH_Z_; DMSO-*d*_6_) *δ*_H_: 13.23 (1H, s, NH), 8.33–8.28 (2H, m, 2 × CH of 4-NO_2_-C_6_H_4_), 7.97 (1H, d, *J* = 2.0 Hz, CH^5^-quinazolin-4(3*H*)-one), 7.93 (1H, dd, *J* = 8.5, 2.0 Hz, CH^7^-quinazolin-4(3*H*)-one), 7.62–7.56 (2H, m, 2 × CH of 4-NO_2_-C_6_H_4_), 7.35 (1H, d, *J* = 8.5 Hz, CH^8^-quinazolin-4(3*H*)-one); ^13^C-NMR (75 MH_Z_; DMSO-*d*_6_) *δ*_C_: 175.62 (C=S), 158.86 (C=O), 147.38, 145.17, 139.01, 138.54, 131.07, 129.46, 124.56, 118.43, 118.28, 116.30 (7 × CH-aromatic and 5 × C_q_-aromatic); MS (EI) *m/z* (%) [M^+^ + 2, ^81^Br] 381.07 (2.28), [M^+^ + 1, ^81^Br] 380.11 (8.00), [M^+^, ^81^Br] 379.05 (35.08), [M^+^ + 1, ^79^Br] 378.01 (47.20), [M^+^, ^79^Br] 377.02 (39.84) for C_14_H_8_BrN_3_O_3_S, 376.01 (39.79), 361.40 (1.64), 344.03 (2.49), 332.03 (16.15), 298.12 (4.51), 271.96 (2.41), 239.99 (2.86), 224.17 (3.44), 197.08 (10.80), 168.09 (5.36), 133.10 (11.22), 116.23 (6.34), 108.09 (5.97), 90.12 (43.77), 75.18 (48.59), 50.08 (13.32), 46.07 (6.82).

*6,7-Dimethoxy-3-(4-nitro-phenyl)-2-thioxo-2,3-dihydro-1H-quinazolin-4-one* (**3h**), yellow powder, yield (54%), m.p. 328–329 °C; ν_max_ (KBr)/cm^−1^ 3177 (NH), 3113 and 3033 (CH-aromatic), 2972 (CH-aliphatic), 1696 (C=O), 1625; 1550 and 1514 (C=C), 1428, 1400, 1348, 1292, 1247, 1200, 1156, 1064, 1024, 988, 855, 818, 756, 708, 684, 647, 607, 581, 498, 426; ^1^H-NMR (500 MHz; DMSO-*d*_6_) *δ*_H_: 12.96 (1H, s, NH), 8.32–8.24 (2H, m, 2 × CH-aromatic), 7.62–7.54 (2H, m, 2 × CH-aromatic), 7.24 (1H, s, CH-aromatic), 6.95 (1H, s, CH-aromatic), 3.83 (3H, s, OCH_3_), 3.77 (3H, s, OCH_3_); ^13^C-NMR (75 MH_Z_; DMSO-*d*_6_) *δ*_C_: 174.38 (C=S), 159.33 (C=O), 155.69, 147.24, 146.82, 145.59, 135.86, 131.17, 124.40, 108.77, 107.08, 98.23 (6 × CH-aromatic and 6 × C_q_-aromatic), 56.23 (OCH_3_), 56.06 (OCH_3_); MS (EI) *m/z* (%) [M^+^ + 2] 361.20 (4.32), [M^+^ + 1] 360.21 (17.90), [M^+^] 359.19 (100.00) for C_16_H_13_N_3_O_5_S, 358.20 (55.20), 343.20 (2.79), 326.17 (4.69), 312.18 (4.66), 297.15 (3.91), 269.11 (5.30), 255.12 (6.26), 222.13 (3.54), 205.15 (3.67), 179.20 (22.25), 164.14 (50.57), 150.15 (21.00), 136.12 (55.73), 120.11 (12.10), 108.13 (29.19), 90.13 (21.26), 76.11 (20.09), 63.15 (35.96), 50.15 (23.96), 44.12 (8.94).

#### 3.1.3. Synthesis of 2-(benzylsulfanyl)-3-aryl-3*H*-quinazolin-4-one Derivatives **5a**–**h**

##### General Procedures

Potassium carbonate (3 equiv., 0.0054 mol, 0.74 g) was added [63] to an equimolar mixture (0.0018 mol) of 6-substituted or 6,7-disubstituted-3-aryl-2-thioxo-2,3-dihydro-1*H*-quinazolin-4-one **3a**–**h** and aryl halide derivative Ar-CH_2_-X **4a**–**d**, namely (in order) 2-fluoro benzyl bromide, 4-chlorobenzyl chloride, 3-nitro benzyl bromide or 2-bromo-5-methoxy benzyl bromide in dry acetone (20 mL). After refluxing the reaction mixture for 6 h, it was filtered while hot to isolate potassium carbonate and the filtrate was concentrated under reduced pressure to give the crude solid product. The obtained solid in each case was washed with water, air-dried and recrystallized from the appropriate solvent to give compounds **5a**–**h** in pure forms.

*2-(2-Fluoro-benzylsulfanyl)-6-chloro-3-(3-trifluoromethyl-phenyl)-3H-quinazolin-4-one* (**5a**), white powder (EtOH/CHCl_3_), yield (80%), m.p. 148–149 °C; ν_max_ (KBr)/cm^−1^ 3084 (CH-aromatic), 2989 and 2929 (CH-aliphatic), 1923, 1797, 1699 (C=O), 1547 (C=N), 1466 and 1402 (C=C), 1333, 1304, 1282, 1248, 1199, 1170, 1120, 1095, 1071, 976, 895, 873, 833, 807, 763, 696, 612, 540, 562, 420; ^1^H-NMR (300 MHz; DMSO-*d*_6_) *δ*_H_: 8.12-7.50 (8H, m, 8 × CH-aromatic), 7.38–7.04 (3H, m, 3 × CH-aromatic), 4.46 (2H, s, S-CH_2_); ^13^C-NMR (75 MHz; DMSO-*d*_6_) *δ*_C_: 162.27 (C=O), 159.97 (C=N), 159.02 (C_q_-F), 156.83 145.99, 136.50, 135.17, 133.97, 131.94, 1130.98, 130.30, 129.90, 129.80, 128.43, 127.08, 126.79, 125.59, 124.56, 121.13, 115.56, 115.28 (11 × CH-aromatic, 6 × C_q_-aromatic, and CF_3_), 29.79 (CH_2_); MS (EI) *m/z* (%) [M^+^, ^37^Cl] 467.21 (2.86), [M^+^ + 1, ^35^Cl] 466.06 (3.45), [M^+^, ^35^Cl] 465.32 (1.41) for C_22_H_13_ClF_4_N_2_OS, 464.10 (19.39), 430.49 (3.42), 411.50 (6.55), 377.95 (3.80), 355.03 (9.71), 339.47 (11.09), 320.17 (52.77), 308.94 (13.30), 296.96 (16.64), 287.14 (47.96), 269.09 (10.39), 253.19 (41.03), 244.25 (6.72), 233.28 (5.24), 225.90 (1.93), 191.88 (3.35), 168.39 (6.01), 145.14 (3.60), 132.93 (7.28), 124.01 (8.68), 109.12 (100.00), 83.14 (16.33), 76.04 (7.36), 69.36 (1.67), 63.56 (14.33), 57.31 (4.36), 44.95 (7.26).

*2-(4-Chloro-benzylsulfanyl)-6-bromo-3-(3-trifluoromethyl-phenyl)-3H-quinazolin-4-one* (**5b**), off-white solid (EtOH/CHCl_3_), yield (80%), m.p. 193–194 °C; ν_max_ (KBr)/cm^−1^ 3056 (CH-aromatic), 2935 (CH-aliphatic), 2298, 1907, 1690 (C=O), 1541 (C=N), 1490 and 1463 (C=C), 1331, 1289, 1249, 1176, 1125, 1092, 1067, 1013, 977, 910, 836, 809, 784, 744, 696, 610, 538, 507, 440; ^1^H-NMR (300 MHz; DMSO-*d*_6_) *δ*_H_: 8.14 (1H, d, *J* = 2.4 Hz, CH-aromatic), 8.08–7.807.68 (5H, m, 5 × CH-aromatic), 7.68 (1H, d, *J* = 8.7 Hz, CH-aromatic), 7.59 (2H, d, *J* = 8.4, 2 × CH-aromatic), 7.35 (2H, d, *J* = 8.4 Hz, 2 × CH-aromatic), 4.43 (2H, AB q, *J* = 13.5 Hz, S-CH_2_); ^13^C-NMR (150 MHz; CDCl_3_) *δ*_C_: 160.35 (C=O), 156.60 (C=N), 146.35, 138.11, 135.87, 134.55, 133.57, 132.68, 130.64, 130.42, 129.73, 128.75, 128.05, 127.08, 126.37, 121.08, 119.44 (11 × CH-aromatic, 7 × C_q_-aromatic, and CF_3_), 36.37 (CH_2_); MS (EI) *m/z* (%) [M^+^ + 2, ^81^Br and ^37^Cl] 528.05 (24.98), [M^+^ + 1, ^81^Br and ^37^Cl] 527.05 (31.94), [M ^+^, ^81^Br and ^37^Cl] 526.03 (100.00), [M^+^ + 1, ^79^Br and ^35^Cl] 525.07 (24.52), [M^+^, ^79^Br and ^35^Cl] 524.05 (82.52) for C_22_H_13_BrClF_3_N_2_OS, 509.04 (2.46), 507.07 (5.47), 493.07 (44.88), 491.09 (35.03), 458.12 (3.28), 456.09 (3.77), 320.12 (4.10), 268.91 (1.50).

*2-(3-Nitrobenzylsulfanyl)-6,7-dimethoxy-3(3-trifluoromethyl-phenyl)-3H-quinazolin-4-one* (**5c**), off-white powder (EtOH/CHCl_3_), yield (51%), m.p. 245–246 °C; ν_max_ (KBr)/cm^−1^ 3067 (CH-aromatic), 2971 and 2938 (CH-aliphatic), 2831, 1918, 1849, 1681 (C=O), 1613 (C=N), 1580; 1535 and 1499 (C=C), 1454, 1388, 1352, 1330, 1273, 1240, 1171, 1129, 1072, 1027, 1002, 981, 922, 868, 846, 808, 758, 695, 675, 588, 528; ^1^H-NMR (300 MHz; DMSO-*d*_6_) *δ*_H_: 8.52 (1H, app. dd, *J* = 6.3, 2.1 Hz, CH-aromatic), 8.09 (1H, app. dd, *J* = 8.4, 2.4 Hz, CH-aromatic), 7.98 (1H, s, CH-aromatic), 7.93 (2H, d, *J* = 7.8 Hz, 2 × CH-aromatic), 7.83-7.77 (2H, m, 2 × CH-aromatic), 7.60 (1H, t, *J* = 7.8 Hz, CH-aromatic), 7.37 (1H, s, CH-aromatic), 7.24 (1H, s, CH-aromatic), 4.53 (2H, AB q, *J* = 13.5 Hz, S-CH_2_); 3.99 (3H, s, OCH_3_), 3.85 (3H, s, OCH_3_); ^13^C-NMR (150 MHz; DMSO-*d*_6_) *δ*_C_: 160.55 (C=O), 155.47 (C=N), 154.15 (2 × C_q_-O), 148.48, 147.74, 143.81, 140.41, 137.27, 136.68, 134.42, 131.20, 130.26, 127.15, 125.21, 122.55, 112.68, 107.45, 106.06 (10 × CH-aromatic, 6 × C_q_-aromatic, and CF_3_), 56.43 (OCH_3_), 56.19 (OCH_3_), 35.12 (CH_2_); MS (EI) *m/z* (%) [M^+^ + 2] 519.10 (6.53), [M^+^ + 1] 518.19 (29.95), [M^+^] 517.18 (100.00) for C_24_H_18_F_3_N_3_O_5_S 501.23 (3.23), 500.21 (9.57), 484.26 (3.90), 381.12 (4.91), 395.07 (1.59), 372.13 (1.22), 350.20 (2.09), 339.51 (1.92), 323.13 (2.02), 294.26 (2.49), 179.09 (2.06), 164.08 (1.62), 150.16 (1.78), 145.06 (1.30), 136.22 (8.05), 121.29 (1.38), 90.21 (15.71), 89.20 (15.26), 77.05 (2.71), 63.25 (4.11).

*2-(4-Chloro-benzylsulfanyl)-6-chloro-3-(4-trifluoromethyl-phenyl)-3H-quinazolin-4-one* (**5d**), off-white solid (EtOH), yield (80%), m.p. 259–260 °C; ν_max_ (KBr)/cm^−1^ 3091 and 3063 (CH-aromatic), 2926 and 2856 (CH-aliphatic), 2369, 2340, 1898, 1850, 1776, 1695 (C=O), 1571 (C=N), 1549; 1464 and 1413 (C=C), 1328, 1269, 1203, 1162, 1114, 1066, 1019, 968, 930, 826, 784, 734, 712, 678, 642, 598, 543, 499, 466, 424; ^1^H-NMR (300 MH_Z_; CDCl_3_) *δ*_H_: 7.73 (1H, app. s, CH-aromatic), 7.34 (2H, d, *J* = 8.1 Hz, 2 × CH-aromatic), 7.25 (1H, dd, *J* = 8.7, 2.4 Hz, CH-aromatic), 7.15 (1H, d, *J*= 8.7 Hz, CH-aromatic), 6.97 (2H, d, *J* = 8.1 Hz, 2 × CH-aromatic), 6.88–6.77 (4H, m, 4 × CH-aromatic), 3.91 (2H, s, S-CH_2_); ^13^C-NMR(150 MHz; DMSO-*d*_6_) *δ*_C_: 160.17 (C=O), 157.11 (C=N), 146.25, 139.78, 136.46, 135.51, 132.34, 131.69, 131.07, 130.59, 128.72, 127.15, 125.89, 121.35 (11 × CH-aromatic, 7 × C_q_-aromatic, and CF_3_), 35.43 (CH_2_); MS (EI) *m/z* (%) [M^+^ + 1, ^37^Cl] 485.19 (4.80), [M ^+^, ^37^Cl] 484.08 (16.22), [M^+^ + 3, ^35^Cl] 483.08 (23.67), [M^+^ + 2, ^35^Cl] 482.07 (79.35), [M^+^ + 1, ^35^Cl] 481.21 (60.81), [M^+^, ^35^Cl] 480.14 (100.00) for C_22_H_13_Cl_2_F_3_N_2_OS, 479.59 (35.24), 478.93 (10.15), 449.00 (52.76), 447.10 (72.05), 412.25 (7.68), 355.00 (34.48), 337.13 (4.68), 335.03 (3.09), 322.23 (1.40), 320.10 (2.93), 304.29 (1.46), 268.93 (3.28).

*2-(2-Bromo-5-methoxybenzylsulfanyl)-6-bromo-3-(4-trifluoromethyl-phenyl)-3H-quinazolin-4-one* (**5e**), off-white solid (EtOH/CHCl_3_), yield (93%), m.p. 206–207 °C; ν_max_ (KBr)/cm^−1^ 3067 (CH-aromatic), 2929 and 2862 (CH-aliphatic), 1691 (C=O), 1544 (C=N), 1462 (C=C), 1326, 1241, 1203, 1161, 1120, 1063, 1018, 967, 832, 660; ^1^H-NMR (500 MHz; CDCl_3_) *δ*_H_: 8.33 (1H, d, *J* = 2.5 Hz, CH-aromatic), 7.84 (1H, dd, *J* = 8.5, 2.5 Hz, CH-aromatic), 7.78 (2H, d, *J* = 8.0 Hz, 2 × CH of 4-CF_3_-C_6_H_4_), 7.57 (1H, d, *J* = 8.5 Hz, CH-aromatic), 7.43–7.40 (3H, m, 3 × CH-aromatic), 7.13 (1H, d, *J* = 3.0 Hz, CH-aromatic), 6.68 (1H, dd, *J* = 9.0, 3.0 Hz, CH-aromatic), 4.50 (2H, s, S-CH_2_), 3.74 (3H, s, OCH_3_); ^13^C-NMR (125 MHz; CDCl_3_) *δ*_C_: 160.39 (C=O), 158.83 (C=N), 156.73, 146.43, 138.48, 138.13, 136.47, 133.49, 129.79, 128.03, 127.01, 126.98, 124.63, 122.46, 121.10, 119.39, 117.31, 115.24, 114.92 (10 × CH-aromatic, 8 × C_q_-aromatic, and CF_3_), 55.45 (OCH_3_), 37.52 (CH_2_); MS (EI) *m/z* (%) [M ^+^, ^81^Br] 600.15 (40.51), [M^+^ + 1, ^79^Br] 599.29 (19.34), for C_23_H_15_Br_2_F_3_N_2_O_2_S, 566.81 (89.90), 552.96 (73.88), 520.49 (26.10), 467.86 (43.53), 440.74 (47.66), 437.83 (20.21), 422.12 (28.05), 337.38 (78.36), 290.43 (21.89), 222.37 (26.16), 202.73 (58.44), 201.11 (32.20), 191.45 (81.52), 186.76 (23.12), 156.47 (27.10), 144.50 (58.73), 116.18 (64.60), 106.18 (28.17), 87.12 (64.64), 74.20 (60.82), 69.66 (16.26), 57.86 (33.61).

*2-(2-Bromo-5-methoxy-benzylsulfanyl)-6-chloro-3-(4-nitro-phenyl)-3H-quinazolin-4-one* (**5f**), white powder (MeOH/CHCl_3_), yield (67%), m.p. 215–216 °C; ν_max_ (KBr)/cm^−1^ 3082 (CH-aromatic), 2962 and 2862 (CH-aliphatic), 1682 (C=O), 1548 (C=N), 1520 and 1469 (C=C), 1399, 1347, 1304, 1246, 1199, 1164, 1111, 1018, 968, 919, 840, 810, 730, 698, 644, 597, 542, 496, 465, 424; ^1^H-NMR (300 MHz; DMSO-*d*_6_) *δ*_H_: 8.40 (2H, d, *J* = 9.0 Hz, 2 × CH-aromatic), 8.00 (1H, d, *J* = 2.4 Hz, CH-aromatic), 7.96–7.74 (4H, m, 4 × CH-aromatic), 7.48 (1H, d, *J* = 9.0 Hz, CH-aromatic), 7.30 (1H, d, *J* = 3.3 Hz, CH-aromatic), 6.80 (1H, dd, *J* = 8.7, 3.3 Hz, CH-aromatic), 4.51 (2H, s, S-CH_2_), 3.72 (3H, s, OCH_3_); ^13^C-NMR (150 MHz; CDCl_3_) *δ*_C_: 160.38 (C=O), 158.82 (C=N), 155.87, 148.63, 146.01, 140.88, 136.29, 135.56, 133.51, 132.00, 130.58, 127.92, 126.61, 125.07, 120.60, 117.36, 115.21, 114.95 (10 × CH-aromatic, 8 × C_q_-aromatic), 55.45 (OCH_3_), 37.54 (CH_2_); MS (EI) *m/z* (%) [M^+^ + 1, ^81^Br and ^37^Cl] 536.07 (5.84), [M^+^, ^81^Br and ^37^Cl] 535.04 (32.01), [M^+^ + 1, ^81^Br and ^35^Cl] 534.07 (34.13), [M^+^, ^81^Br and ^35^Cl] 533.05 (100.00), [M^+^ + 1, ^79^Br and ^35^Cl] 532.09 (30.17), [M^+^, ^79^Br and ^35^Cl] 531.07 (90.35), for C_22_H_15_^81^BrClN_3_O_4_S, 502.04 (8.58), 500.03 (13.17), 499.10 (5.81), 453.69 (4.52), 378.36 (1.34), 345.05 (2.04), 286.12 (1.85), 254.15 (1.48), 201.06 (2.19), 120.84 (1.31), 77.21 (3.92), 76.17 (1.32), 63.14 (1.28).

*2-(3-Nitro-benzylsulfanyl)-6-bromo-3-(4-nitro-phenyl)-3H-quinazolin-4-one* (**5g**), white powder (MeOH/CHCl_3_), yield (67%), m.p. 251–252 °C, ν_max_ (KBr)/cm^−1^ 3118 and 3065 (CH-aromatic), 2929 and 2856 (CH-aliphatic), 1690 (C=O), 1548 (C=N), 1518 and 1464 (C=C), 1349, 1264, 1199, 1098, 1013, 967, 899, 856, 826, 750, 719, 695, 656, 528, 415; ^1^H-NMR (300 MHz; DMSO-*d*_6_) *δ*_H_: 8.47-8.35 (3H, m, 3 × CH-aromatic), 8.15–8.00 (3H, m, 3 × CH-aromatic), 7.95 (1H, d, *J* = 7.2 Hz, CH-aromatic), 7.83 (2H, d, *J* = 8.7 Hz, 2 × CH-aromatic), 7.69 (1H, d, *J* = 8.7 Hz, CH-aromatic), 7.59 (1H, t, *J* = 7.8 Hz, CH-aromatic), 4.56 (2H, s, S-CH_2_); ^13^C-NMR (75 MHz; DMSO-*d*_6_) *δ*_C_: 159.65 (C=O), 156.40 (C=N), 148.46, 147.66, 146.18, 141.43, 139.87, 138.03, 136.40, 131.40, 129.94, 128.72, 128.52, 124.95, 124.45, 122.35, 121.50, 118.46 (11 × CH-aromatic and 7 × C_q_-aromatic), 34.96 (CH_2_); MS (EI) *m/z* (%) [M^+^ + 2, ^81^Br] 517.03 (1.36), [M^+^, ^81^Br] 515.08 (18.24), [M^+^ + 1, ^79^Br] 514.56 (12.55), [M^+^, ^79^Br] 513.11 (19.77) for C_21_H_13_BrN_4_O_5_S, 511.51 (41.12), 497.04 (73.71), 495.04 (63.14), 467.95 (33.54), 434.27 (2.01), 387.96 (5.03), 376.89 (6.86), 346.06 (16.36), 331.06 (9.28), 311.17 (4.59), 304.07 (31.73), 300.12 (20.05), 266.70 (4.79), 223.20 (7.92), 207.82 (10.64), 196.83 (1.84), 136.18 (46.62), 122.16 (9.11), 116.19 (1.37), 105.16 (17.00), 90.17 (100.00), 89.16 (77.70), 77.16 (35.65).

*2-(4-Chloro-benzylsulfanyl)-6,7-dimethoxy-3-(4-nitro-phenyl)-3H-quinazolin-4-one* (**5h**), yellow powder (EtOH/CHCl_3_), yield (77%), m.p. 250–251 °C; ν_max_ (KBr)/cm^−1^ 3072 (CH-aromatic), 2960 and 2833 (CH-aliphatic), 1684 (C=O), 1610 (C=N), 1554; 1518 and 1495 (C=C), 1461, 1437, 1388, 1356, 1308, 1277, 1240, 1210, 1175, 1149, 1075, 1009, 971, 868, 840, 774, 749, 711, 602, 502, 430; ^1^H-NMR (300 MHz; DMSO-*d*_6_) *δ*_H_: 8.40 (2H, d, *J* = 5.4 Hz, 2 × CH-aromatic), 7.81 (2H, d, *J* = 5.4 Hz, 2 × CH-aromatic), 7.56-7.12 (6H, m, 6 × CH-aromatic), 4.44 (2H, s, S-CH_2_), 3.98 (3H, s, OCH_3_), 3.86 (3H, s, OCH_3_); ^13^C-NMR (150 MHz; DMSO-*d*_6_) *δ*_C_: 160.15 (C=O), 155.65 (C=N), 153.73, 148.60, 143.93, 142.25, 136.29, 132.42, 131.77, 131.67, 128.82, 125.12, 112.61, 107.70, 106.14 (10 × CH-aromatic and 8 × C_q_-aromatic), 56.65 (OCH_3_), 56.28 (OCH_3_), 35.41 (CH_2_); MS (EI) *m/z* (%) [M^+^ + 1, ^37^Cl] 486.20 (18.25), [M^+^, ^37^Cl] 485.02 (33.61), [M^+^ + 1, ^35^Cl] 484.03 (46.89), [M^+^, ^35^Cl] 483.03 (100.00) for C_23_H_18_ClN_3_O_5_S, 481.05 (15.67), 467.98 (2.89), 452.25 (13.58), 450.37 (28.43), 437.86 (2.75), 421.58 (1.59), 407.04 (7.06), 373.34 (4.96), 361.05 (12.05), 348.66 (8.75), 331.55 (12.43), 329.19 (53.67), 295.92 (2.68), 282.07 (3.70), 258.41 (2.80), 158.09 (1.56), 143.15 (2.27), 125.08 (2.33), 76.29 (2.21), 63.01 (2.86).

### 3.2. Biology

#### 3.2.1. DPPH Radical Scavenging Assay

The antioxidant potencies of all synthesized compounds were investigated using a 1,1 diphenyl-2-picrylhydrazyl (DPPH) radical scavenging assay according to the protocol described by Bersuder et al. [105]. In brief, a 0.5 mL volume of DPPH ethanolic solution was mixed with an equal volume of each sample concentration (i.e., the test compound or butylated hydroxytoluene, BHT, which was used as the reference antioxidant in the positive control experiment), shaken strongly, and incubated at room temperature for 1 h in darkness. The absorbance of the residual DPPH radicals was measured at λ = 519 nm and compared to the negative control (without the test compound or reference antioxidant). The DPPH radical scavenging power was calculated using the following formula: scavenging efficiency (%) = (1 − A_compound_/A_Control_) × 100, where A_compound_ and A_control_ are the absorbance of the tested compound and the negative control, respectively. The standard curve was obtained by plotting the scavenging effect (%) versus the compound concentration and it was used to determine the compound’s concentration providing 50% inhibition (IC_50_) in mg/mL, which was converted into mM units. Additionally, the ± SD values were calculated from three independent triplicate measurements.

#### 3.2.2. COX-2 Inhibition Assay

Each compound of the two series, **3a**–**h** and **5a**–**h**, was resuspended in 100% DMSO at a final concentration of 10 mg/mL. Each compound was investigated in triplicates at different concentrations (50, 100, 200 and 300 µg/mL) using a commercial COX-2 inhibitory screening assay kit (Catalog Number: 701080; Cayman Chemical Company) following the manufacturer’s instructions. Briefly, 10 µL of the tested compound was added to a mixture of the reaction buffer solution (160 µL of 0.1 M Tris-HCl, pH 8.0, in the presence of 2 mM phenol and 5 mM EDTA), COX-2 (10 µL) and heme (10 µL). After a 10 min incubation at 37 °C, the reaction was started by adding arachidonic acid (10 µL), rapidly mixed and kept at 37 °C for 2 min. PGF2α generated via PGH2 reduction with stannous chloride (30 µL) was incubated at room temperature for 5 min. The PGF_2α_ amount was then determined via an enzyme immunoassay. Prior to incubation with the COX-2 reaction (at room temperature 18 h), the 96-well plate was previously coated with a mouse anti-rabbit monoclonal antibody. Unbound reagents were removed by washing the plate followed by adding Ellman’s reagent to the well. The reaction product displaying a distinct yellow color was measured at 410 nm using a UV microplate reader. The % inhibition was calculated by comparing the PGF2α produced in the compound-treated samples with a control sample, using the following equation: % inhibition is equal to [([PGF_2α_]_control_ − [PGF_2α_]_sample_) /[PGF_2α_ ]_control_ ] × 100. Celecoxib was used as the standard inhibitor for COX-2. The COX-2 inhibitory efficiency was expressed as an inhibition percentage, which was determined via a comparison with the negative control experiments. The IC_50_ values were deduced for the most active candidates from their calibration curves.

#### 3.2.3. Lactate Dehydrogenase A (LDHA) Inhibitory Assay

The LDHA inhibitory effectiveness was investigated by measuring the amounts of consumed NADH [73]. Briefly, different concentrations of each compound (50, 100, 200 and 300 µg/mL) were incubated in a buffer containing 20 mM of HEPES-K+ (pH 7.2), 20 μM of NADH, 2 mM of pyruvate and 10 ng of purified recombinant human LDHA protein for 10 min. The fluorescence of NADH, which has an excitation wavelength of 340 nm and an emission wavelength of 460 nm, was detected using a spectrofluorometer. Sodium oxamate at 1 mM was used as a standard inhibitor of LDHA. The LDHA inhibitory efficiency was expressed as an inhibition percentage, which was determined via a comparison with the negative control experiments. The IC_50_ values were calculated for the most active candidates from their calibration curves.

#### 3.2.4. In Vitro α-Glucosidase (AG) Inhibitory Activity

The AG inhibitory activities of the synthesized quinazolinone derivatives were assessed based on the detection of the release of 4-nitrophenol (NP) from 4-nitrophenyl α-D-glucopyranoside (4-NPGP) using the method reported by Andrade-Cetto and collaborators [106]. Concisely, 20 µL of each compound at different concentrations ranging from 0 to 50 µg/mL, DMSO or quercetin (used as the positive control) at the same concentration of the tested compounds was mixed with 180 µL of the α-glucosidase enzyme from *Saccharomyces Cerevisiae,* and the resulting mixture was incubated at 37 °C for 2 min. Then, the reaction was allowed to start by adding 150 µL of the color reagent 4-NPGP and the resulting mixture was incubated for additional 15 min at 37 °C. Collectively, the colorimetric assay medium contained 2U of α-glucosidase, 5 mM of 4-NPGP and 10 mM of the potassium phosphate buffer with a pH of 6.9. Reading the assay was carried out using a microplate reader (Bio-Tek ELX-800, Thermo Fischer Scientific, Agilent Technologies, New York, USA) at 405 nm. The α-glucosidase inhibition was calculated as follows: % inhibition = 100 − (X2 sample − X1 sample/ X2 control − X1 control) × 100, where X1 is the absorbance of the initial reading and X2 is the absorbance of the final reading, and control is the absorbance of the assay without the compound and with DMSO instead. The inhibitory efficiency of each compound was expressed as IC_50_ µg/mL (as well as µM), which was calculated from the corresponding calibration curve.

#### 3.2.5. In Vitro α-Amylase (AA) Inhibition Assay

In this experiment, the α-amylase inhibitory activities of the compounds **3a**-**h** and **5a**-**h** were evaluated according to the reported methodology [107]. Precisely, 10 µL of α-amylase enzyme (3,3 U, EC 3.2.1.1, Sigma Chemical Co., St. Louis, MO, USA) was mixed with 10 µL of each compound at different concentrations ranging from 20 to 200 µg/mL, DMSO or quercetin (used as the positive control at the same compound concentrations) at 37 °C for 5 min. Then, 180 µL of Labtest (the amylase substrate) was added and the samples were incubated for 8 min, followed by measuring the first reaction at 620 nm to obtain (X1). Afterwards, the reaction mixture was incubated for an additional 5 min at 37 °C; then, the second reaction was measured to obtain the final reading (X2). Notably, the amylase substrate, Labtest, was diluted in distilled water (1:1) before being added to the microplate (Bio Tek ELX-800, USA). The α-amylase inhibitory efficiency was calculated as follows: % inhibition = 100 − (X2 sample − X1 sample/ X2 control − X1 control) × 100, where control is the absorbance of the assay with DMSO instead the test compound. The results were expressed as IC_50_ µg/mL (as well as µM) and were determined from the standard curves.

#### 3.2.6. Viability Assay

The cytotoxic potencies of the studied quinazolinone derivatives were examined on two human colon cancer cell line types: HCT-116 and Lovo (American Type Culture Collection; Manassas, VA, USA), using various amounts of tested compounds (50, 100, 200 and 400 µg). The samples were diluted in Dulbecco’s Modified Eagle’s Medium, consisting of 10% fetal bovine serum (FBS), added to cells grown and cultured for 24 h in a 5% CO_2_-humidified incubator at 37 °C. Thereafter, the activity of lactate dehydrogenase released from damaged cells was determined [73] in the collected supernatant aliquots using an ELISA end-point assay (Benchmark Plus, Bio-Rad, CA, USA); 0.1% Triton X-100 in the assay medium and the assay medium only were used as the positive and negative controls, respectively. Cell viability was expressed as a relative percentage of the OD values (at 550 nm) for compound-treated cells (final concentration of 200 µg /mL) and the negative control, which was shown as the mean % ± SD (n = 3). The results of the viability assays for the potential cytotoxic candidates were compared with the % of viable human umbilical vein endothelial cells (HUVEC) at the same concentration. These cells were grown in Dulbecco’s Modified Eagle’s Medium (PAN-Biotech; Barcelona, Spain) containing 10% FBS (Sigma Aldrich; St. Quentin-Fallavier, France), 2 mM Glutamine (Sigma Aldrich), 100 units/mL penicillin and 100 mg/mL streptomycin (Life Technologies; Paisley, UK).

A plot of the cell viability (%) versus the compound concentration (µg/mL) was also performed to determine the compound concentration providing 50% inhibition (IC_50_) in µg/mL, which was converted to µM.

#### 3.2.7. Statistical Analysis

The obtained results were presented as the mean ± standard deviation (SD) from three experiments. The statistical significance between pairs of means was evaluated using Student’s t test. A value of p\0.05 or p\0.01 was the criteria for a statistical significance.

The IC_50_ value for each tested compound was obtained using the Quest Graph™ IC_50_ Calculator, AAT Bioquest, Inc. (https://www.aatbio.com/tools/ic50-calculator) online resource accessed on 2 February 2023.

#### 3.2.8. Determination of the Expressional Levels of Bax, caspase-3 and Bcl-2 Genes

##### Cell Culture

Briefly, in a 25 mL flask (1 × 10^6^ cells/flask), the human colon cancer cell lines (HCT-116 and LoVo) were grown for 24 h. After replacing the culture media with a medium containing 1% FBS for 24 h, the cells were treated with 10 µg/mL of each tested compound in 2 mL of fresh medium containing 1% FBS, or 0.25% DMSO, which was used as the negative control. The cells were adherent cells and they were detached using trypsin after being washed with ice-cold phosphate-buffered saline (PBS).

##### Design of the Primer

Primers specific for the Bax, Bcl-2 and Caspase-3 genes were designed using the primer blast (https://www.ncbi.nlm.nih.gov/tools/primer-blast/ accessed on 9 January 2023) software. The primers were used in the RT-qPCR analysis to amplify fragments of 100–200 bp in length (Table 11).

##### Reverse-Transcription PCR (RT-PCR)

The RNeasy Mini Kit (Qiagen, Hilden, Germany) was used to extract the total RNA. The RNA purity, quality and quantity were checked using a Nanodrop 8000 spectrophotometer (Thermo Fisher Scientific, USA). The Hyperscript Kit (GeneAll, Seoul, Korea) and random hexamers (GeneAll, Seoul, Korea ) were used to reverse-transcript 1 µg of RNA from each sample into cDNA as described in the manufacturer’s protocol.

##### Quantitative Real-Time PCR

A QuantStudio 7Flex Detection System (Applied Biosystems, Thermo Fischer Scientific, Inc., USA) was used to measure the mRNA expression of Bax, Caspase-3 and Bcl-2. The reactions for the synthesis of cDNA were carried out using an iScript One-Step RT-PCR Kit with SYBR Green (cat no: 170–8892; Bio-Rad, Inc., Hercules, CA, USA). Briefly, primers (Table 10) were added to the reaction mixture at a final concentration of 10 pM. Thus, 5 µL of each cDNA sample were added to a 20 µL PCR mixture consisting of 12.5 µL of 2 × iScript One-Step RT-PCR Kit with SYBR Green, 0.5 µL of primers for Bax, Caspase-3 and Bcl-2 as well as GAPDH (Macrogen, Seoul, Korea), and 7 µL RNase/DNase-free water (Qiagen, DE). The thermal cycling conditions for Bax, Caspase-3 and Bcl-2 were established as 5 min at 95 °C, followed by 40 cycles of 30 s at 95 °C and 30 s at 60 °C, and finally 10 s at 95 °C. The presence of a single melting temperature peak verified the specificity of each primer. The expression of a house-keeping gene, GAPDH, was used as an endogenous control for the current work.

#### 3.2.9. Annexin-V-FITC Assay

The distribution of early and late apoptotic cells after treatment with compounds **3a** and **3f** was carried out using an Annexin V-FITC Apoptosis Detection Kit (BioVisionResearch Products—USA). HCT-116 and LoVo carcinoma cells were seeded in 60 mm^2^ culture dishes and treated with the vehicles DMSO, and compounds **3a** and **3f** (50 µg/mL) for 48 h. Cells were then harvested, washed twice with PBS and resuspended in an Annexin-V binding buffer (BioVisionResearch Products—USA). Finally, the cells were stained with Annexin V-FITC and propidium iodide (PI). The fluorescent intensity of the stained cancer cells was determined using an FITC signal detector (usually FL1) and PI staining with the phycoerythrin emission signal detector (through quadrant statistics for necrotic and apoptotic cell populations).

#### 3.2.10. Cell Cycle Assay

Changes in cell cycle distribution induced by compounds **3a** and **3f** were analyzed using a flow cytometric analysis according to Nicoletti et al. 1991 [108] using a Propidium Iodide Flow Cytometry Kit for Cell Cycle Analysis (Abcam-UK). HCT-116 and LoVo carcinoma cells (5 × 10^4^ cells/mL) were treated with DMSO as a negative control, and compounds **3a** and **3f** (50 µg/mL), for 48 h. After the centrifugation of the treated cells at 1800 rpm for 5 min, the pellet was washed twice with PBS. Then, the cells were fixed by mixing 700 mL of 90% cold ethanol, and stained with propidium iodide (PI) for 1 h at 37 °C. RNase A at 10 mg/mL was added in order to limit the ability of the PI to bind only to the DNA molecules. The stained cells were analyzed for their DNA content using a BD FACSC flow cytometer.

#### 3.2.11. Immunoblot Experiments

In order to determine the levels of production of caspases 8 and 9 by the treated carcinoma cells, Western blot analysis was carried out using a FLAG^®^ Western Detection Kit (Agilent Technologies, USA). Exponentially growing HCT-116 and LoVo cells were treated with 50 µg/mL of derivatives **3a** and **3f** for 48 h. After incubation, the cells were harvested and lysed using a Lysis buffer consisting of 10 mM of Tris, 100 mM of NaCl, 25 mM of ethylenediaminetetra aceticacid (EDTA), 25 mM of Ethylene glycol bis(2-aminoethyl)tetraacetic acid (EGTA), 0.1% Sodium dodecyl sulfate (SDS), 1% (*v*/*v*) Triton X-100 and 2% (*v*/*v*) NP-40 (pH 7.4), with a 1:200 protease inhibitor cocktail (Sigma-Adrich) and a 1:300 phosphatase inhibitor cocktail tablet (Roche Applied Science, Mannheim, Germany). The protein concentrations of each sample were determined calorimetrically using the Bradford method before proceeding to carry out the Western blotting [109]. Equal amounts (20 µg) of the protein samples were mixed and boiled with SDS Loading buffer for 10 min, allowed to cool on ice and then loaded into SDS-polyacrylamide gel and separated using a Cleaver electrophoresis unit (Cleaver, Warwickshire, UK), before being transferred onto polyvinylidene fluoride (PVDF) membranes (Bio-Rad, USA) for 30 min using a Semi-dry Electroblotter (Biorad, USA) at 2.5 A and 25 V for 30 min. The membrane was blocked with 5% nonfat dry milk in TBS-T for two hours at room temperature, in order to reduce non-specific protein interactions between the membrane and the antibody. The blocked membrane was incubated overnight at 4 °C with primary antibodies (Cell Signaling Technology, Massachusetts, USA) and β-actin (Sigma). The blots were then washed three times (10 min each) with TBS-T. The membrane was then incubated with the corresponding horse radish peroxidase (HRP)-linked secondary antibodies (Dako, Hamburg, Germany) for another hour at room temperature, followed by washing three times (10 min each) with TBS-T. The chemiluminescent Western ECL substrate (Perkin Elmer, Waltham, MA, USA) was applied to the blot according to the manufacturer’s recommendation. Briefly, the membranes were incubated for 1 min with a mixture of equal volumes of ECL solution A and ECL solution B. The chemiluminescent signals were captured using a CCD camera-based imager (Chemi Doc imager, Biorad, USA), and the bands’ intensities were then measured using ImageLab v3.0 (Bio-Rad). Protein-sized markers were used in all gels to localize the gel transfer regions for specific proteins and determine the transfer efficiency. Following an overnight incubation with the primary antibodies, HRP conjugated secondary antibodies were added and incubated for 1 h. The proteins were detected using an enhanced chemiluminescence (ECL) detection system (Thermo Fischer Scientific, USA). The intensities of the bands were quantified using the NIH ImageJ software v3.0.1 (http://rsb.info.nih.gov/ij/).

### 3.3. In Silico Studies

#### 3.3.1. Molecular Docking Studies

- Preparation of the protein

The crystal structures of the protein–ligand complexes for alpha-amylase (PDB ID: 3BAJ) (https://doi.org/10.1021/bi701652t) accessed on 5 March 2023, human lysosomal acid-alpha-glucosidase (PDB ID: 5NN5) (https://doi.org/10.1038/s41467-017-01263-3) on 5 March 2023, lactate dehydrogenase A (PDB ID: 1I10) (https://doi.org/10.1002/1097-0134(20010501)43:2<175:aid-prot1029>3.0.co;2-#) on 24 May 2023 and cyclooxygenase-2 (PDB ID: 3LN1) (https://doi.org/10.1016/j.bmcl.2010.07.054) 25 May 2023 were used for the docking calculations. They were retrieved from the Research Collaboratory for Structural Bioinformatics (RCSB) Protein Data Bank. For each crystal structure, the crystallographic water molecules were removed, the missing hydrogen atoms were added and the inhibitor from the crystal structure was used to define the active site.

- Preparation of ligands

The structures of all the quinazolinone compounds were sketched using Chemdraw ultra 13.0 and converted into 3D structures using the Hyperchem pro 8.0 software (www.hyper.com). Geometry optimization was carried out using the PM3 method via the MOPAC program (http://OpenMOPAC.net). Finally, all the compounds were saved in a .pdb format for further docking studies.

- Docking studies

Autodock tools (ADT) version 1.5.6 (www.autodock.scrips.edu) was used to prepare the molecular docking. The Lamarckian genetic algorithm methodology was employed for the docking simulations. The best binding conformation was selected from the docking log (.dlg) file for each ligand and further interaction analysis was performed using PyMol and Discovery Studio Visualizer 4.0.

#### 3.3.2. ADMET Analysis

The ADMET analysis was performed using the standard protocol recommended in ADMETLab 2.0 (https://admetmesh.scbdd.com) [89]. For this, the canonical SMILES (simplified molecular-input line-entry system) of all molecules were submitted serially to the ‘ADMET Evaluation’ option of the ‘services’ section of the ADMETLab 2.0 server. After submitting, the server led to the calculation and display of a good number of the ADMET properties of a molecule. The results were then saved in a ‘.csv’ file. The same file is available as a Supplementary Material.

#### 3.3.3. The Bioavailability Radar Charts

These were studied using the SwissADME free web tool [95] (http://www.swissadme.ch/idex.php), accessed on 26 May 2023.

## 4. Conclusions

Quinazolinone derivatives **3a**–**h** and their *S*-arylated analogues **5a**–**h** were synthesized and characterized by various spectroscopic techniques. Multi-target biological screening revealed the significance of **3a**, **3g** and **5a** as potent DPPH radical scavengers with lowered IC_50_ values (mM) of 0.191 ± 0.011, 0.165 ± 0.005 and 0.172 ± 0.004, respectively, as compared with 0.245 ± 0.027 by BHT. Despite this, none of the screened compounds showed an improved inhibitory efficiency to celecoxib (IC_50_ = 0.136 × 10 ^−3^ ± 0.006 × 10 ^−3^ µM) and sodium oxamate (IC_50_ = 140.503 ± 7.647 µM) against COX- 2 and LDHA, respectively; quinazolinones **3a** (IC_50_ values = 281.374 ± 10.545 and 273.345 ± 16.087 µM) and **3g** (IC_50_ values = 251.780 ± 22.023 and 242.279 ± 31.298) were recognized as the most active derivatives against these enzymes, respectively. Notably, quinazolinones **3h**, **5a** and **5h** with IC_50_ values ranging from 12.548 ± 0.542 to 12.882 ± 0.426 µM against AG, and derivatives **3a**, **3c**, **3f**, **3h** and **5a**–**5f** as well as **5h**, with IC_50_ values spanning from 186.437 ± 9.700 to 381.335 ± 8.713 µM against AA, exerted superior activity to quercetin (IC_50_ = 13.126 ± 0.688 and 402.566 ± 10.108, respectively). Therefore, **3h**, **5a** and **5h** can be considered as dual inhibitors for AG and AA.

In addition, the viability assays identified **3a** and **3f** as the most active antiproliferative agents with IC_50_ (µM) values of 294.324 ± 8.409 and 383.521 ± 8.989 (LoVo cells), and 298.060 ± 13.247 and 323.596 ± 2.996(HCT-116 cells) as compared with 22.371 ± 0.215(LoVo) and 91.098 ± 2.721(HCT-116) by 5-FU. Interestingly, these compounds did not inhibit the growth of non-tumoral HUVEC at 560.617 and 599.251 µM, with viability percents of 97.000 ± 2.646 and 99.667 ± 0.577, respectively. Quantitative real-time PCR measurements confirmed the capability of **3a** and **3f** at 10 µg/mL to upregulate the expression levels of Bax and Caspase-3 genes, along with the downregulation of the expression level of the Bcl-2 gene in the cultures of the treated HCT-116 and LoVo cells as compared to the negative control cultures. Moreover, the Western blotting analyses indicated that **3a** and **3f** were able to induced apoptosis in HCT-116 and LoVo colon cancer cells via pathways triggered by caspases, and predominantly through the intrinsic pathway mediated by caspase-9. Furthermore, the two compounds induced more death for the HCT-116 and LoVo cells via apoptosis than via necrosis; in addition, they induced cell cycle arrest for the LoVo cells in the G2/M phase and for the HCT-116 cells in the G1 phase. Therefore, the observed cytotoxicity of these derivatives could be attributed to the induction of apoptosis and the blocking of cell cycle progression in both cell lines. Molecular docking analyses of the active or potential candidates corroborated the observed inhibitory efficiencies in the in vitro assays against AA, AG, LDAH and COX-2. Lastly, the physicochemical properties, the suitability in medicinal chemistry and the ADMET characteristics were predicted for all the synthesized compounds and explained in detail for some representative active compounds. Collectively, these quinazolinone derivatives may be considered to be suitable candidates for further optimization and exploration against CRC and its risk factors, particularly oxidative stress and DM-type 2.

## Figures and Tables

**Table 1:** Mean IC_50_ values in mg/mL (mM) for scavenging initial DPPH radicals using the synthesized compounds ± SD of three independent replicates. BHT was used as the reference drug.

Comp. # (MW)	Mean Values of Half Maximal Inhibitory Potency in mg/mL ± SD (mM)
**3a** (356.75)	**0.068 ± 0.004 (0.191 ± 0.011)**
**3b** (401.20)	0.161 ± 0.016 (1.520 ± 0.039)
**3c** (382.36)	0.100 ± 0.014 (0.262 ± 0.037)
**3d** (356.75)	0.179 ± 0.016 (0.502 ± 0.045)
**3e** (401.20)	0.290 ± 0.028 (0.723 ± 0.070)
**3f** (333.75)	0.227 ± 0.010 (0.680 ± 0.030)
**3g** (378.20)	**0.0625 ± 0.002 (0.165 ± 0.005)**
**3h** (359.36)	0.325 ± 0.007 (0.904 ± 0.0195)
**5a** (464.86)	**0.080 ± 0.002 (0.172 ± 0.004)**
**5b** (525.77)	0.167 ± 0.010 (0.318 ± 0.019)
**5c** (517.48)	0.186 ± 0.006 (0.359 ± 0.012)
**5d** (481.31)	0.200 ± 0.028 (0.416 ± 0.058)
**5e** (600.25)	0.225 ± 0.021 (0.375 ± 0.035)
**5f** (532.79)	0.241 ± 0.013 (0.452 ± 0.024)
**5g** (513.32)	0.200 ± 0.014 (0.390 ± 0.027)
**5h** (483.92)	0.364 ± 0.020 (0.752 ± 0.041)
BHT (220.35)	**0.054 ± 0.006 (0.245 ± 0.027)**

**Table 2:** COX-2 inhibitory potency of the synthesized compounds expressed as mean % inhibition ± SD of three independent replicates. The increasement in inhibitory efficiency by duplicating the concentration was obtained by dividing the % inhibition at 0.200 mg/mL by the % inhibition at 0.100 mg/mL and was expressed in fold. Celecoxib was used as the reference COX-2 inhibitor at 0.1 mg/mL concentration.

Comp. #	Mean Values of % Inhibition ± SD	
	At 0.100 mg/mL	At 0.200 mg/mL (Fold)
**3a**	**57.000 ± 2.828**	**97.050 ± 1.344 (1.70)**
**3b**	6.250 ± 1.768	10.750 ± 2.475 (1.72)
**3c**	9.850 ± 1.626	20.200 ± 1.131(2.05)
**3d**	20.200 ± 1.131	32.750 ± 2.475 (1.62)
**3e**	10.400 ± 1.697	15.650 ± 0.494975 (1.50)
**3f**	11.450 ± 0.778	26.750 ± 3.889 (2.34)
**3g**	**60.000 ± 2.828**	**98.900 ± 1.556 (1.65)**
**3h**	14.550 ± 0.636	27.650 ± 2.192 (1.900)
**5a**	**34.200 ± 1.697**	**63.150 ± 5.869 (1.85)**
**5b**	9.250 ± 1.768	19.500 ± 2.121 (2.11)
**5c**	18.650 ± 3.465	26.250 ± 4.031 (1.41)
**5d**	26.300 ± 1.839	44.050 ± 1.344 (1.68)
**5e**	31.000 ± 2.828	51.650 ± 4.879 (1.67)
**5f**	28.900 ± 2.687	52.000 ± 2.828 (1.80)
**5g**	14.250 ± 1.768	26.000 ± 2.828 (1.83)
**5h**	11.200 ± 0.990	28.900 ± 0.283 (2.58)
Celecoxib at 0.1 mg/mL	**100.000 ± 0.000**	**ND ^1^**

^1^ ND: not determined.

**Table 3:** LDHA inhibitory potency of the synthesized compounds at 0.1 mg/mL and 0.2 mg/mL concentrations expressed as mean % inhibition ± SD of three independent replicates. The increasement in inhibitory efficiency by duplicating the concertation was obtained by dividing the % inhibition at 0.200 mg/mL by % the inhibition at 0.100 mg/mL and was expressed in fold. Sodium oxamate was used as the reference drug at 0.111 mg/mL (1 mM) concentration.

Comp. #	Mean Values of % Inhibition ± SD	
	At 0.1 mg/mL	At 0.2 mg/mL (Fold Change as Compared to 0.1 mg/mL)
**3a**	53.500 ± 1.131	**98.450 ± 1.344 (1.84)**
**3b**	29.150 ± 2.192	42.350 ± 1.909 (1.45)
**3c**	17.800 ± 1.556	30.750 ± 1.7678 (0.2)
**3d**	33.500 ± 2.121	53.500 ± 2.121 (1.59)
**3e**	22.150 ± 1.202	35.950 ± 1.344 (1.62)
**3f**	21.500 ± 1.556	34.250 ± 1.768 (1.59)
**3g**	68.250 ± 4.596	**100.000 ± 0.000 (1.7)**
**3h**	29.850 ± 1.768	43.900 ± 1.556 (1.47)
**5a**	24.000 ± 1.697	38.900 ± 1.131 (1.62)
**5b**	14.500 ± 1.131	22.500 ± 1.697 (1.55)
**5c**	8.550 ± 1.061	14.300 ± 1.556 (1.67)
**5d**	13.850 ± 2.051	20.400 ± 1.980 (1.47)
**5e**	15.150 ± 1.768	19.650 ± 2.334 (1.29)
**5f**	31.950 ± 2.475	51.050 ± 3.606 (1.60)
**5g**	15.750 ± 2.050	27.700 ± 2.546 (1.75)
**5h**	15.350 ± 1.202	21.750 ± 1.344 (1.42)
Sodium Oxamate at 0.111 mg/mL (1 mM)	**100.000 ± 0.000**	ND ^1^

^1^ ND: not determined.

**Table 4:** Mean IC_50_ values of the potential COX-2 and LDHA inhibitors and their reference inhibitors, celecoxib and sodium oxamate, respectively, in µg/mL (µM) **±** SD of three independent replicates.

Comp. # (MW)	Mean IC_50_ Values in µg/mL ± SD/(µM ± SD)	
	COX-2	LDHA
**3a** (356.75)	100.380 ± 3.762/ (281.374 ± 10.545)	97.516 ± 5.739/ (273.345 ± 16.087)
**3g** (378.20)	95.223 ± 8.329/ (251.780 ± 22.023)	91.630 ± 11.837/ (242.279 ± 31.298)
Celecoxib (381.373)	0.0517 × 10 ^−3^ ± 0.0021 × 10 ^−3^/ (0.136 × 10 ^−3^ ± 0.006 × 10 ^−3^)	ND ^1^
Sodium oxamate (111.03)	ND ^1^	15.600 ± 0.849/ (140.503 ± 7.647)

^1^ ND: not determined.

**Table 5:** α-Glucosidase and α-amylase inhibitory potencies of the synthesized compounds expressed as mean IC_50_ values ± SD of three independent replicates. Quercetin was used as the reference inhibitor.

Comp. # (MW)	Mean IC_50_ Values in µg/mL ± SD (µM ± SD)	
	α-Glucosidase (AG)	α-Amylase (AA)
**3a** (356.75)	11.200 ± 0.300 (31.395 ± 0.841)	**124.333 ± 4.042 (348.516 ± 11.330)**
**3b** (401.20)	26.033 ± 0.503 (64.888 ± 1.254)	17171.333 ± 4.163 (427.051± 10.376)
**3c** (382.36)	7.867 ± 0.306 (20.5 ± 0.800)	**92.000 ± 3.000 (240.611 ± 7.846)**
**3d** (356.75)	37.200 ± 0.361 (104.275 ± 1.012)	335.667 ± 3.055 (940.903 ± 8.563)
**3e** (401.20)	30.500 ± 0.819 (76.022 ± 2.041)	252.00 ± 4.583 (628.116 ± 11.423)
**3f** (333.75)	5.233 ± 0.153 (15.679 ± 0.458)	**111.667 ± 3.512 (334.583 ± 10.523)**
**3g** (378.20)	12.500 ± 0.889 (33.051 ± 2.351)	179.333 ± 7.768 (474.175 ± 20.539)
**3h** (359.36)	**4.633 ± 0.153 (12.882 ± 0.426)**	**122.000 ± 4.583 (339.492 ± 12.753)**
**5a** (464.86)	**5.833 ± 0.252 (12.548 ± 0.542)**	**86.667 ± 4.509 (186.437 ± 9.700)**
**5b** (525.77)	37.033 ± 0.252 (70.436 ± 4.793)	**162.000 ± 5.568 (308.120 ± 10.590)**
**5c** (517.48)	41.367 ± 1.002 (79.939 ± 1.936)	**197.333 ± 4.509 (381.335 ± 8.713)**
**5d** (481.31)	38.100 ± 0.656 (79.159 ± 1.363)	**181.667 ± 5.508 (377.443 ± 11.444)**
**5e** (600.25)	43.133 ± 2.055 (71.858 ± 3.424)	**150.333 ± 3.512 (250.451 ± 5.851)**
**5f** (532.79)	23.600 ± 0.400 (44.295 ± 0.751)	**106.667 ± 5.033 (200.205 ± 9.446)**
**5g** (513.32)	20.633 ± 0.737 (40.195 ± 1.436)	339.000 ± 4.000 (660.407 ± 7.792)
**5h** (483.92)	**6.100 ± 0.458 (12.605 ± 0.946)**	**117.667 ± 3.055 (243.154 ± 6.313)**
Quercetin (302.236)	**3.967 ± 0.208 (13.126 ± 0.688)**	**121.667 ± 3.055 (402.566 ± 10.108)**

**Table 6:** Cytotoxic effects of the synthesized compounds on LoVo and HCT-116 human colon carcinoma cell lines expressed as mean % of viable cells after being treated with 200 µg/mL of the test compounds for 48 h ± SD of three independent assays. Assay medium and 0.1% Triton X-100 were used as negative and positive control experiments, respectively. The cytotoxic effects of the potential antiproliferative candidates were further assessed on non-tumoral human umbilical vein endothelial cells (HUVEC) at the same concentration.

Comp. #	Mean % of Viable Cells ± SD		
	LoVo	HCT-116	HUVEC
**3a**	**23.500 ± 1.500**	**26.833 ± 1.258**	**97.000 ± 2.646**
**3b**	62.000 ± 3.606	70.667 ± 2.517	ND ^1^
**3c**	**47.333 ± 2.517**	65.000 ± 3.606	**99.667 ± 0.577**
**3d**	87.000 ± 1.732	96.000 ± 1.732	ND ^1^
**3e**	90.667 ± 3.512	92.333 ± 2.517	ND ^1^
**3f**	**22.667 ± 2.082**	**4.667 ± 0.577**	**99.667 ± 0.577**
**3g**	55.667 ± 3.055	**42.333 ±** **2.517**	**98.667 ± 1.155**
**3h**	64.000 ± 3.000	70.333 ± 3.786	ND ^1^
**5a**	90.333 ± 1.528	89.667 ± 3.055	ND ^1^
**5b**	**37.667 ± 2.517**	60.333 ± 2.517	**96.333 ± 0.577**
**5c**	78.333 ± 3.512	82.333 ± 2.517	ND ^1^
**5d**	76.000 ± 1.732	86.667 ± 1.528	ND ^1^
**5e**	64.333 ± 2.082	79.667 ± 2.517	ND ^1^
**5f**	84.333 ± 3.512	76.000 ± 1.732	ND ^1^
**5g**	74.667 ± 2.517	66.333 ± 3.055	ND ^1^
**5h**	81.667 ± 3.055	91.000 ± 2.646	ND ^1^
Positive Control (Triton X-100, 0.1%)	0.000	0.000	ND ^1^
Negative Control (Assay medium) 5-FU	99.500 ± 0.707	100.000 ± 0.000	57.333 ± 2.517

^1^ ND: not determined.

**Table 7:** Mean IC_50_ values of the compounds, which exhibited cell viabilities of less than 50%, expressed in µg/mL (µM) ± SD of three independent replicates. 5-Flurouracil was used as the reference drug.

Comp. # (MW)	Mean IC_50_ Values in µg/mL/(µM) ± SD		
	LoVo Cells	HCT-116 Cells	HUVEC
**3a** (356.75)	105.000 ± 3.000/ (294.324 ± 8.409)	106.333 ± 4.726/ (298.060 ± 13.247)	
**3c** (382.36)	275.333 ± 4.509/ (720.088 ± 11.792)	281.000 ± 3.606/ (734.910 ± 9.431)	
**3f** (333.75)	128.000 ± 3.000/ (383.521 ± 8.989)	108.000 ± 1.000 (323.596 ± 2.996)	
**3g** (378.20)	221.667 ± 3.512/ (586.111 ± 9.286)	168.333 ± 3.512/ (444.090 ± 9.286)	
**5b** (525.77)	161.000 ± 3.606/ (306.218 ± 6.859)	246.000 ± 1.732/ (467.885 ± 3.294)	
5-FU (130.08)	2.910 ± 0.028/ (22.371 ± 0.215)	11.850 ± 0.354/ (91.098 ± 2.721)	298.500 ± 19.092 (2294 ± 146.77)

**Table 8:** Binding free energies of the docked conformations of the studied compounds in the active sites of α-Amylase (AA) and α-Glucosidase (AG).

Comp.#	Binding Energies (kcal/mol)	
	α-Amylase	α-Glucosidase
**3c**	−8.7	−5.6
**3f**	−8.4	−5.2
**3h**	−9.1	−4.8
**5a**	−8.9	−9.0
**5f**	−8.7	ND ^1^
**5h**	−8.4	−5.7
Quercetin	−9.3	−7.0
Acarbose	−5.4	^1^ ND
1-Deoxynojirimycin	^1^ ND	−5.7

^1^ ND: not determined.

**Table 9:** In silico predictions for molecular descriptors for derivatives **3a**, **3c**, **3f**, **3g**, **3h**, **5a**, **5b**, **5c**, **5d**, **5e**, **5f** and **5h**.

Comp.#	MW^1^	nHA^1^	nHD^1^	nRot^1^	TPSA^1^	LogS^1^	Logp^1^	LogD^1^	Fsp3	nRig
**3a**	356.00	3	1	2	37.79	−4.594	3.762	3.732	0.067	19
**3c**	382.06	5	1	4	56.25	−4.421	2.886	3.295	0.176	19
**3f**	333.00	6	1	2	80.83	−4.032	2.871	2.633	0	20
**3g**	376.95	6	1	2	80.93	−4.102	3.076	2.493	0	20
**3h**	359.06	8	1	4	99.39	−3.934	2.011	2.284	0.125	20
**5a**	464.04	3	0	5	34.89	−7.104	5.561	4.422	0.091	24
**5b**	523.96	3	0	5	34.89	−7.588	6.153	4.22	0.091	24
**5c**	517.09	8	0	8	96.49	−6.868	4.228	3.921	0.167	25
**5d**	480.01	3	0	5	34.89	−7.543	6.039	4.385	0.091	24
**5e**	597.92	4	0	6	44.12	−7.779	6.228	4.13	0.13	24
**5f**	530.97	7	0	6	87.26	−7.54	5.344	4.046	0.091	25
**5h**	483.07	3	0	7	96.49	−6.87	4.192	3.788	0.13	25

MW^1^: Molecular weight; nHA^1^: number of H-bond acceptors; nHD^1^: number of H-bond donors; TPSA^1^: topological polar surface area in Å²; LogS^1^: the logarithm of aqueous solubility value; LogP: the logarithm of *n*-octanol/water partition coefficient; LogD^1^: the logarithm of octanol/buffer (at pH 7.4) distribution coefficient; Fsp3: the number of sp3 hybridized carbons/total carbon count; nRig: number of rigid bonds.

**Table 10:** In silico predictions for medicinal chemistry characteristics.

Comp. #	QED ^1^	Lipinski	Pfizer	Golden Triangle
**3a**	0.641	Accepted	Rejected	Accepted
**3c**	0.64	Accepted	Accepted	Accepted
**3f**	0.441	Accepted	Accepted	Accepted
**3g**	0.419	Accepted	Accepted	Accepted
**3h**	0.437	Accepted	Accepted	Accepted
**5a**	0.197	Accepted	Rejected	Accepted
**5b**	0.21	Rejected	Rejected	Rejected
**5c**	0.136	Accepted	Accepted	Rejected
**5d**	0.233	Accepted	Rejected	Accepted
**5e**	0.179	Rejected	Rejected	Rejected
**5f**	0.131	Rejected	Accepted	Rejected
**5h**	0.153	Accepted	Accepted	Accepted

QED ^1^: the quantitative estimate of drug-likeness.

**Table 11:** Description of primers used for qPCR.

Gene	Primer Sequence (5′ to 3′)
Bax	F 5′-TCAGGATGCGTCCACCAAGAAG-3′,R 5′-TGTGTCCACGGCGGCAATCATC-3′.
Bcl-2	F 5′-ATCGCCCTGTGGATGACTGAGT -3′,R 5′-GCCAGGAGAAATCAAACAGAGGC-3′.
Caspase 3	F 5′-GGAAGCGAATCAATGGACTCTGG-3′,R 5’-GCATCGACATCTGTACCAGACC -3′.
GAPDH	F 5′-GTCTCCTCTGACTTCAACAGCG-3′ R 5′-ACCACCCTGTTGCTGTAGCCAA-3′

## Data Availability

Data is contained within the article and supplementary material.

## Supplementary Materials

The following supporting information can be downloaded at https://www.mdpi.com/article/10.3390/ph16101392/s1; The Excel file is for all physicochemical and ADMET predictions and the PDF file is for the spectroscopic analyses of the synthesized compounds.
