# Supplementary figures and images for: Synthesis and Biological Evaluation of Some New 3-Aryl-2-thioxo-2,3-dihydroquinazolin-4(1H)-ones and 3-Aryl-2-(benzylthio)quinazolin-4(3H)-ones as Antioxidants; COX-2, LDHA, α-Glucosidase and α-Amylase Inhibitors; and Anti-Colon Carcinoma and Apoptosis-Inducing Agents
Source: Pharmaceuticals (Basel). 2023 Oct 1;16(10):1392. doi: 10.3390/ph16101392 (PMC10610505; doi:10.3390/ph16101392)

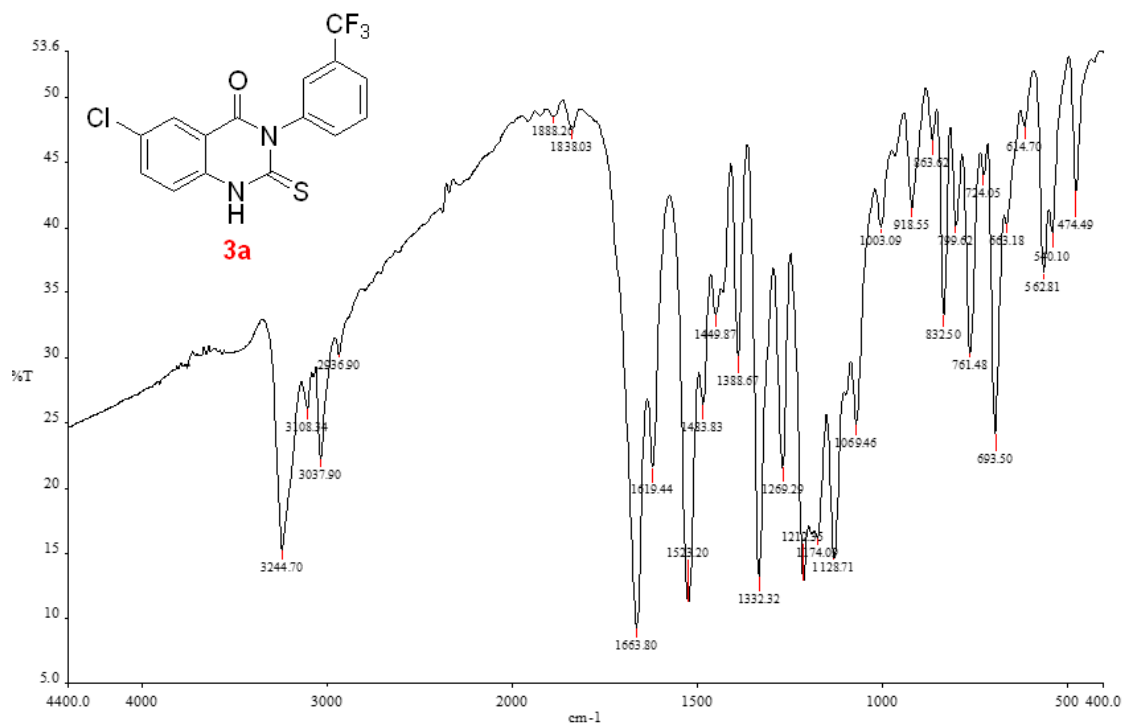

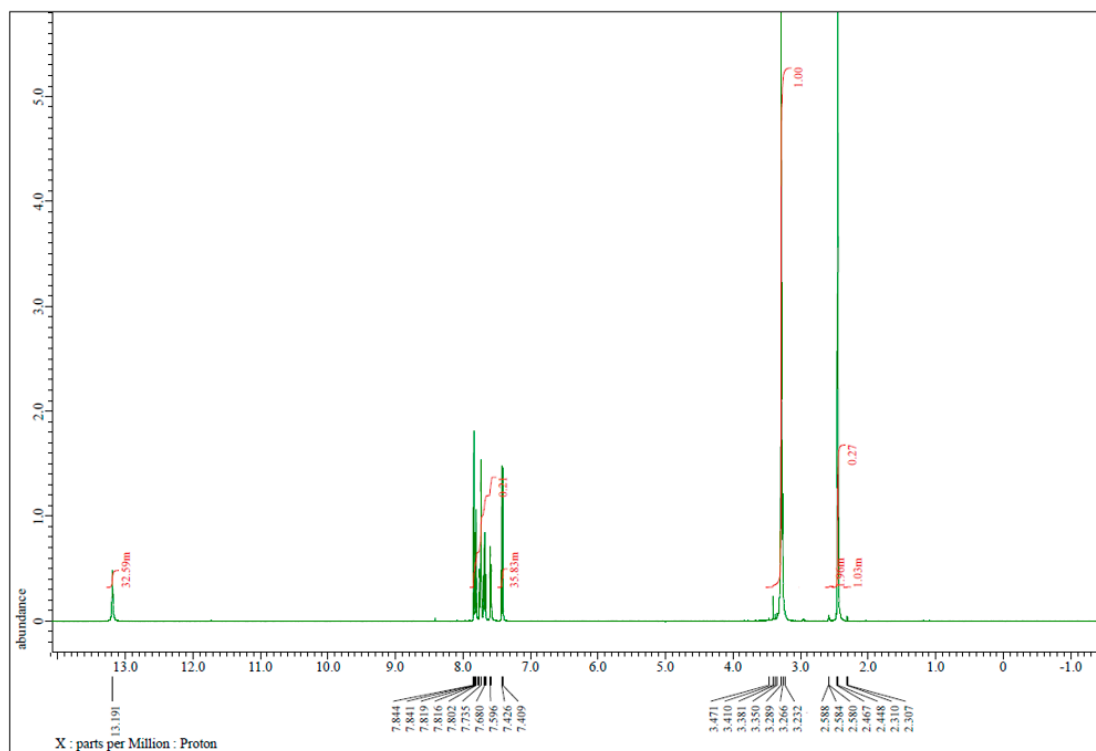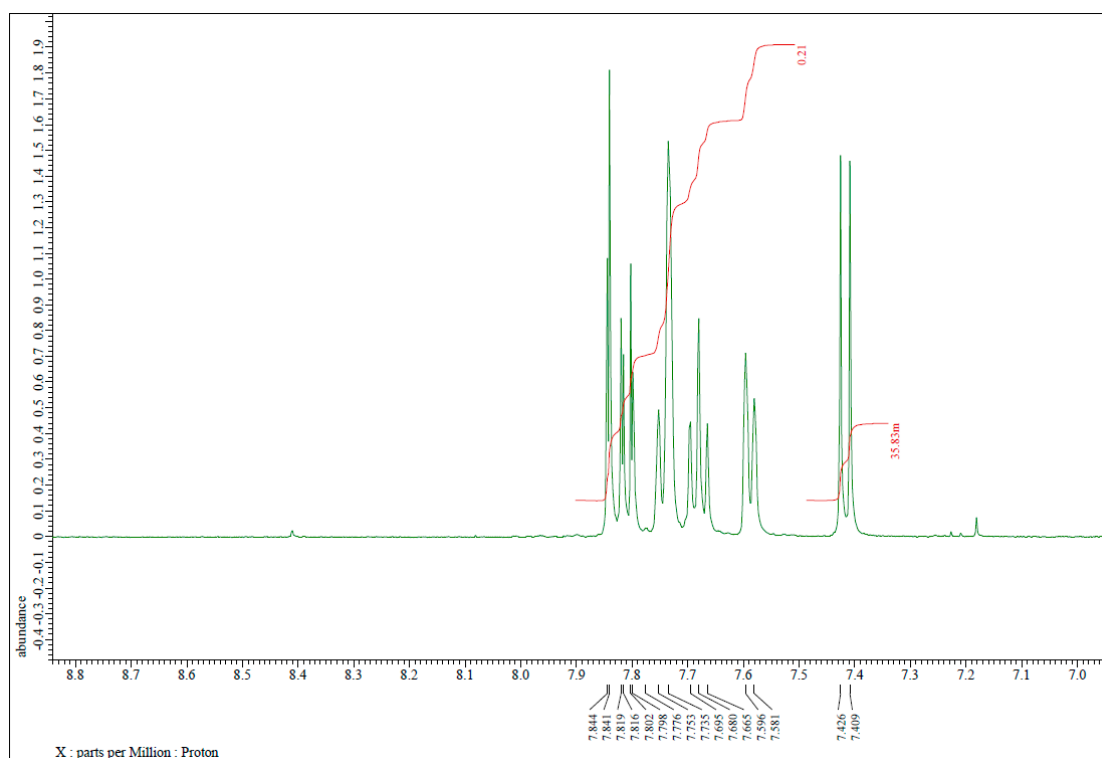

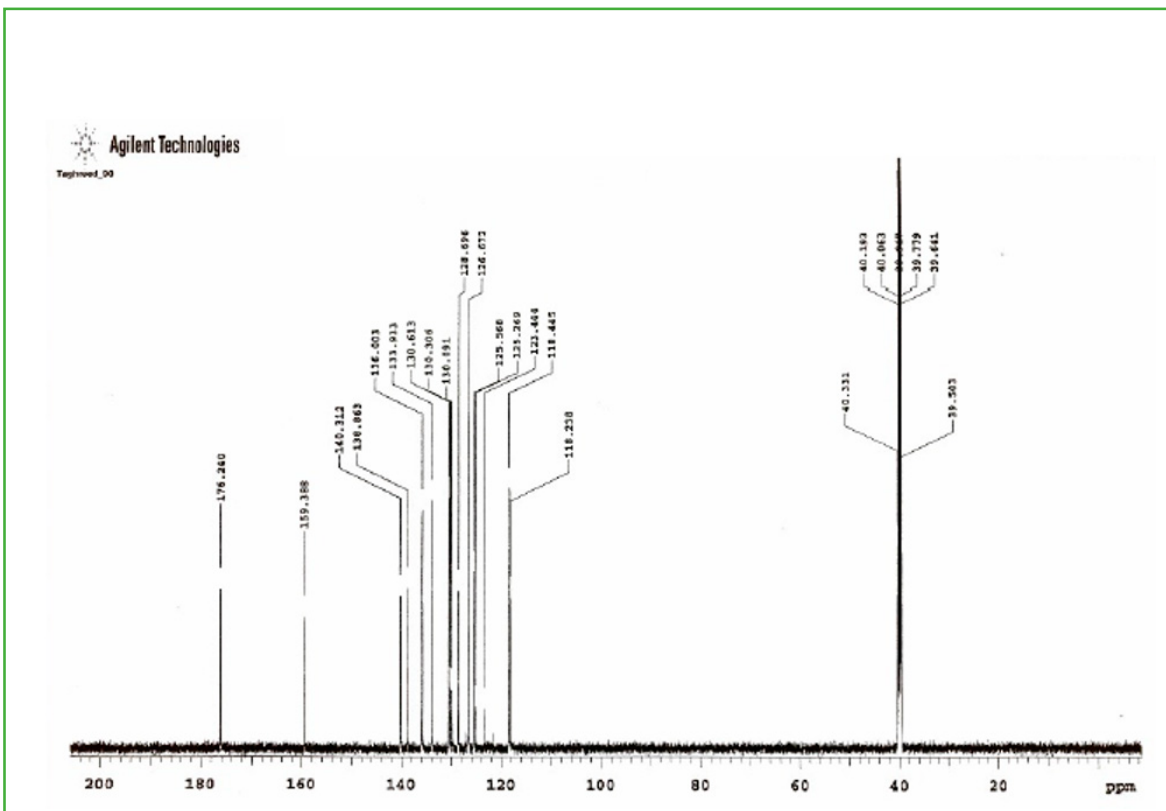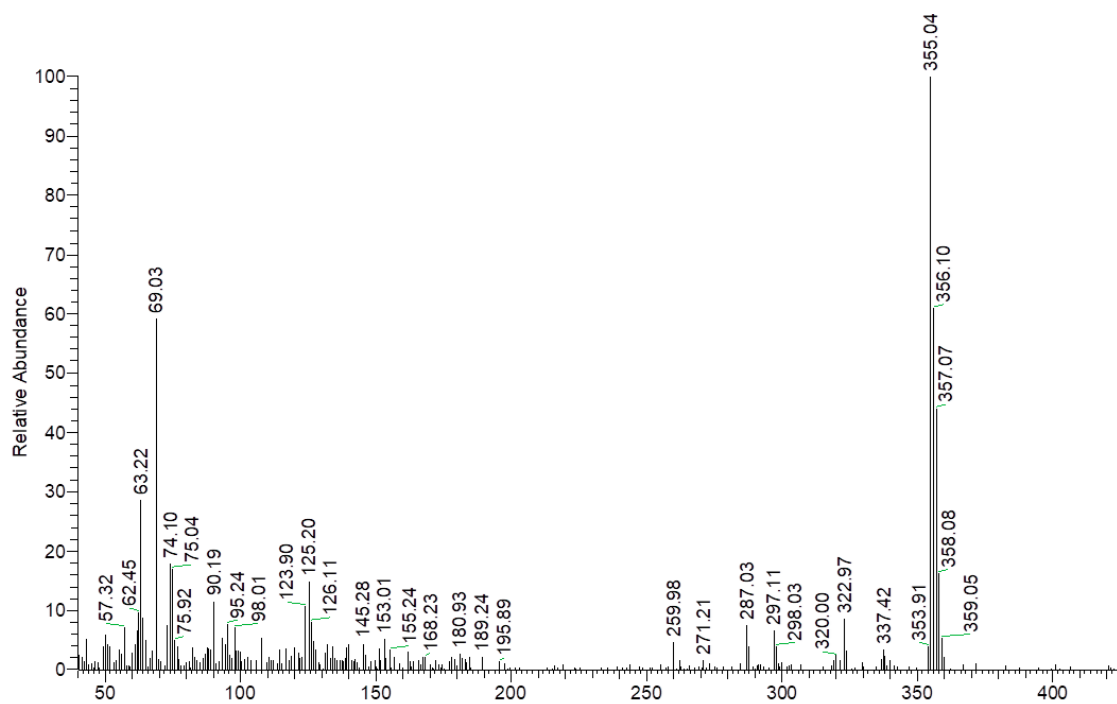

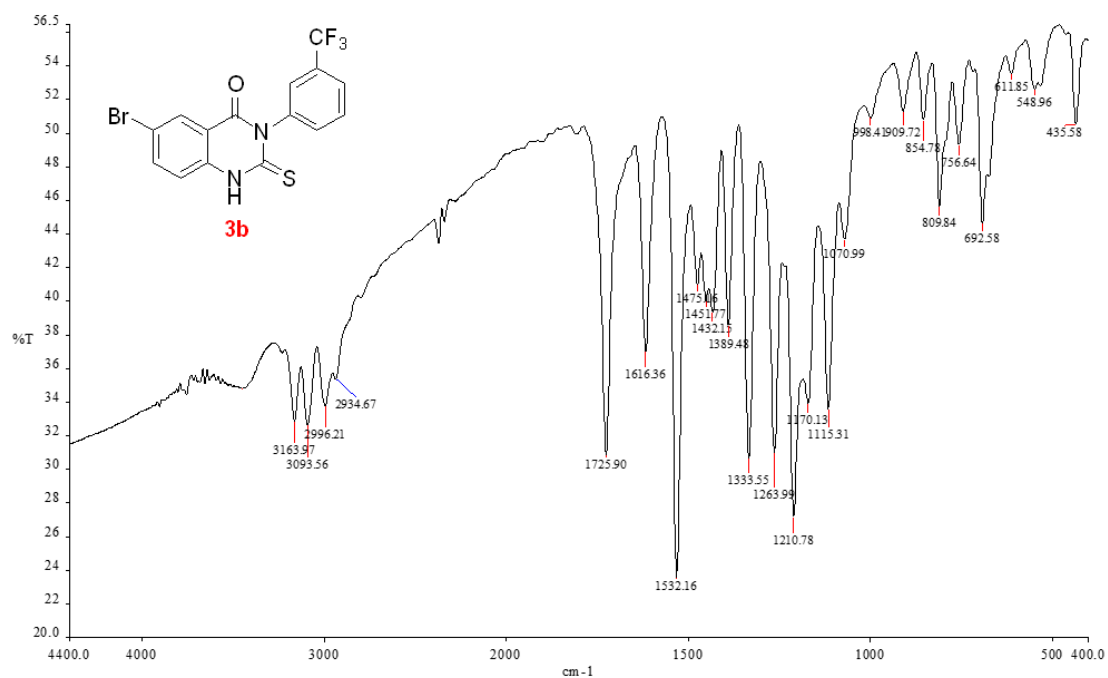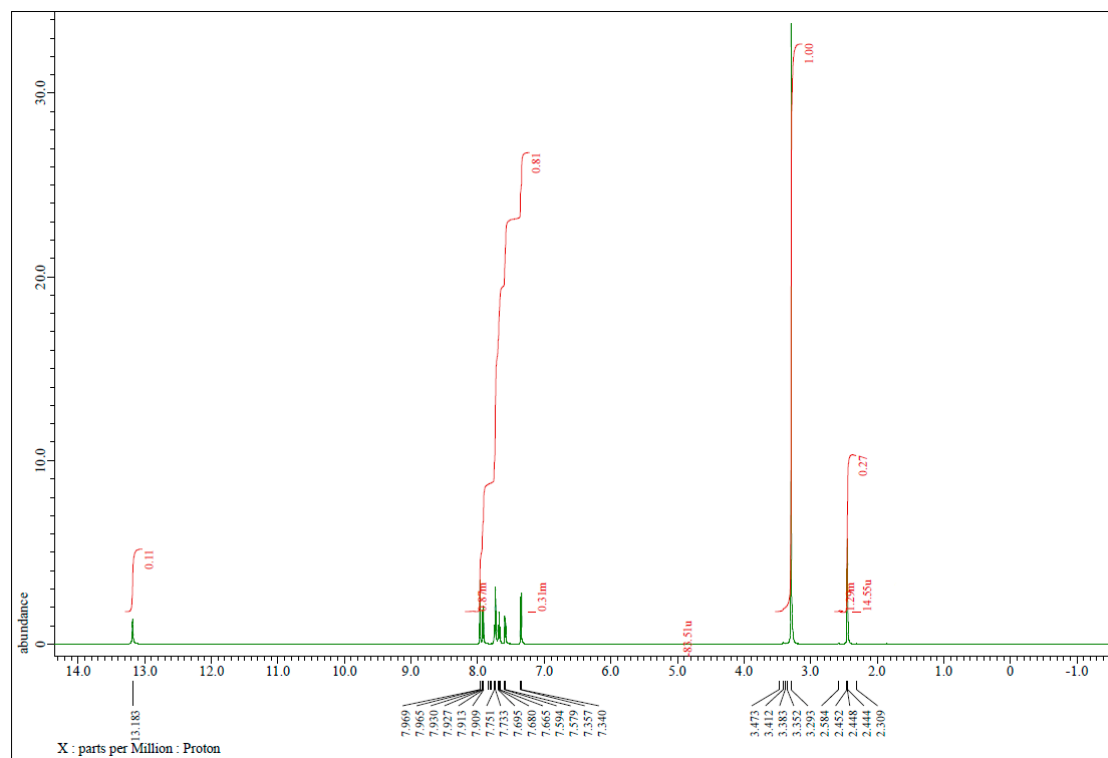

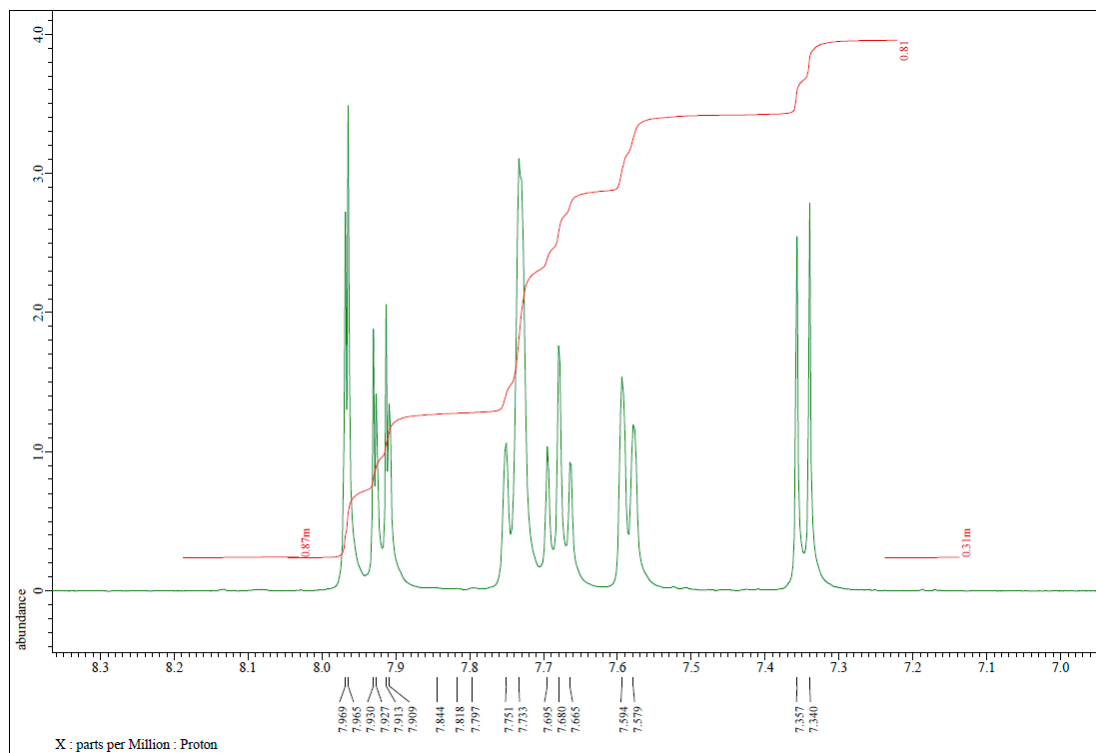

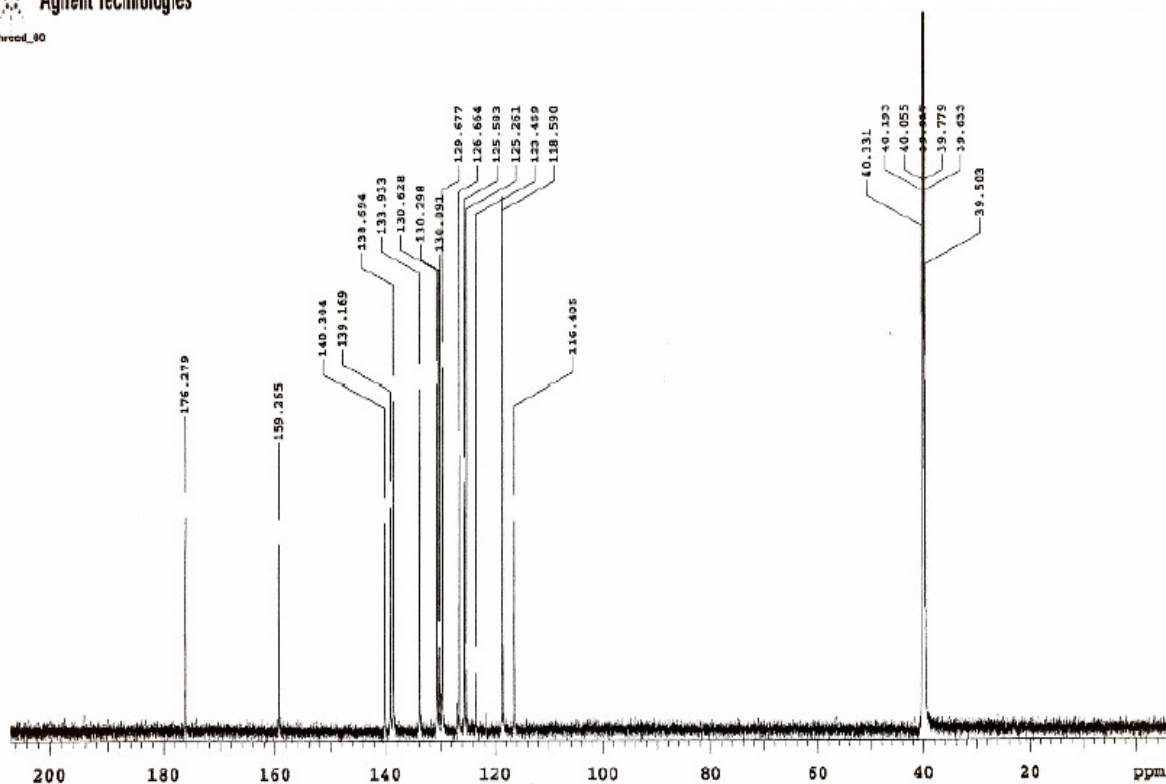

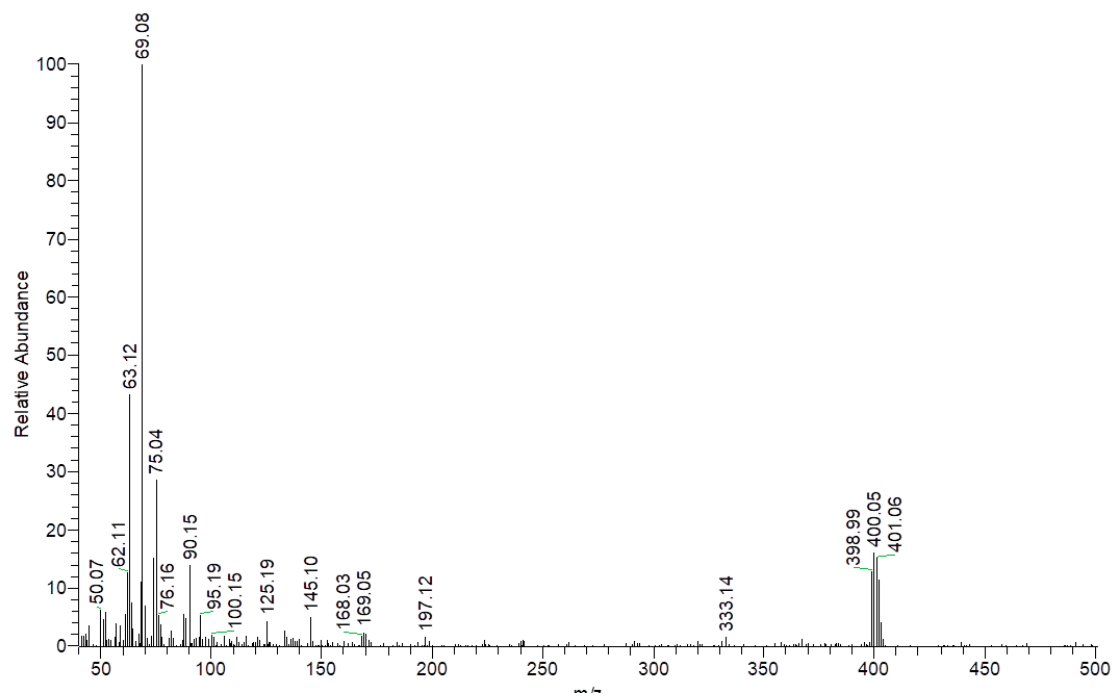

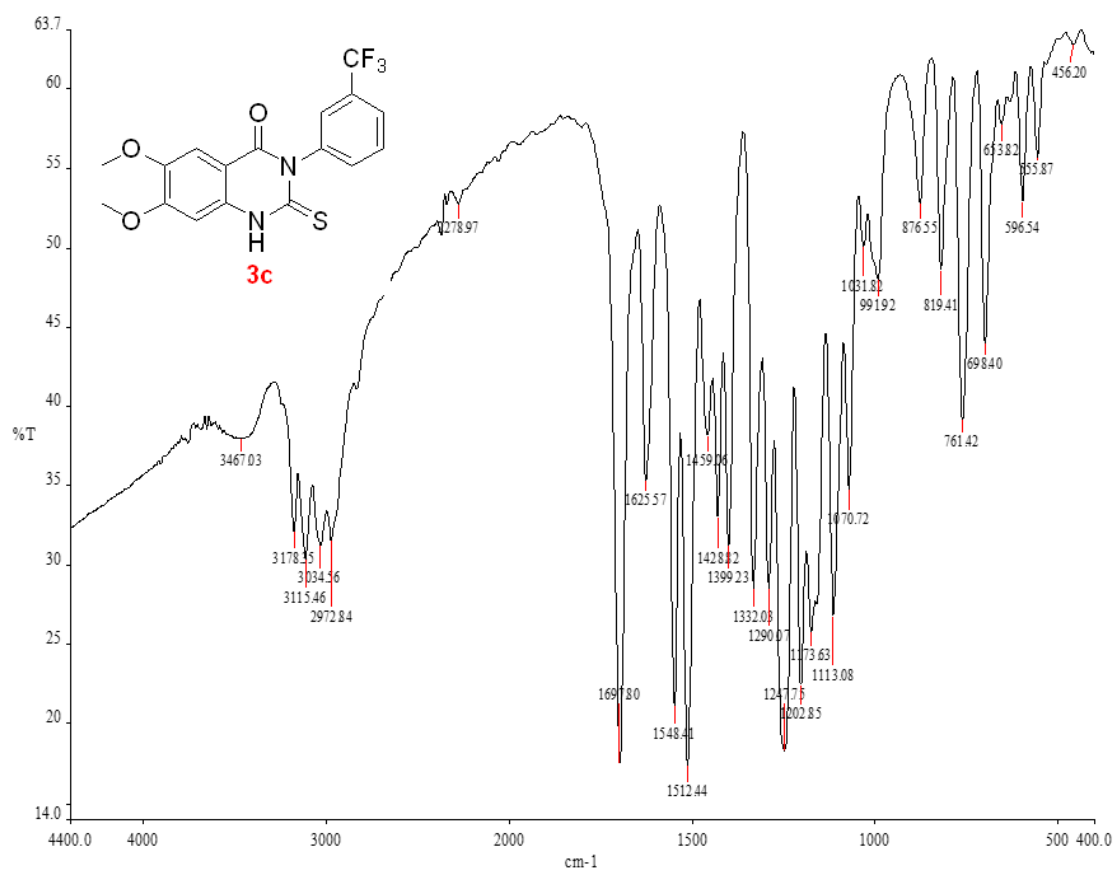

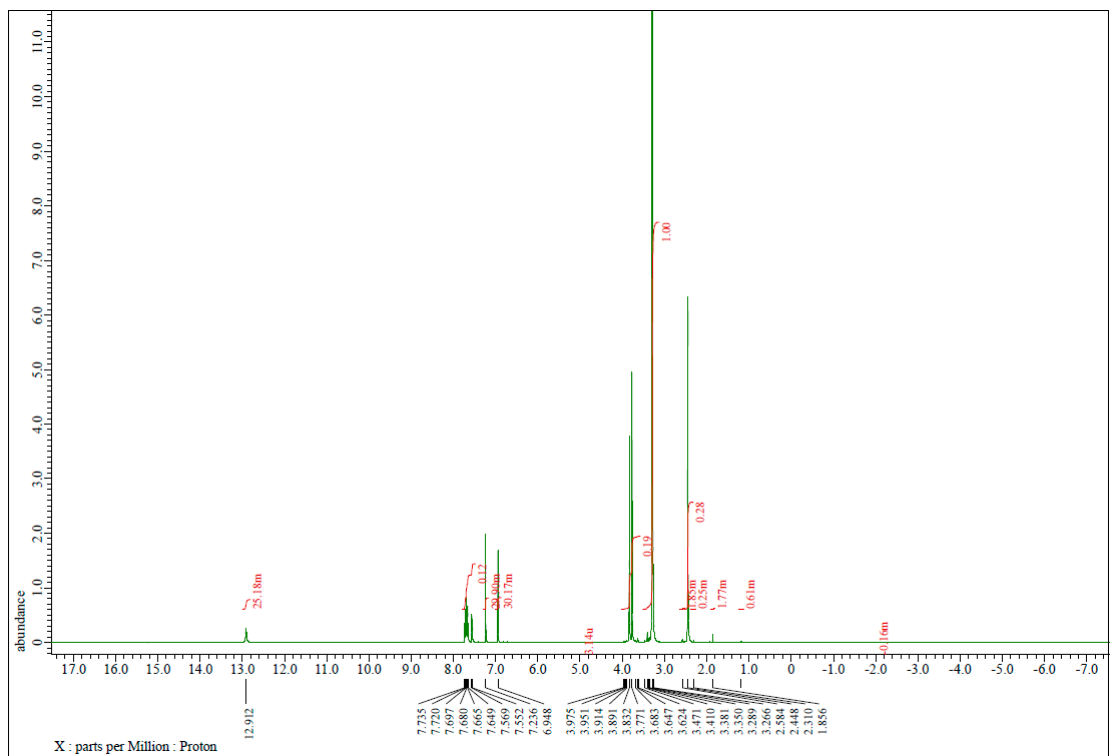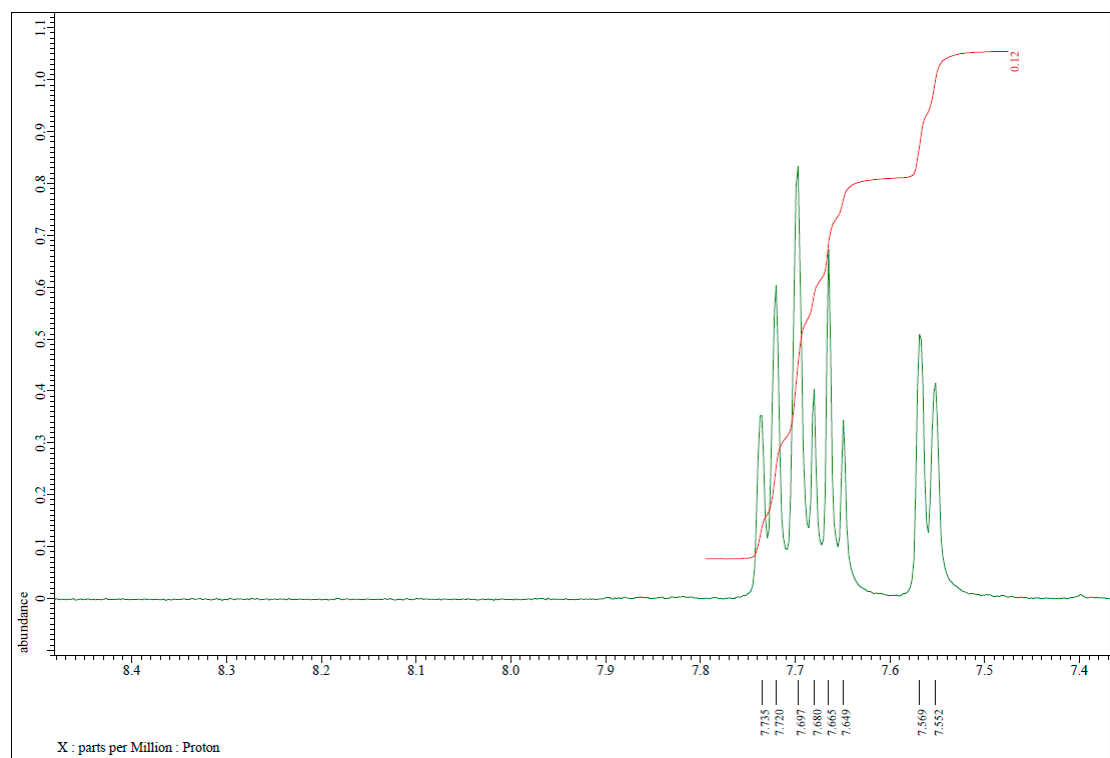

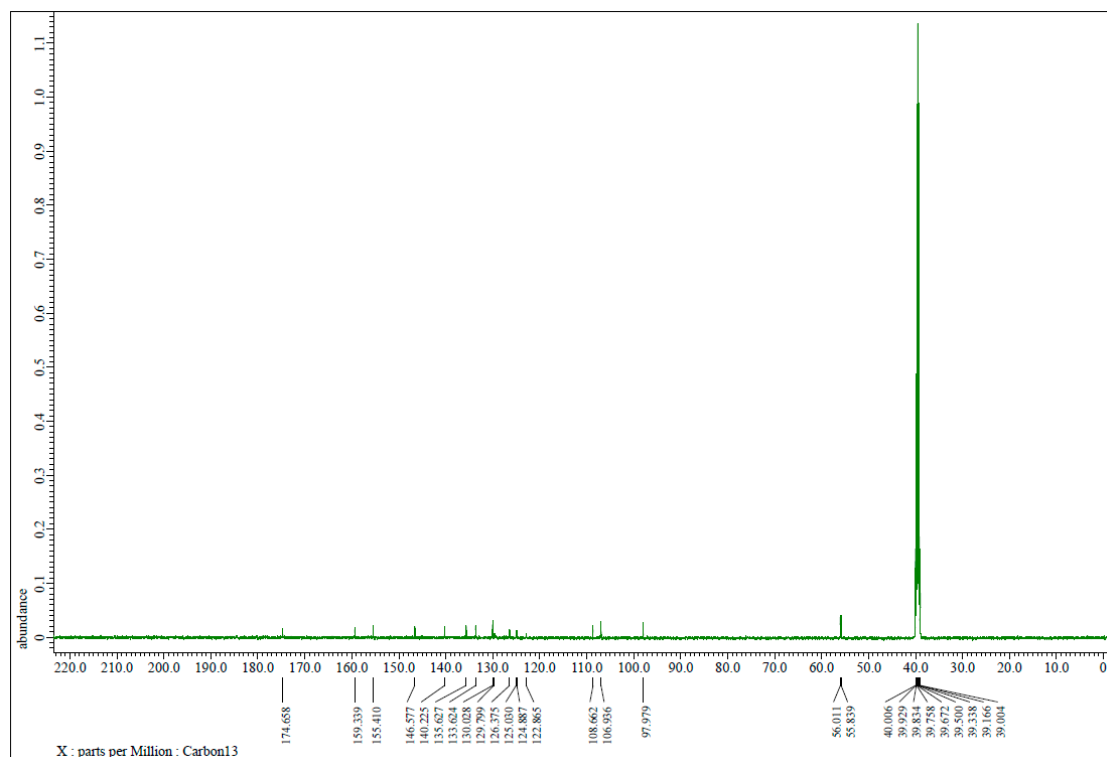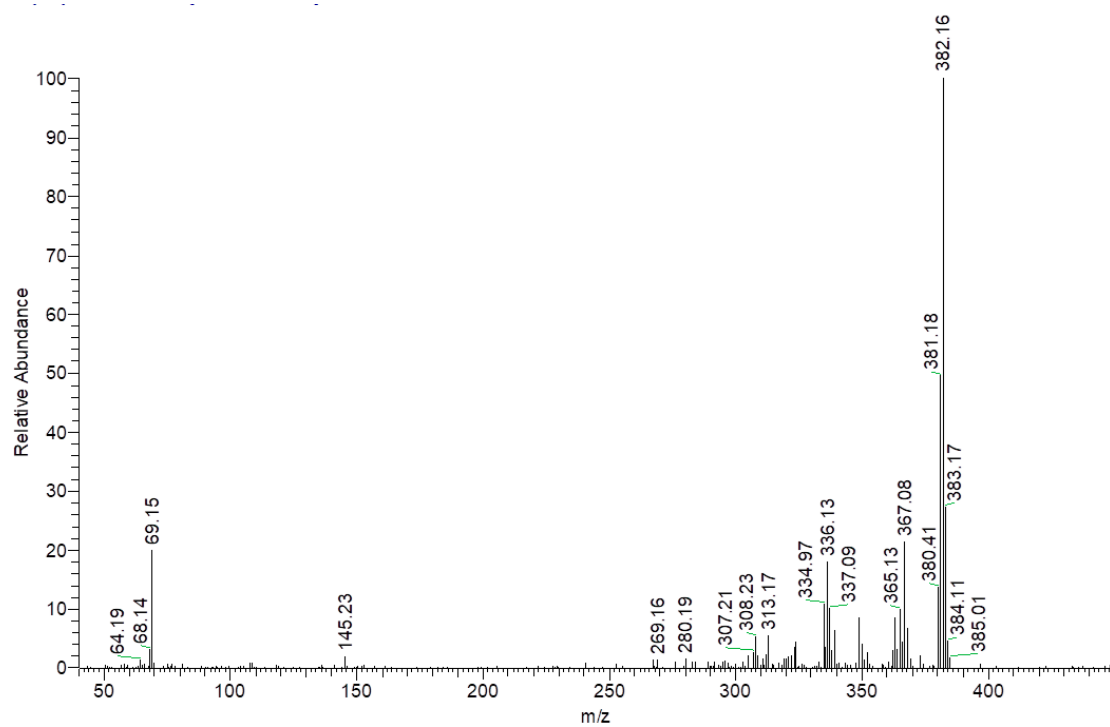

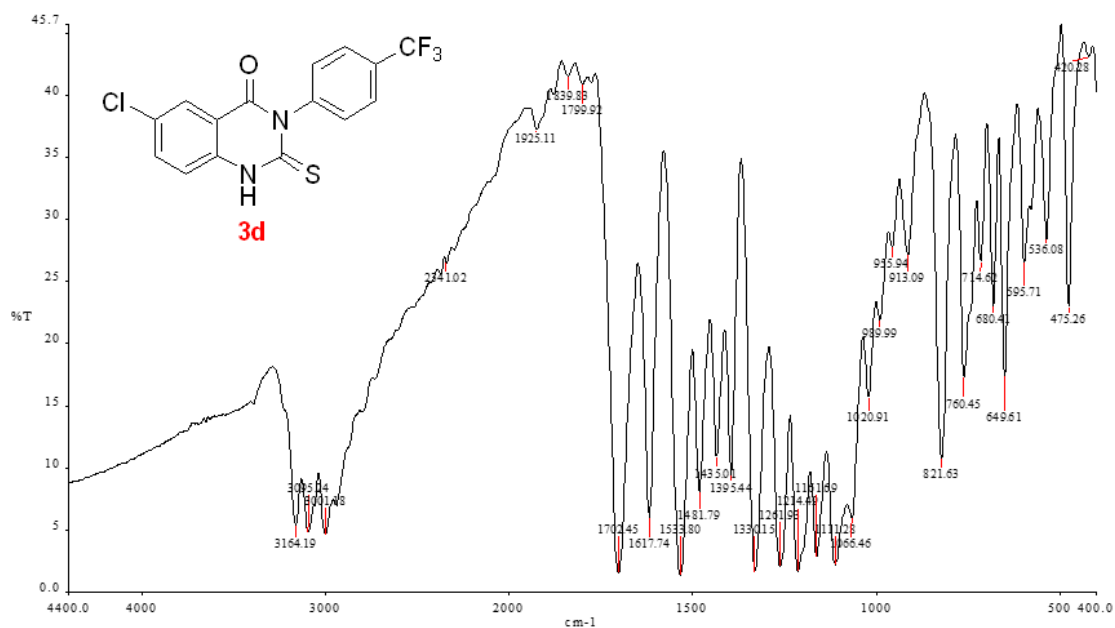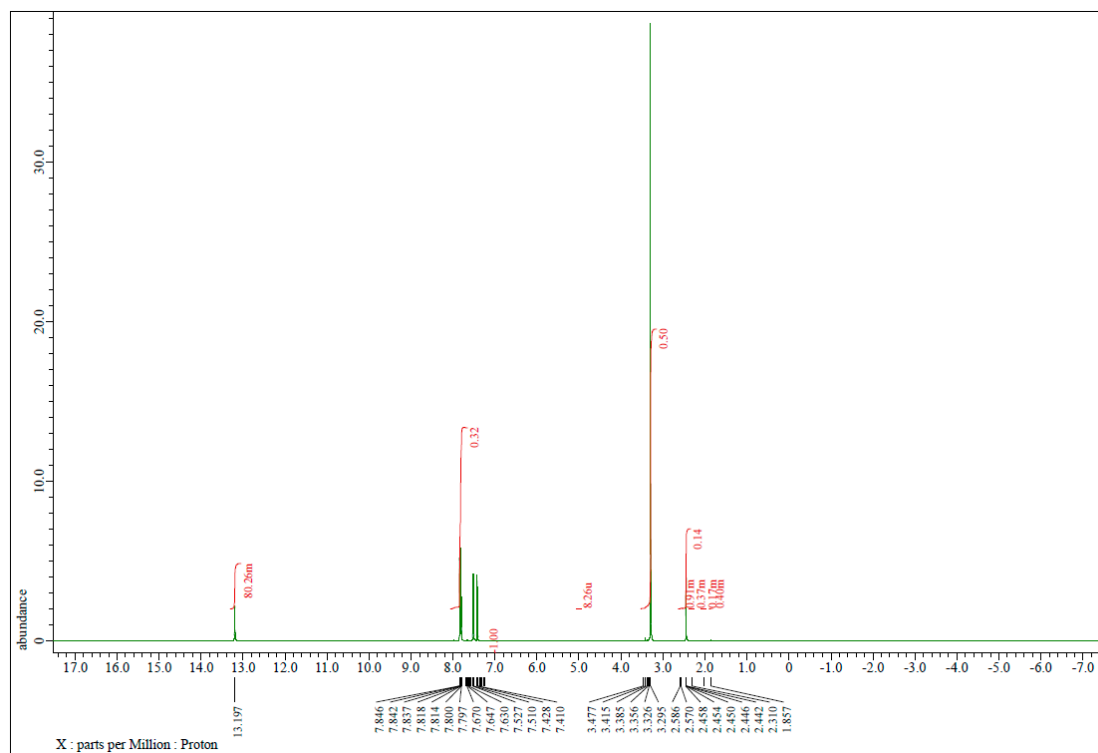

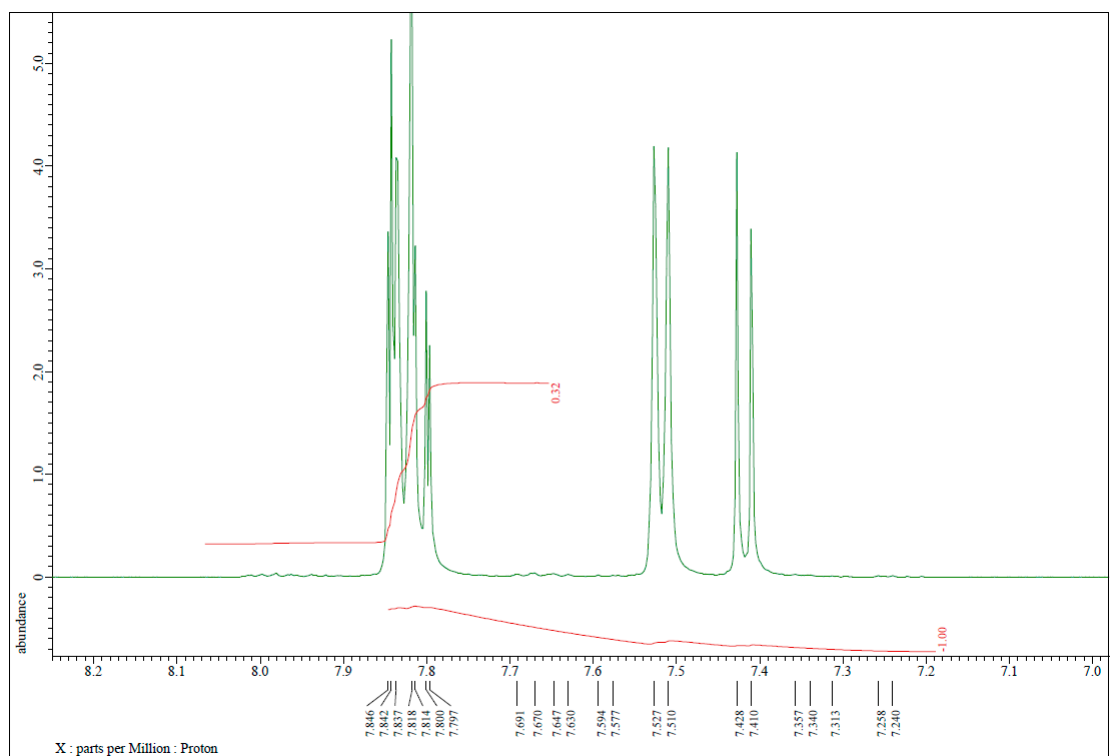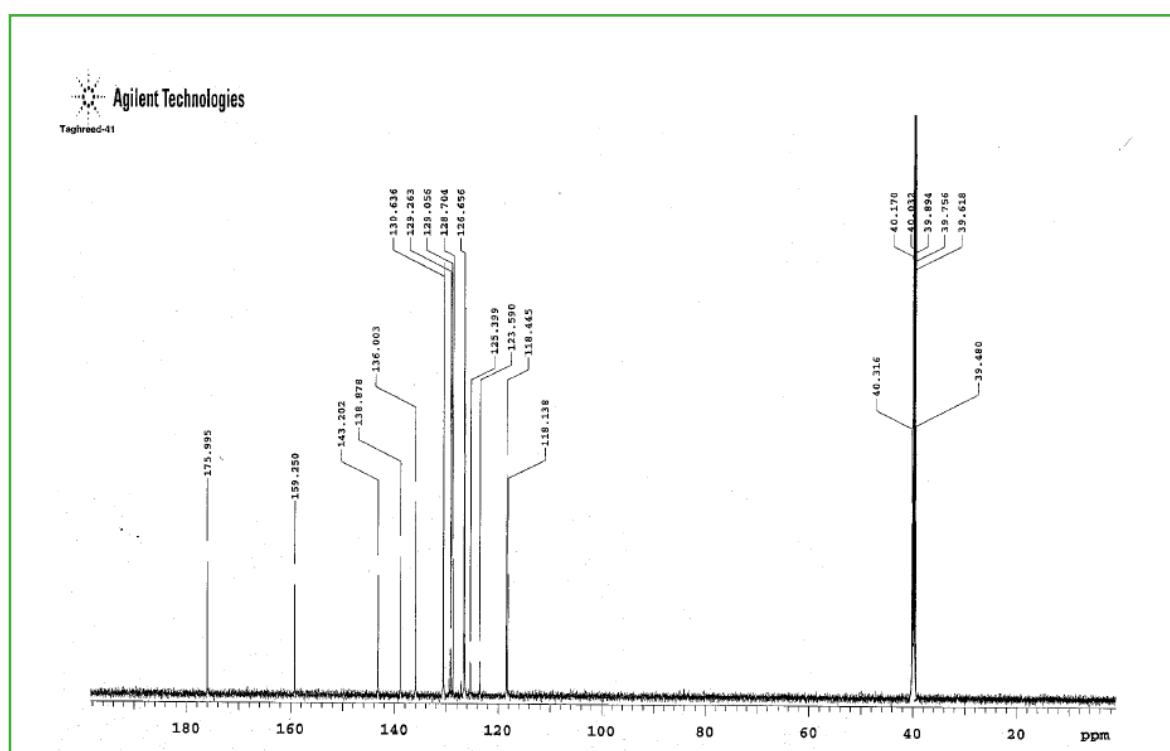

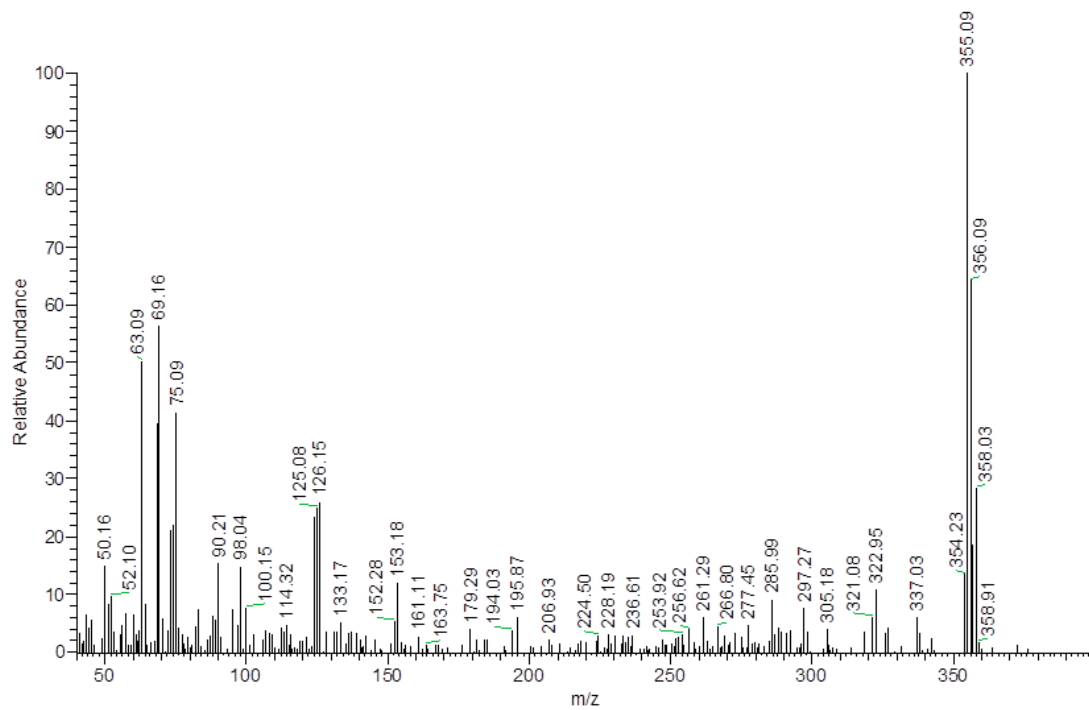

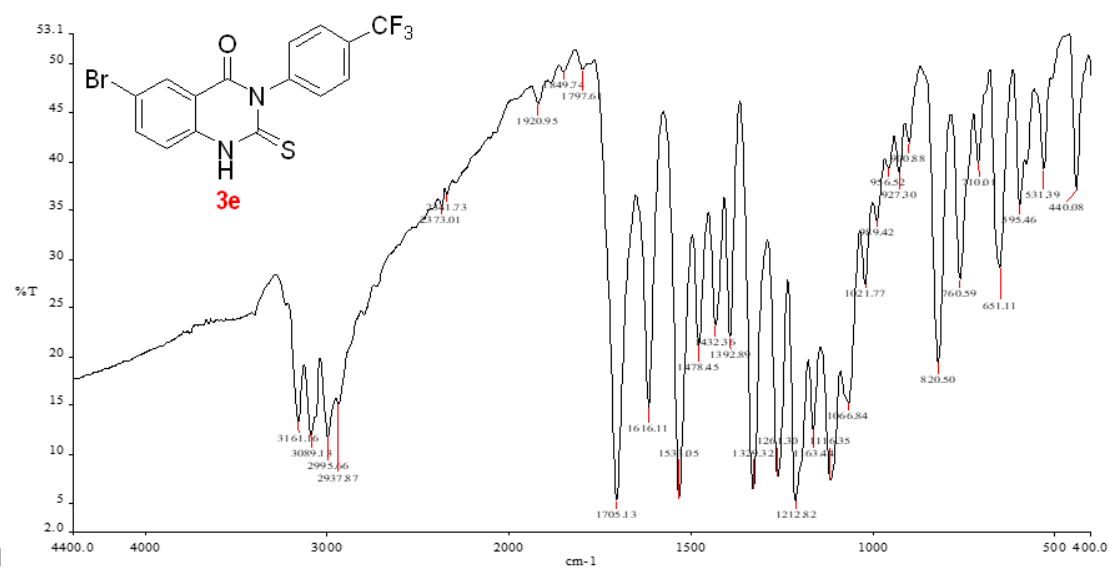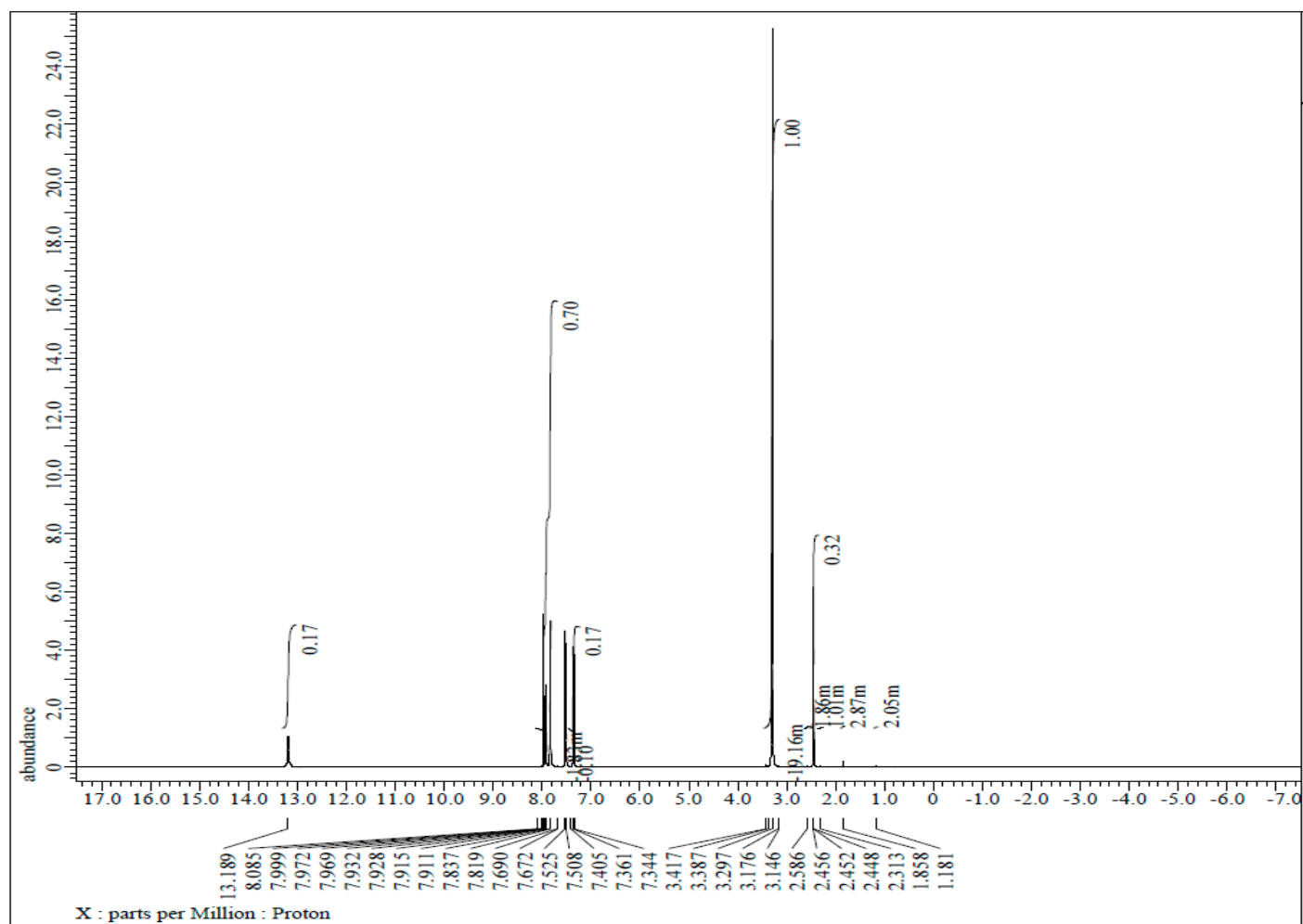

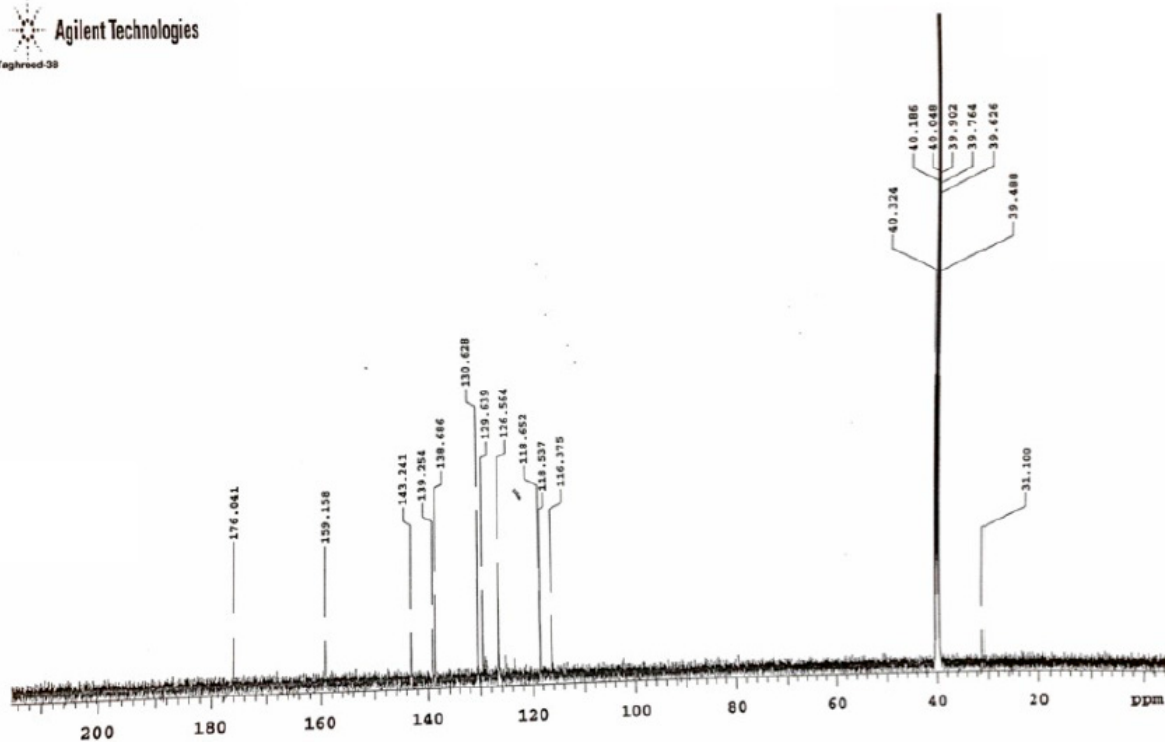

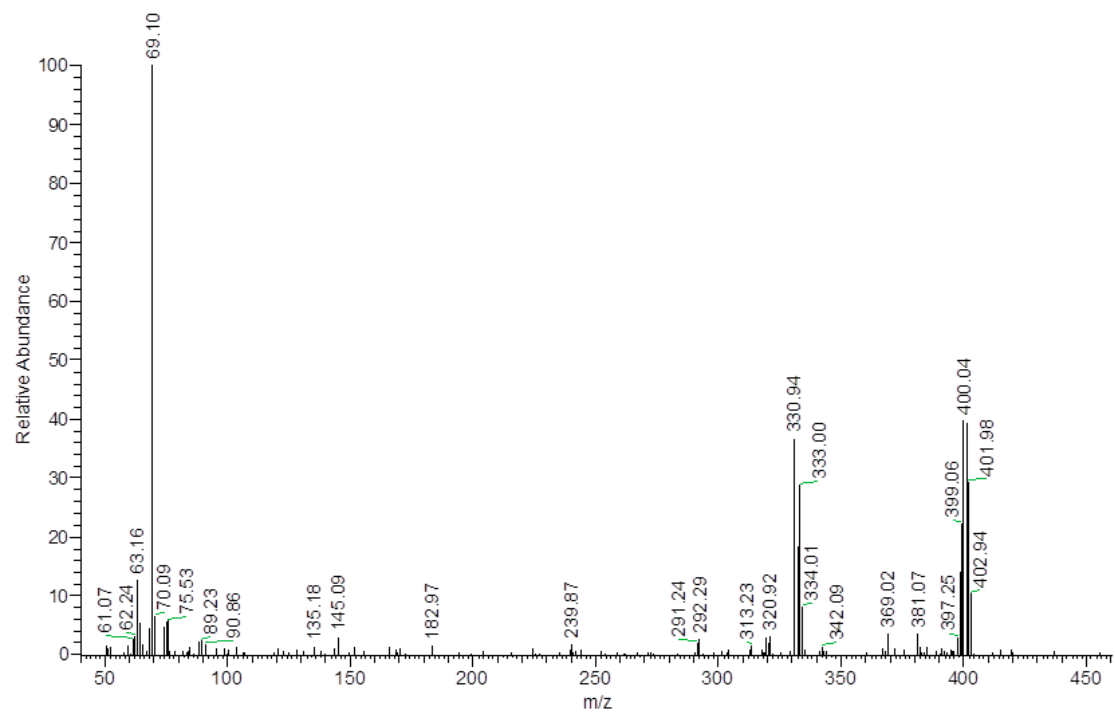

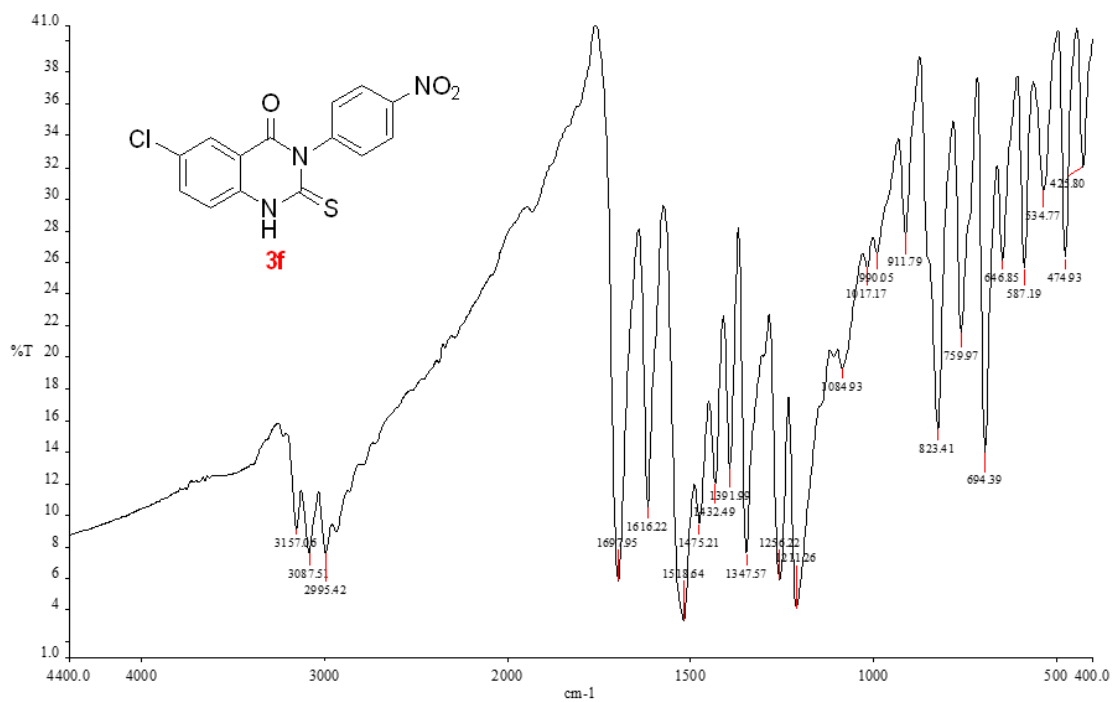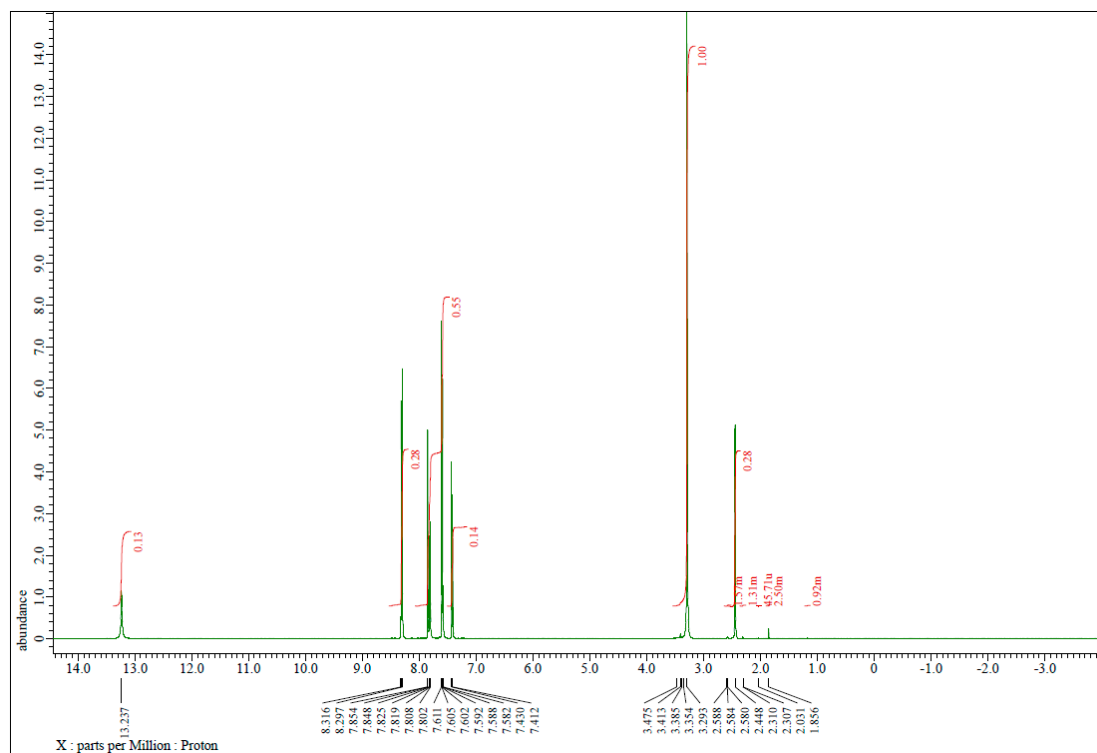

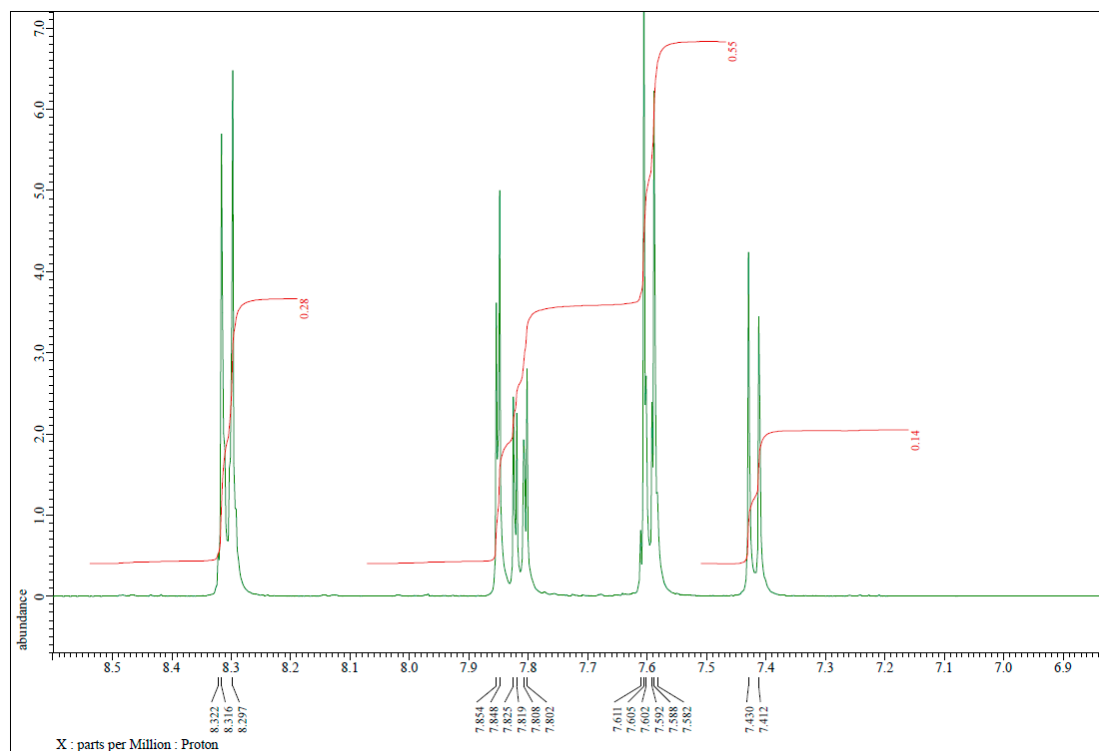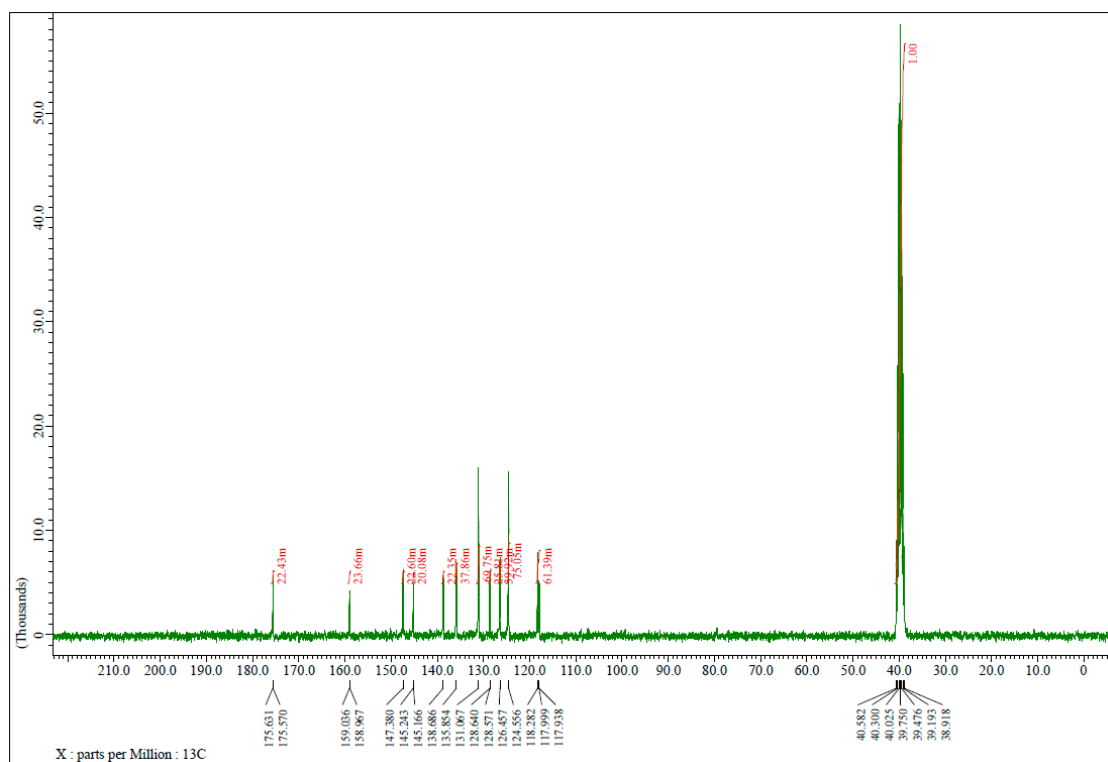

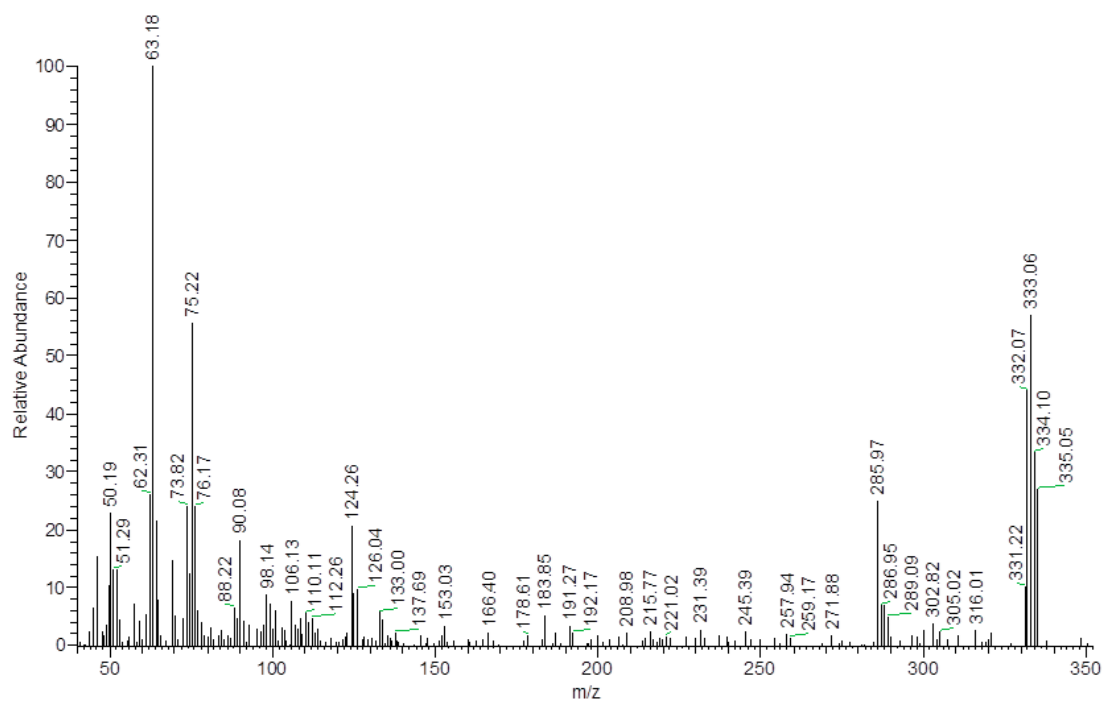

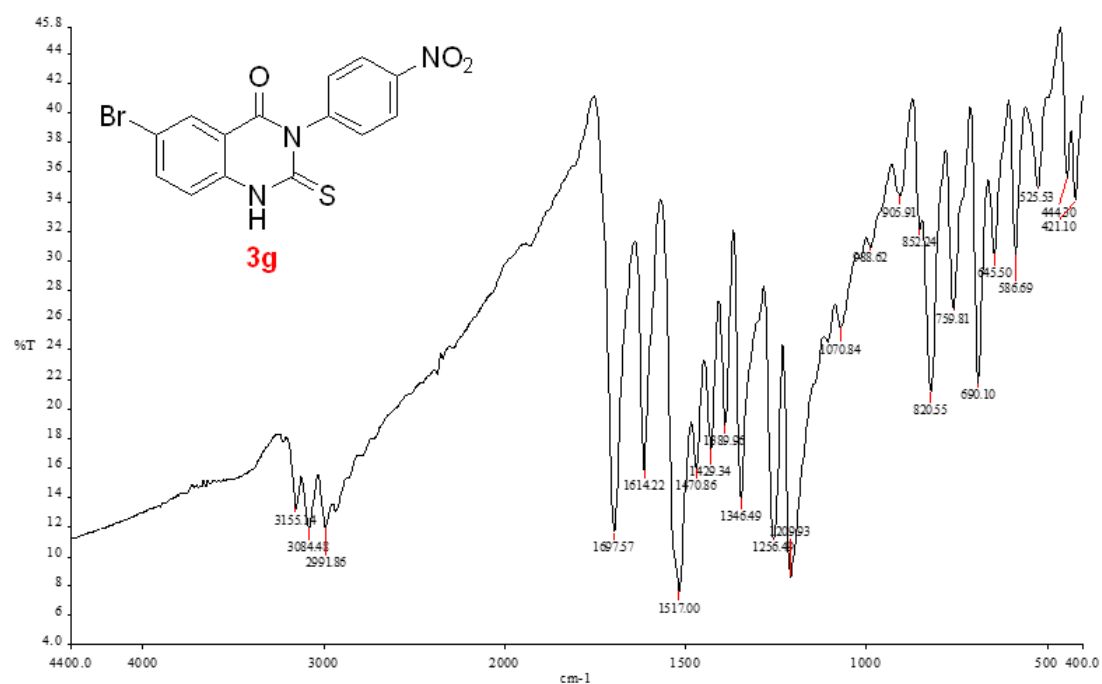

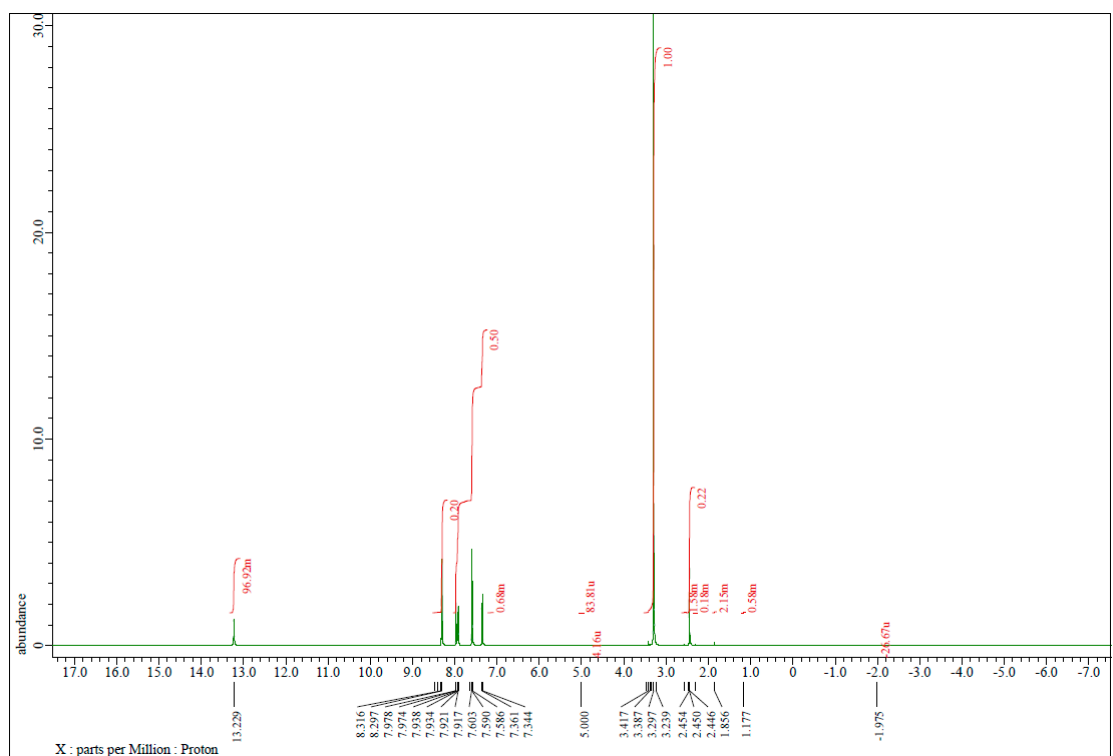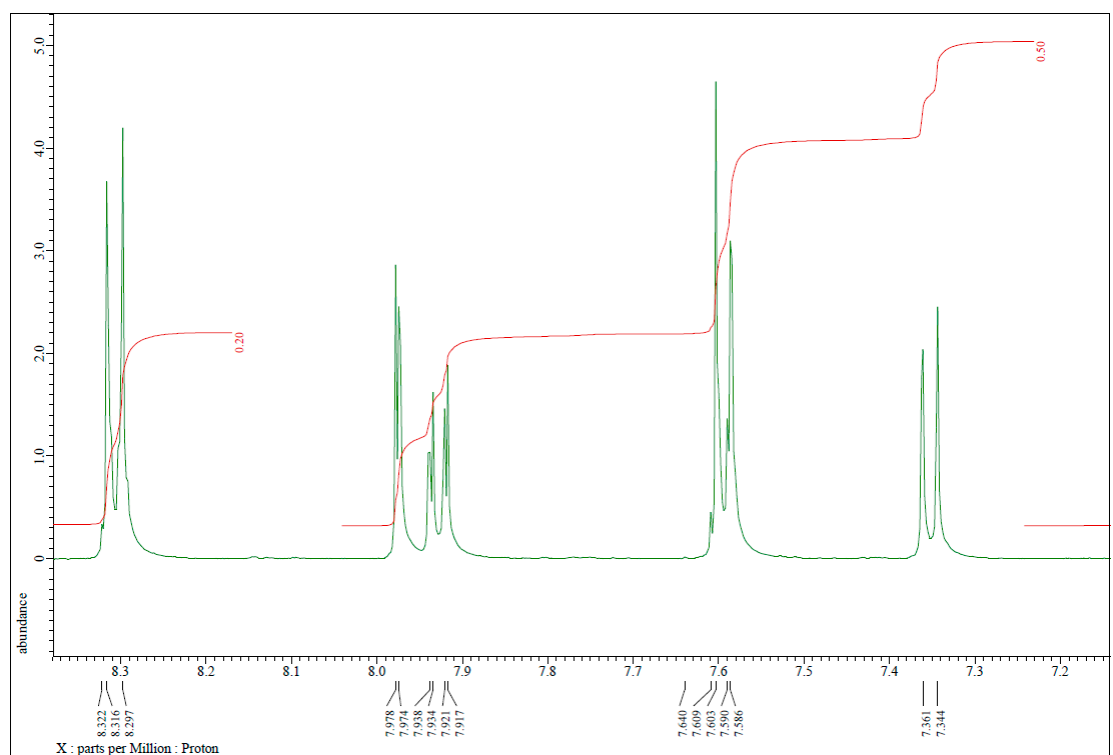

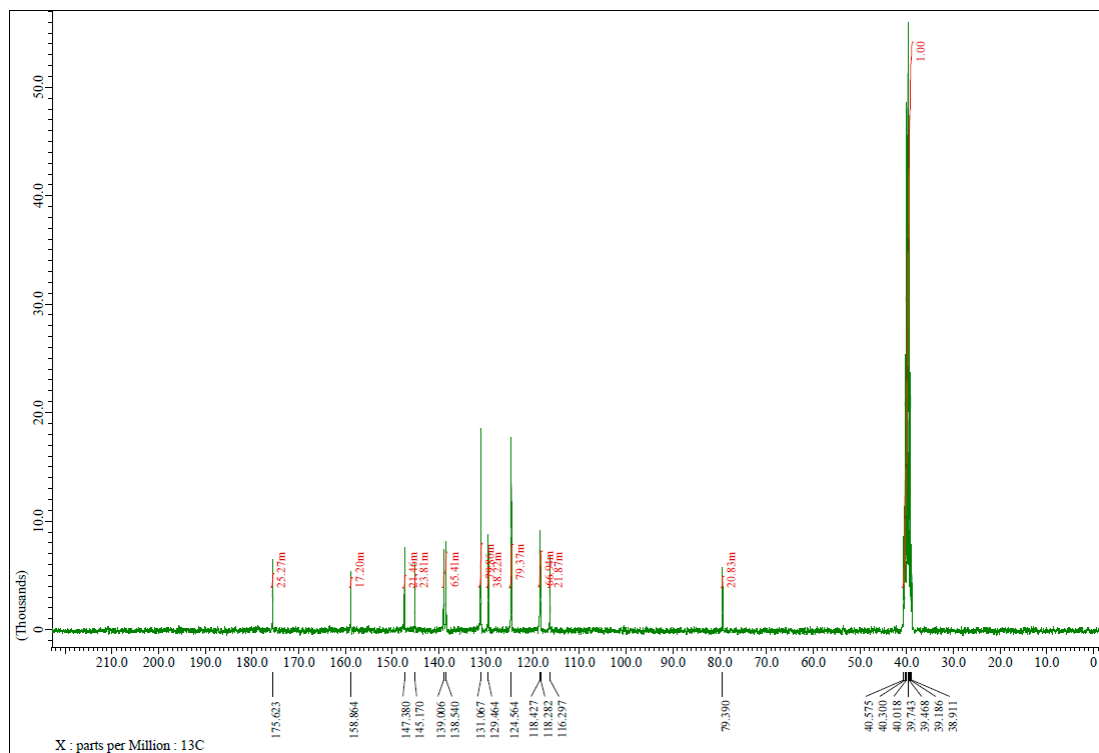

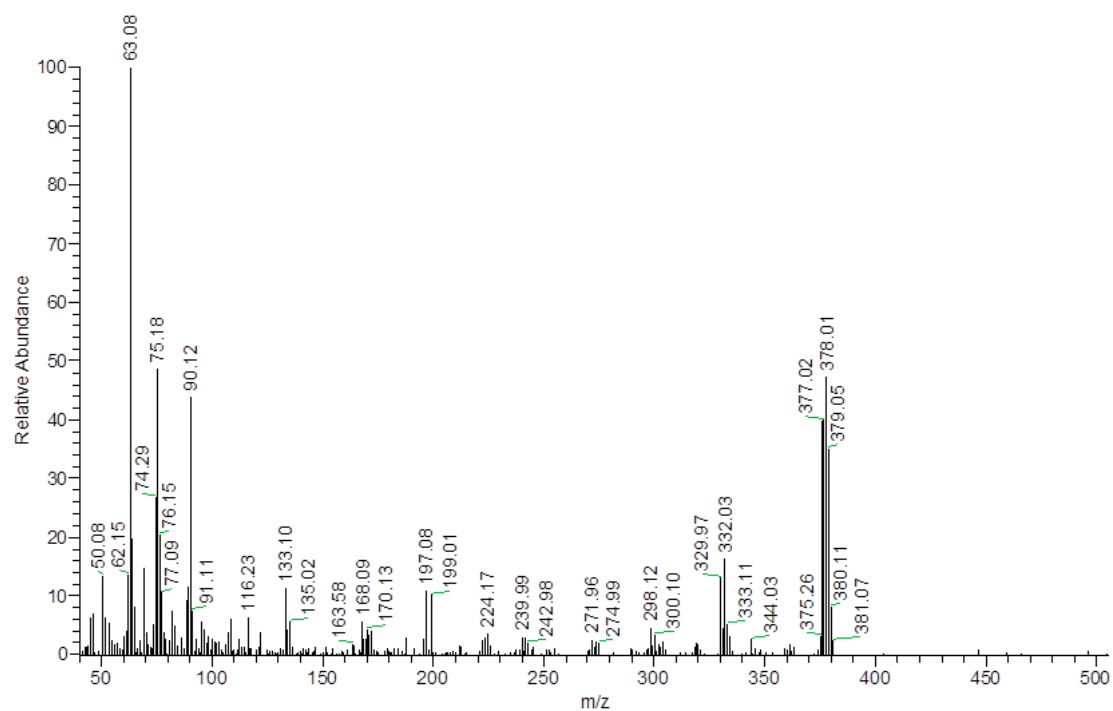

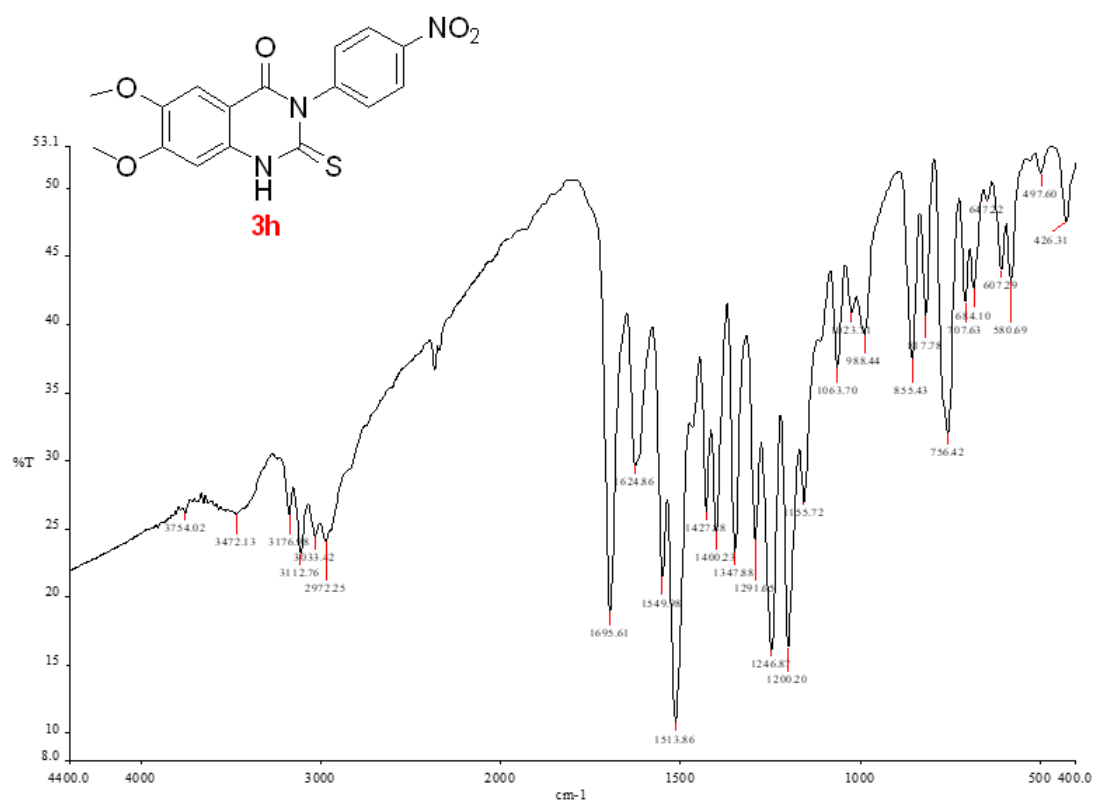

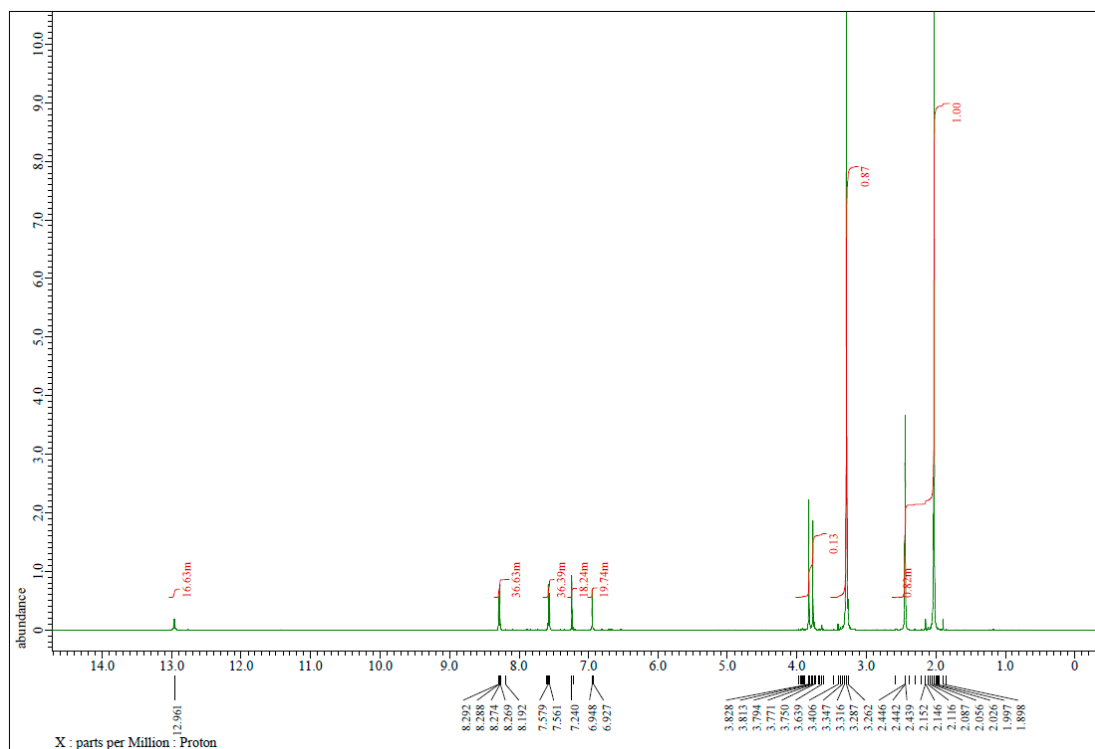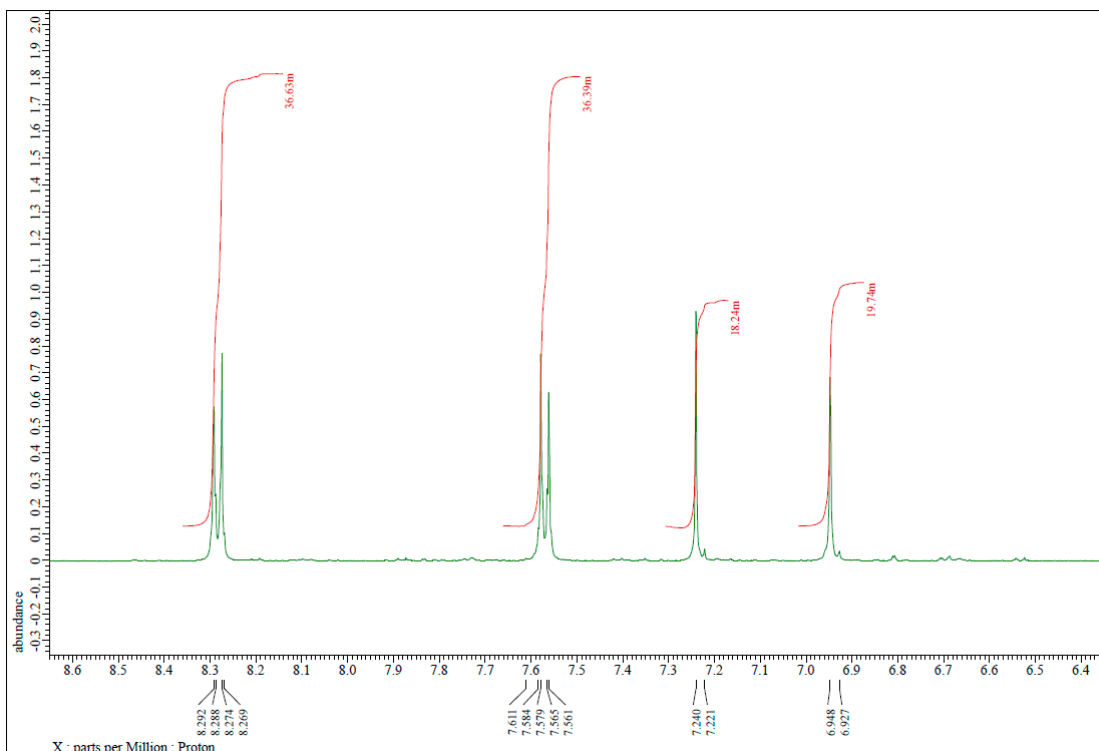

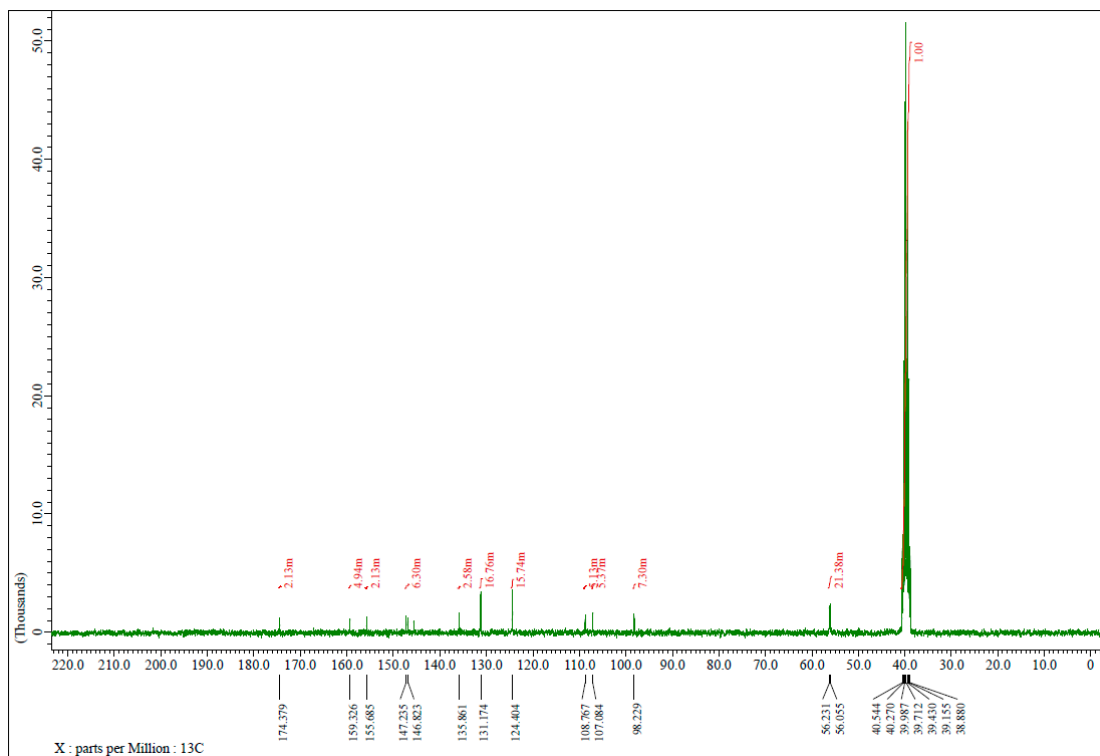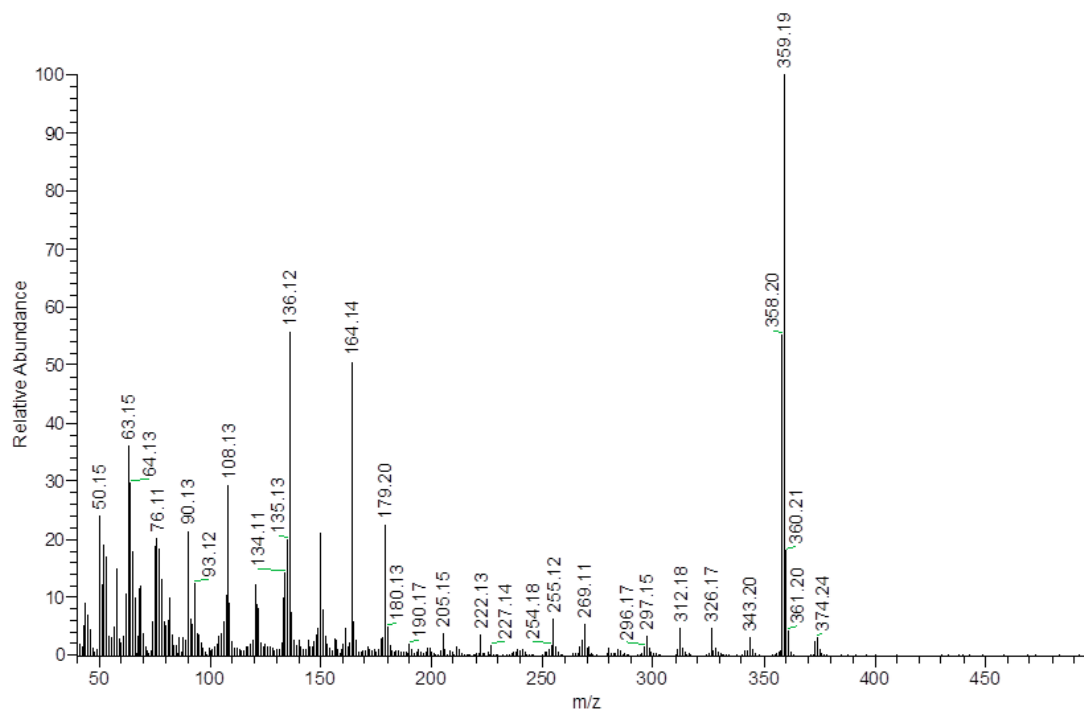

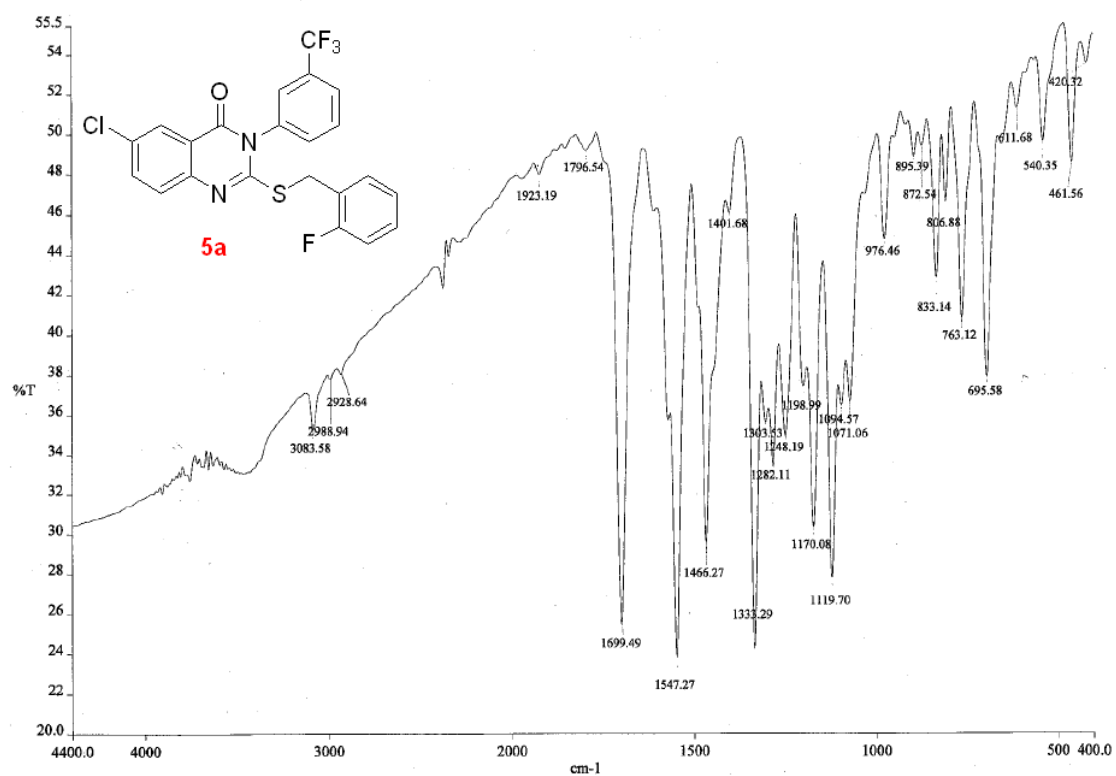

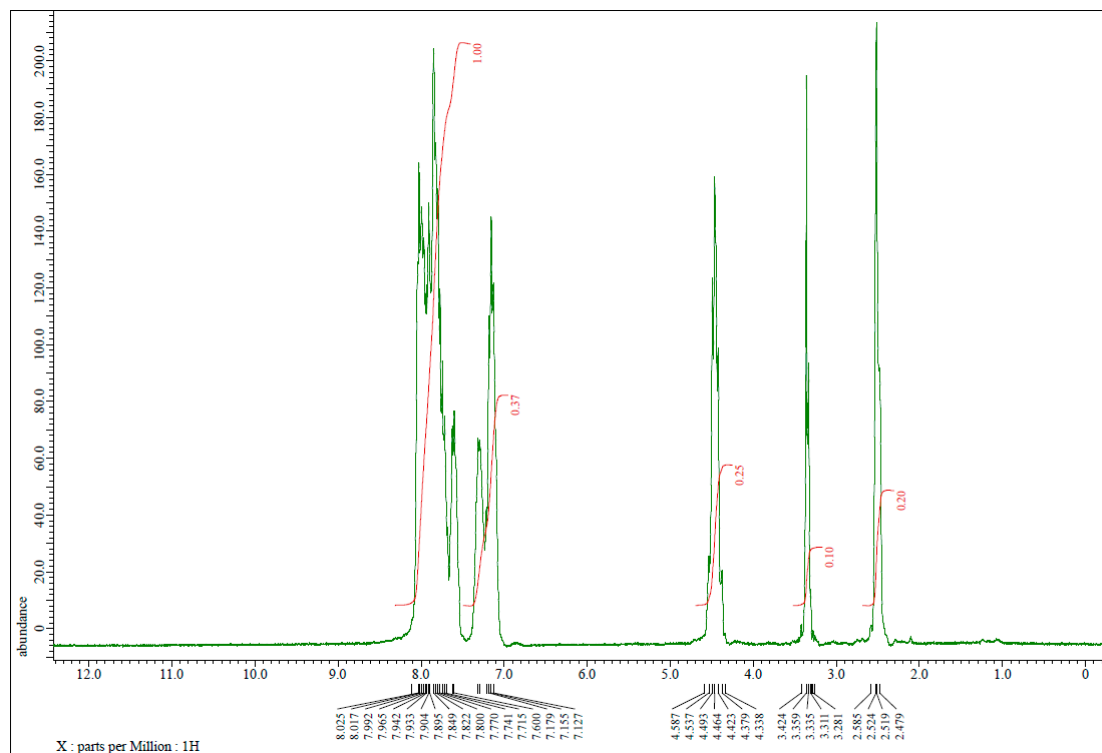

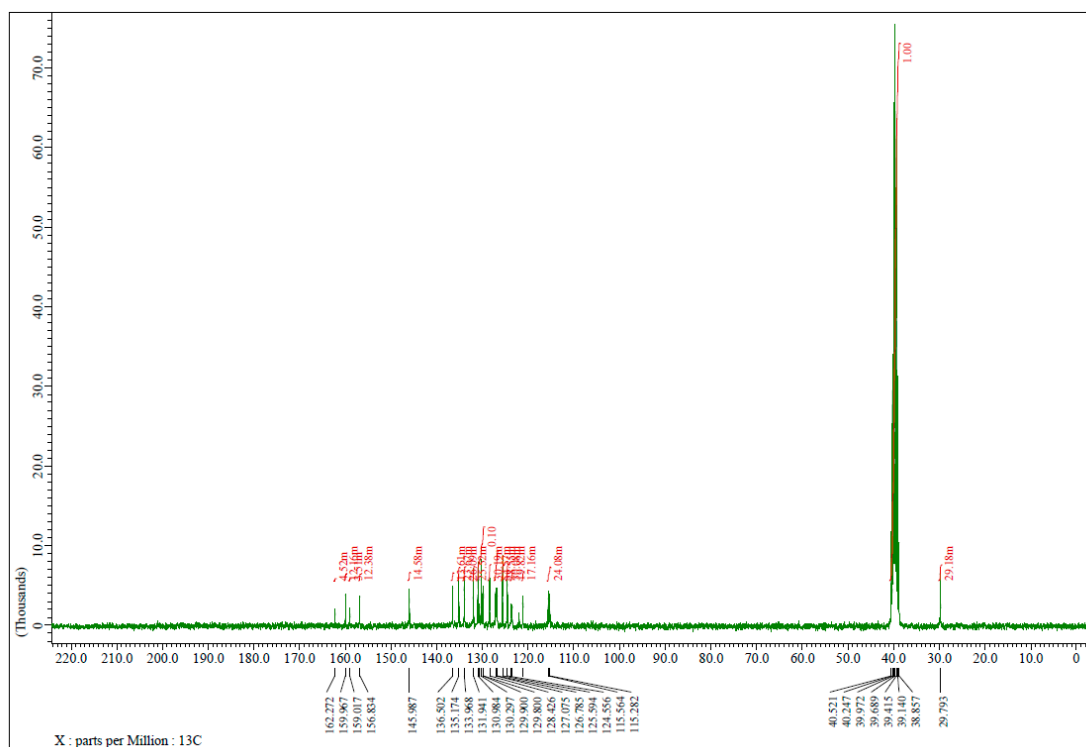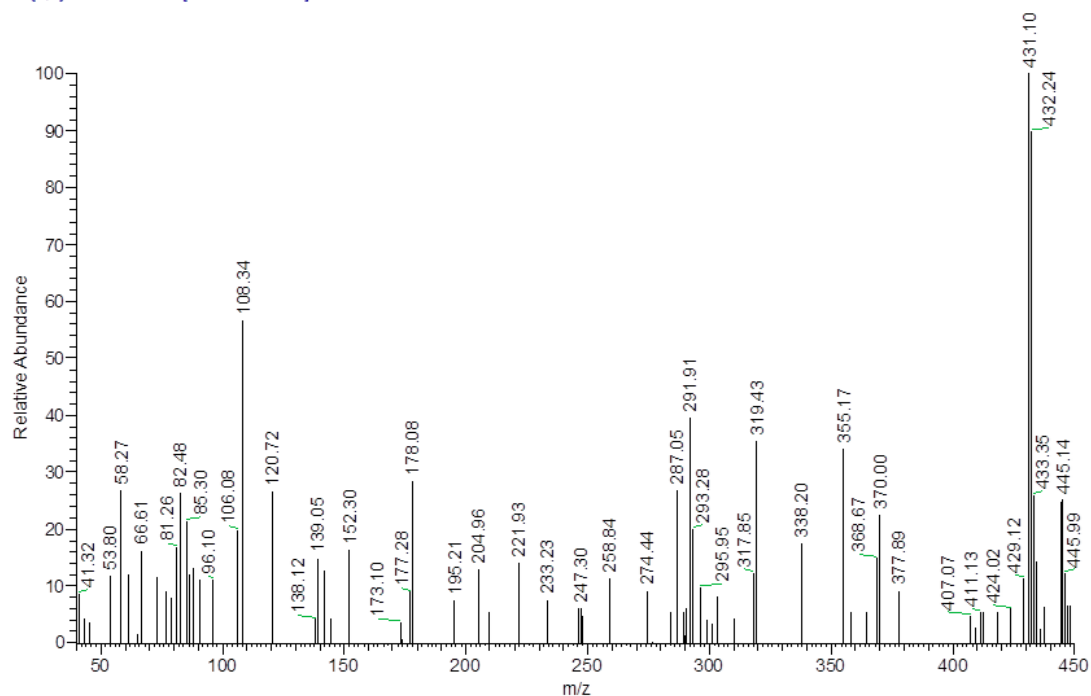

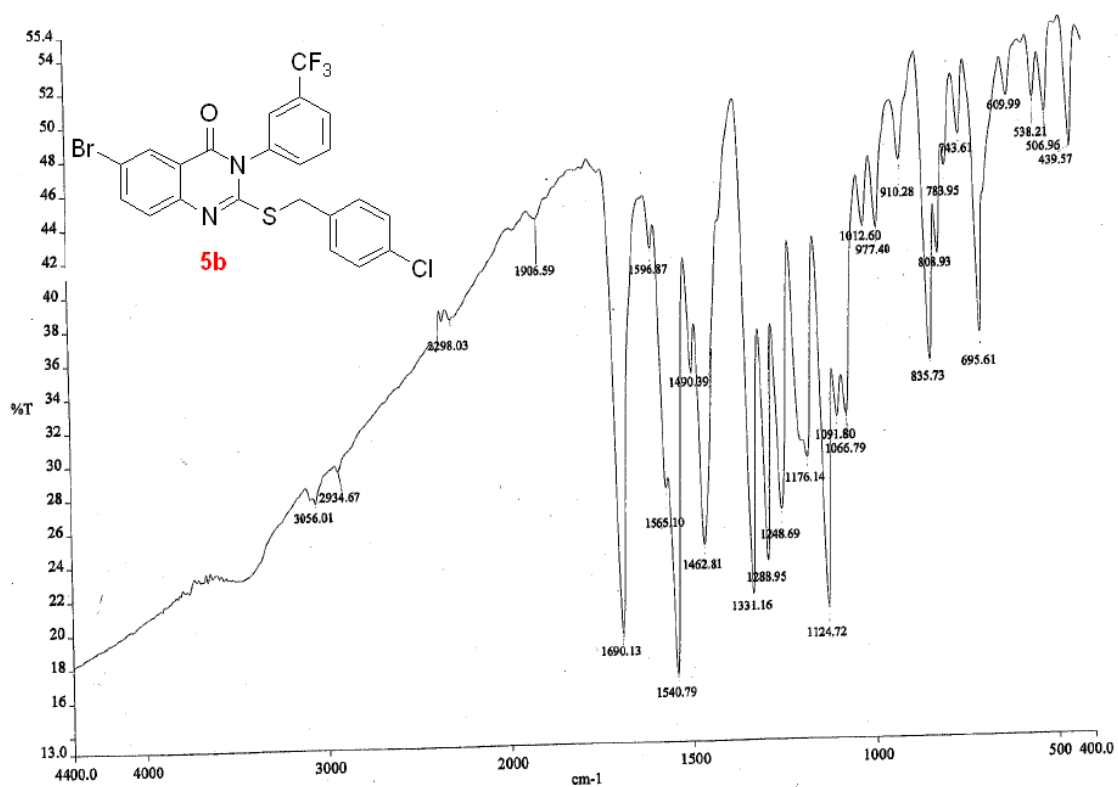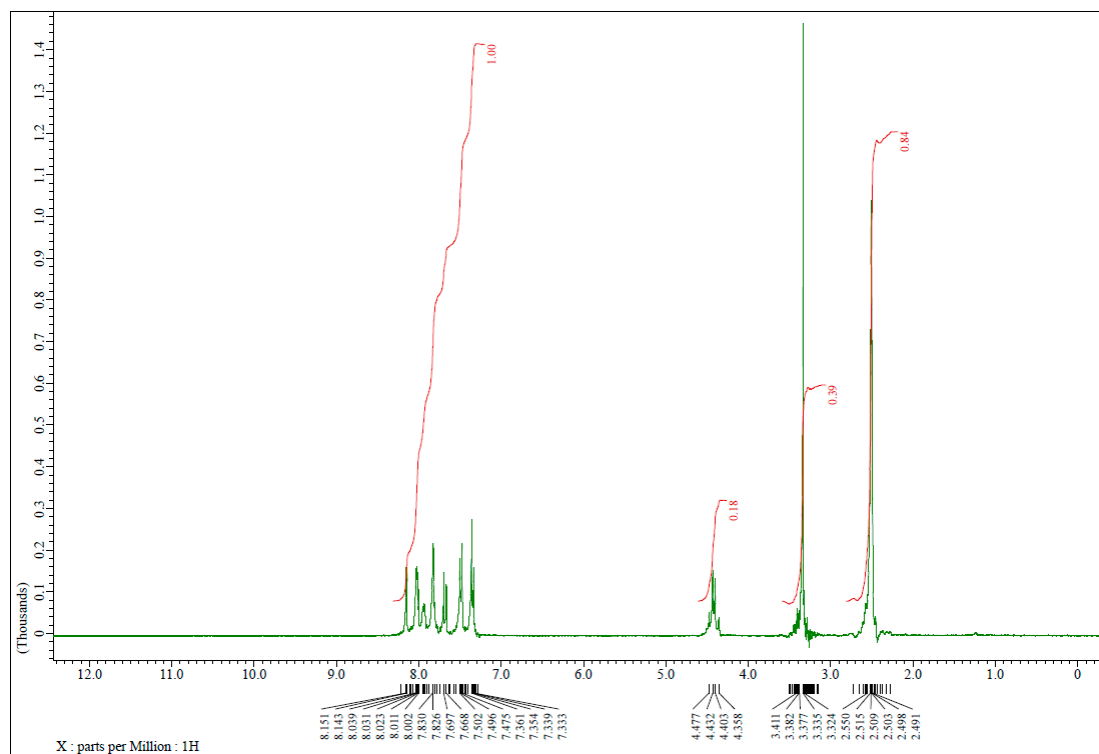

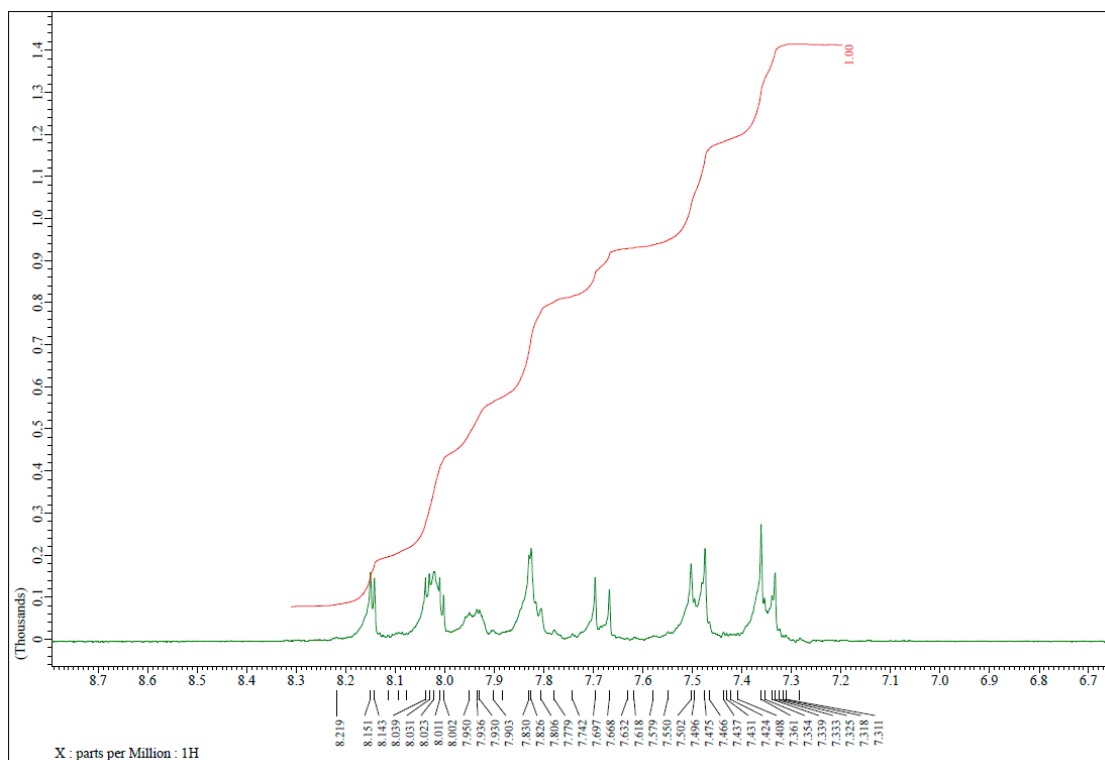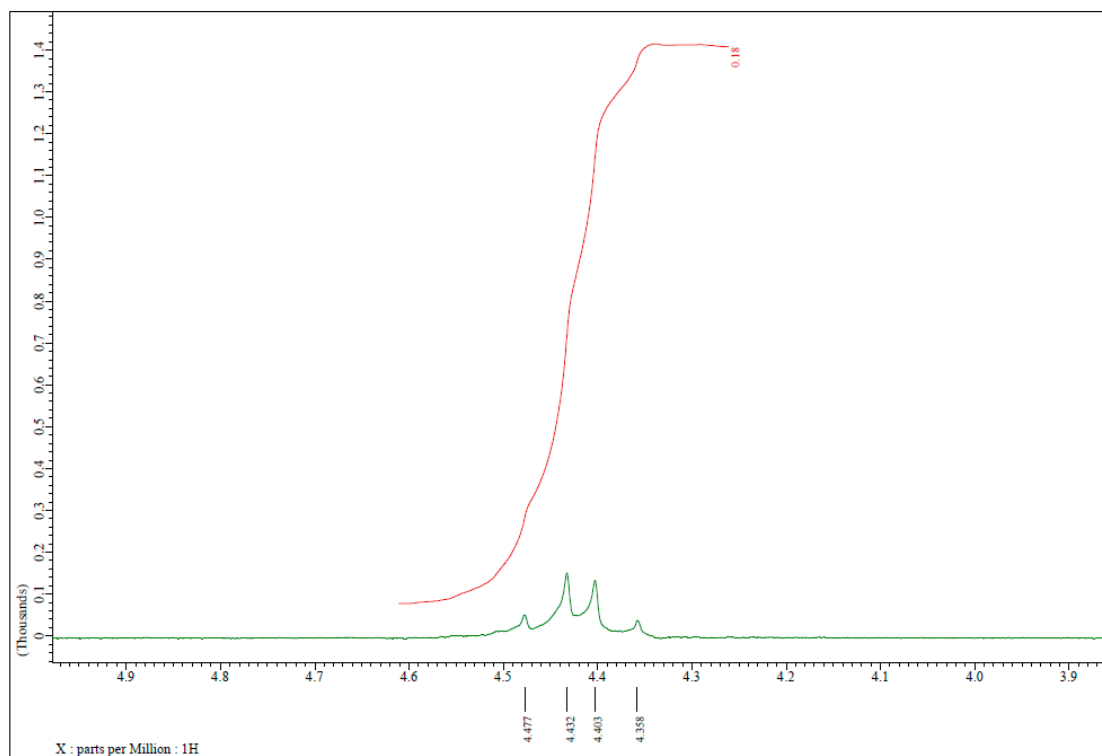

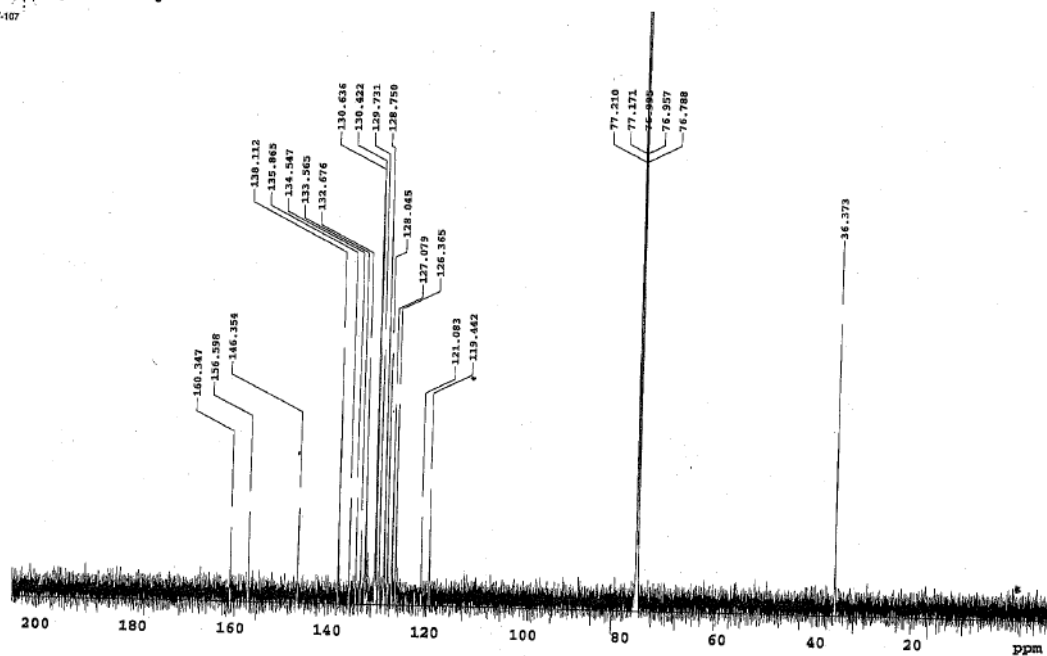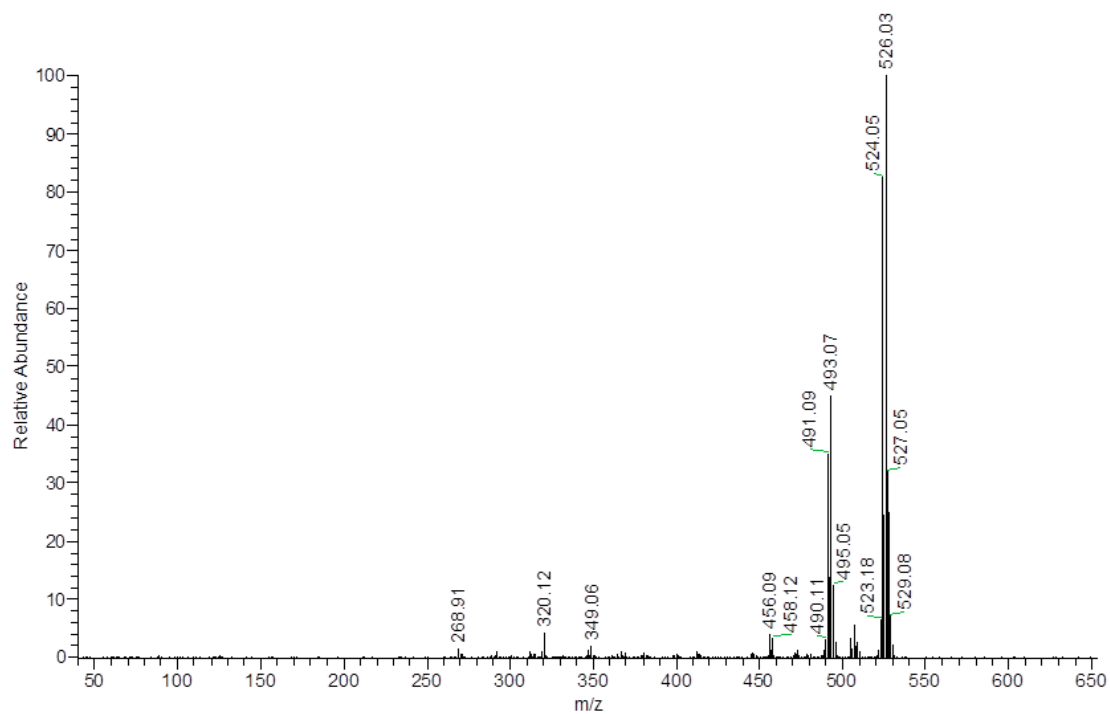

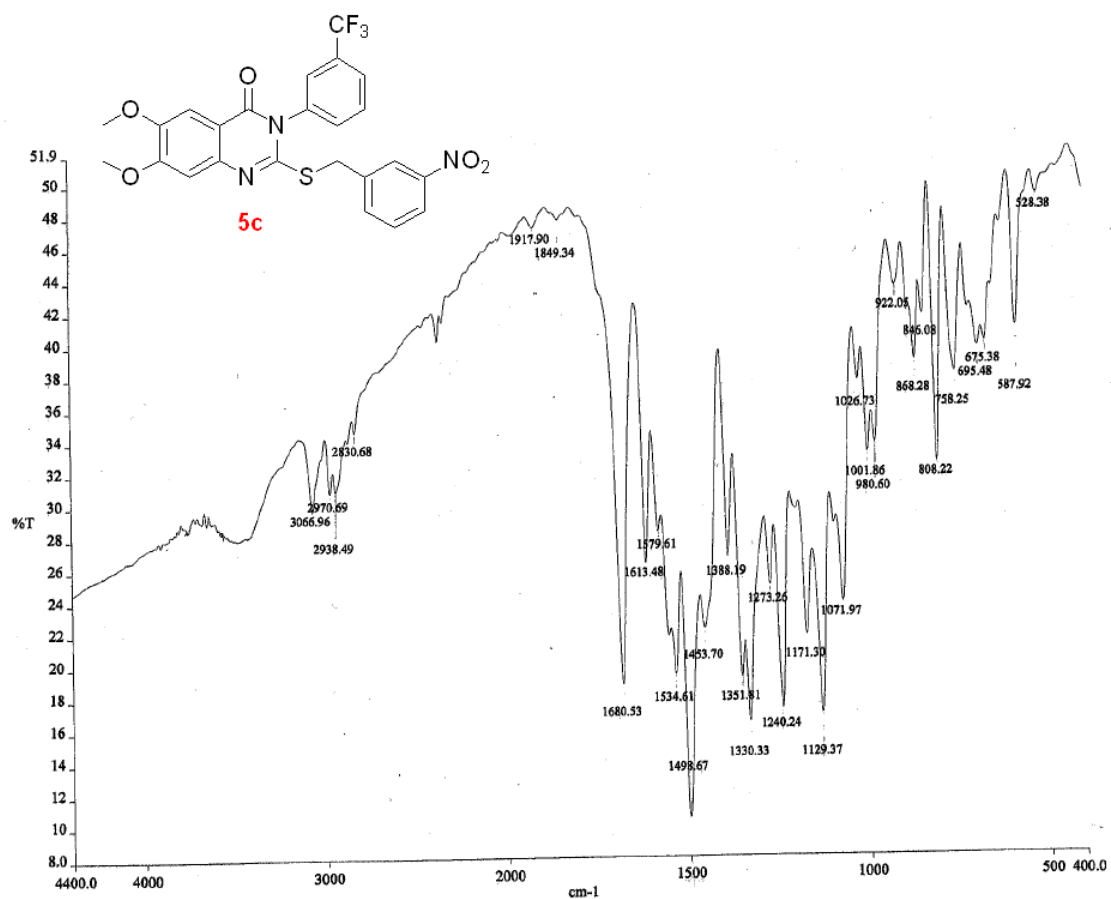

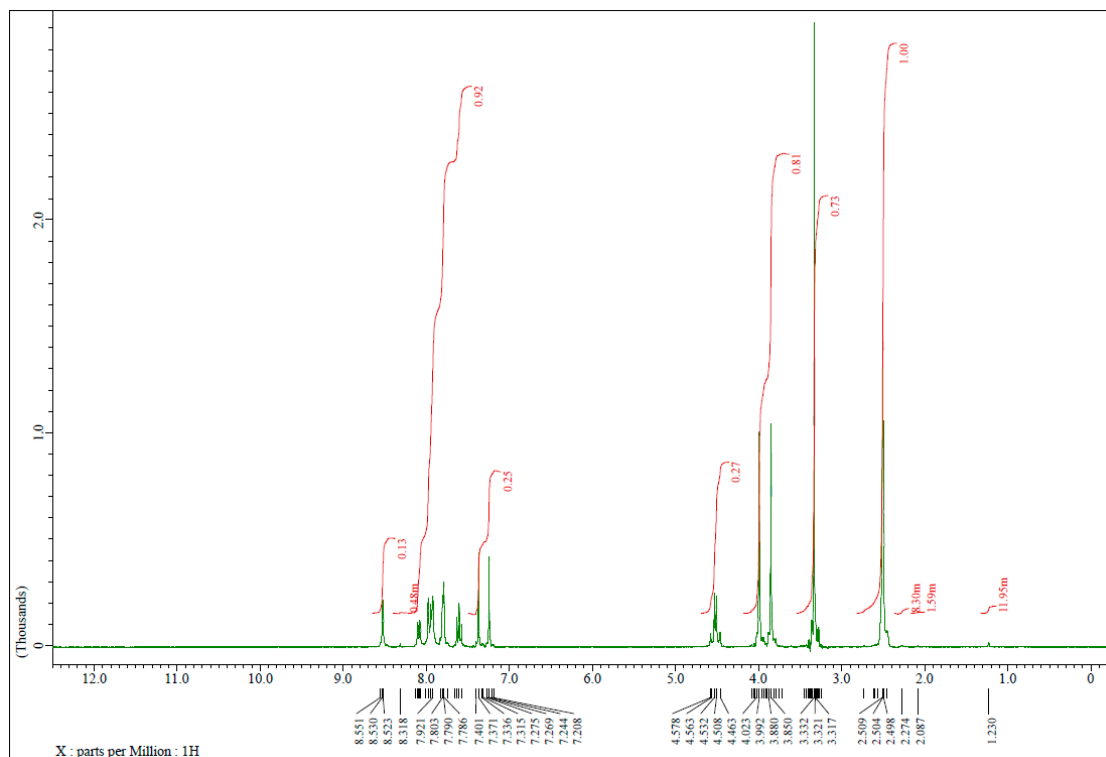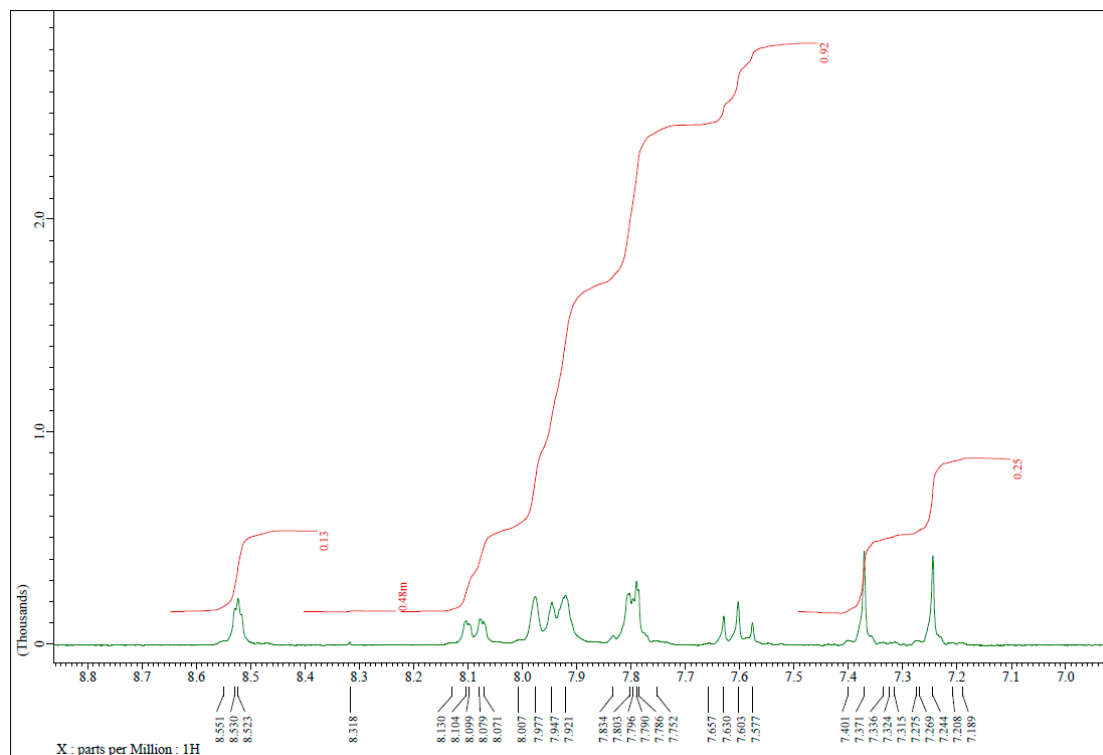

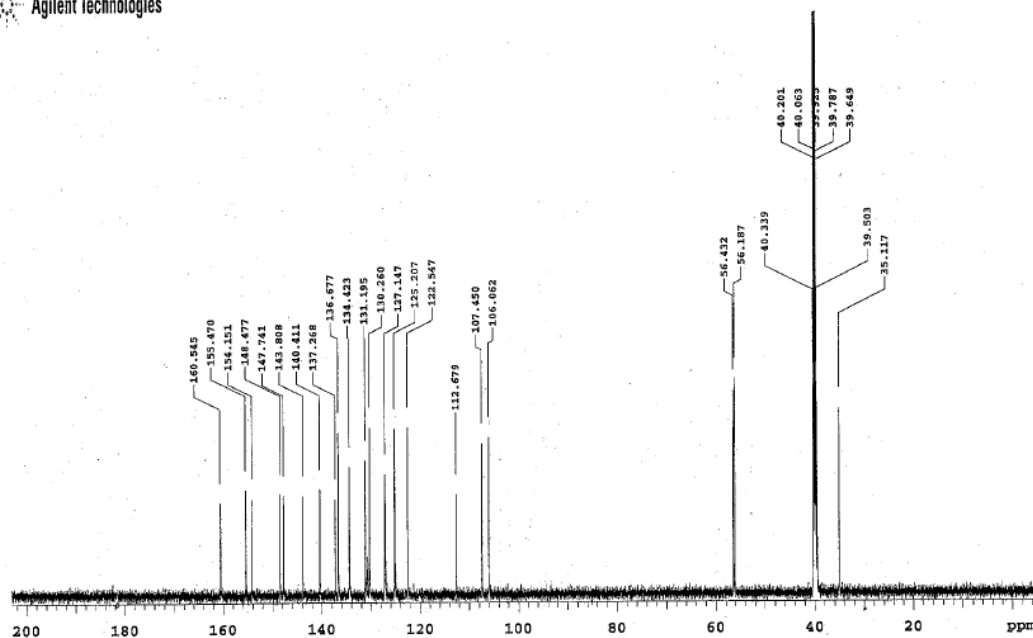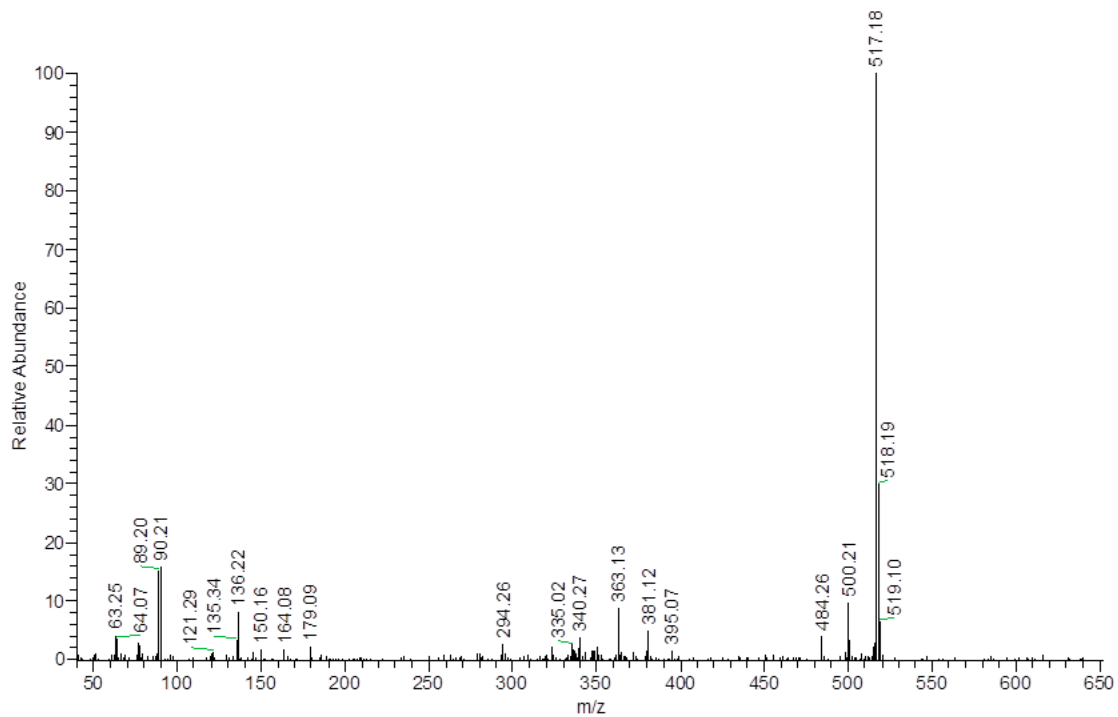

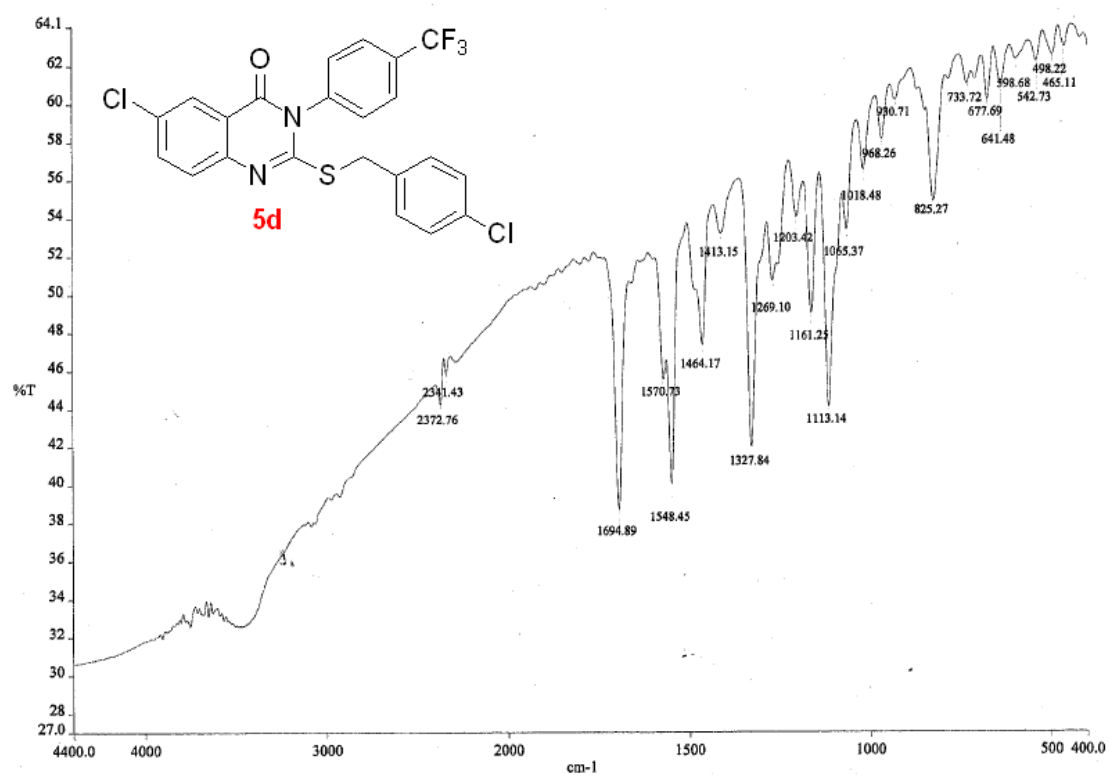

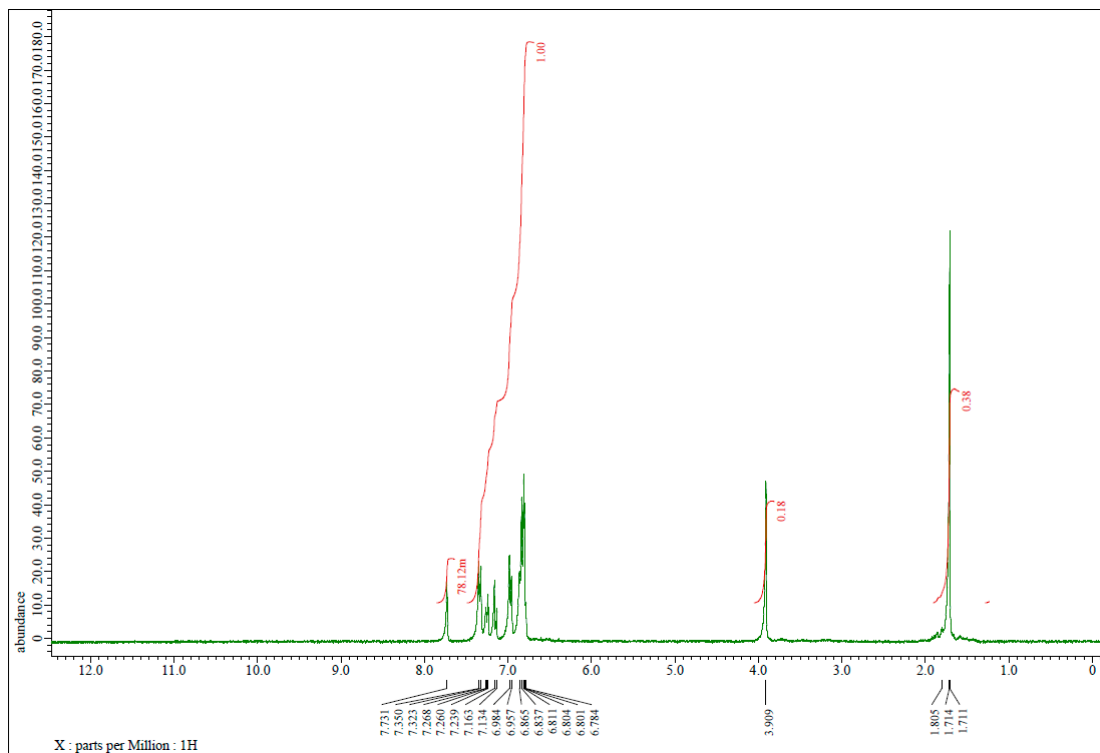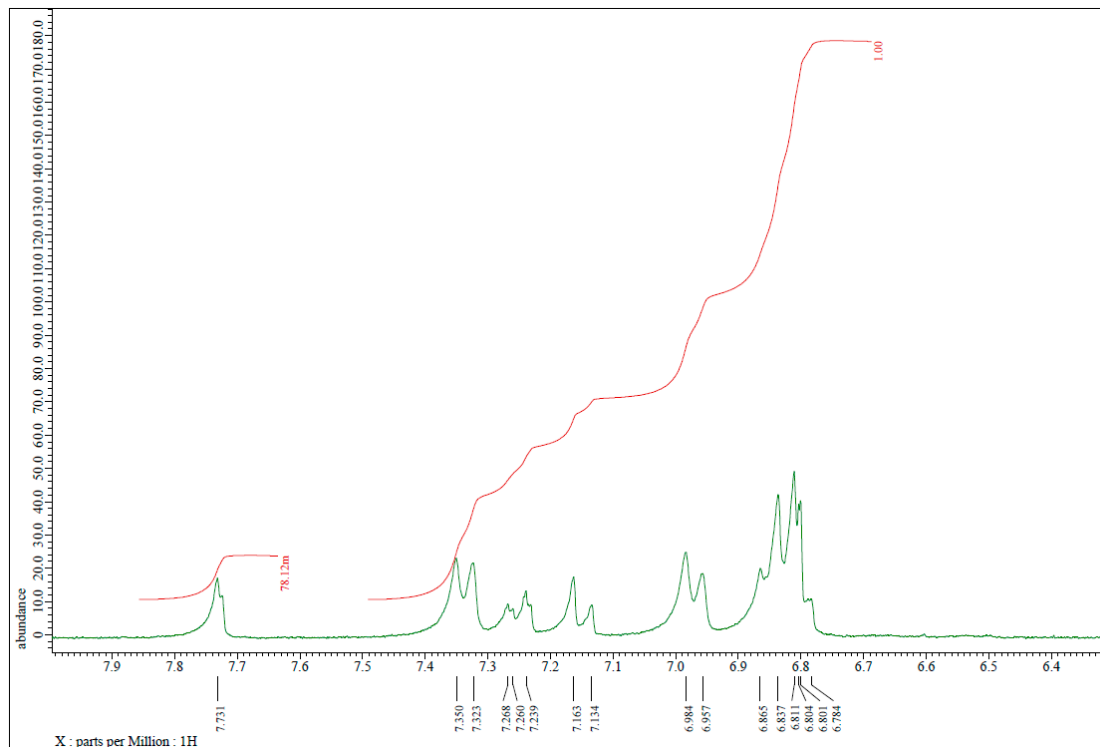

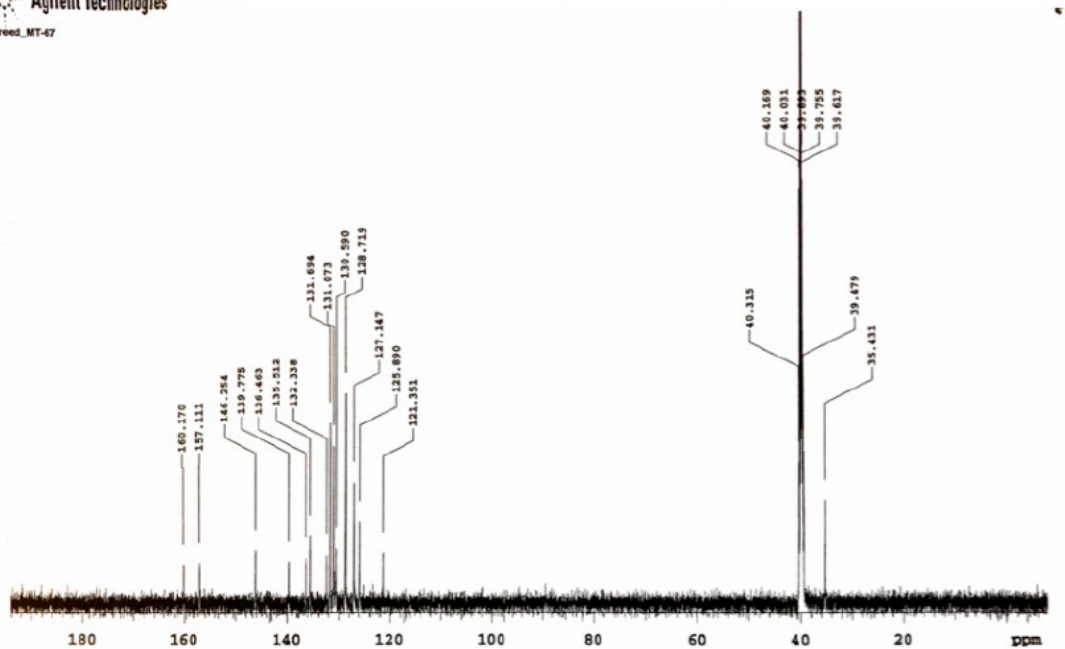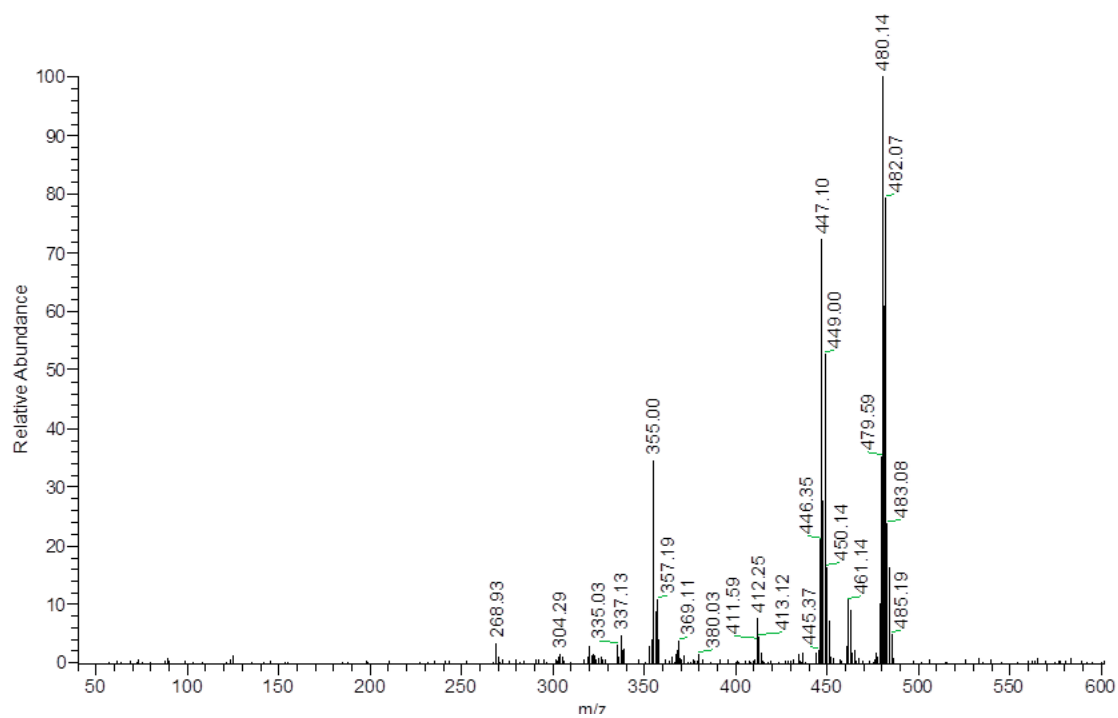



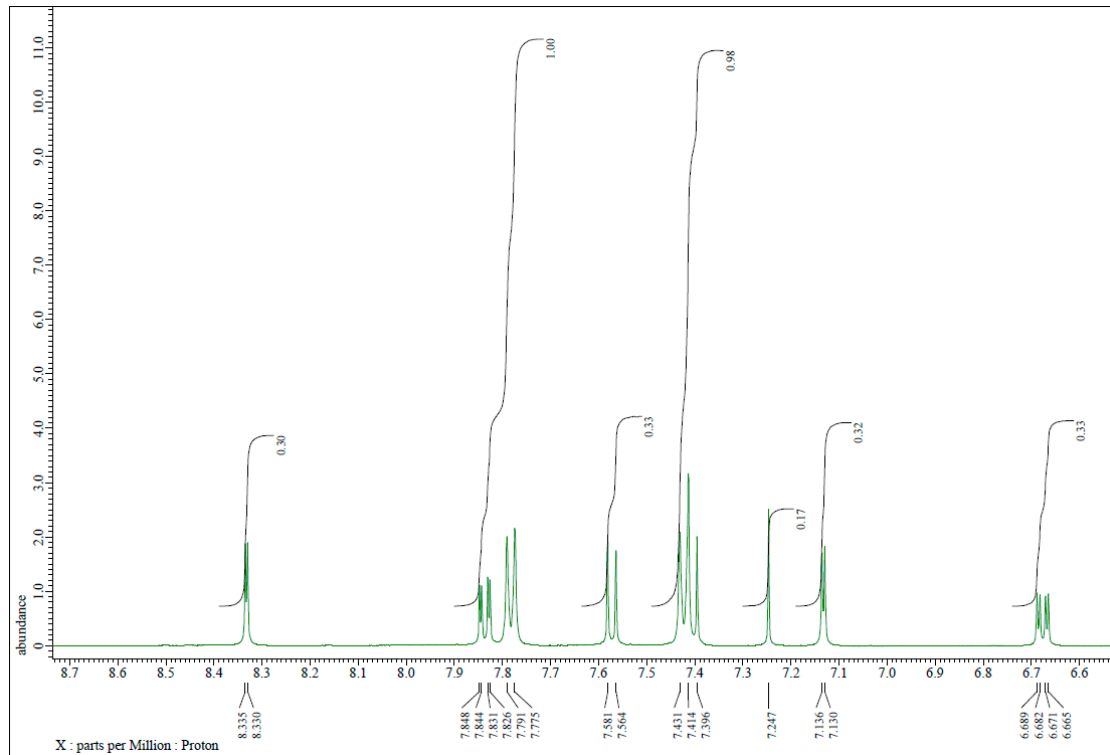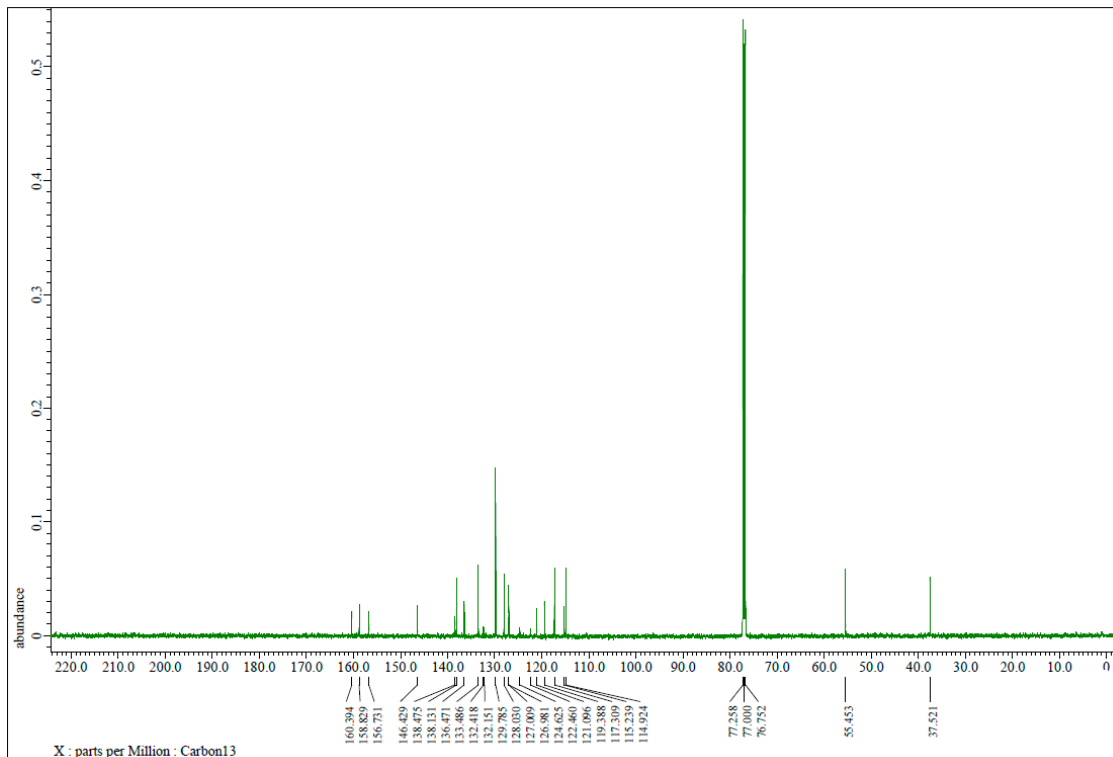

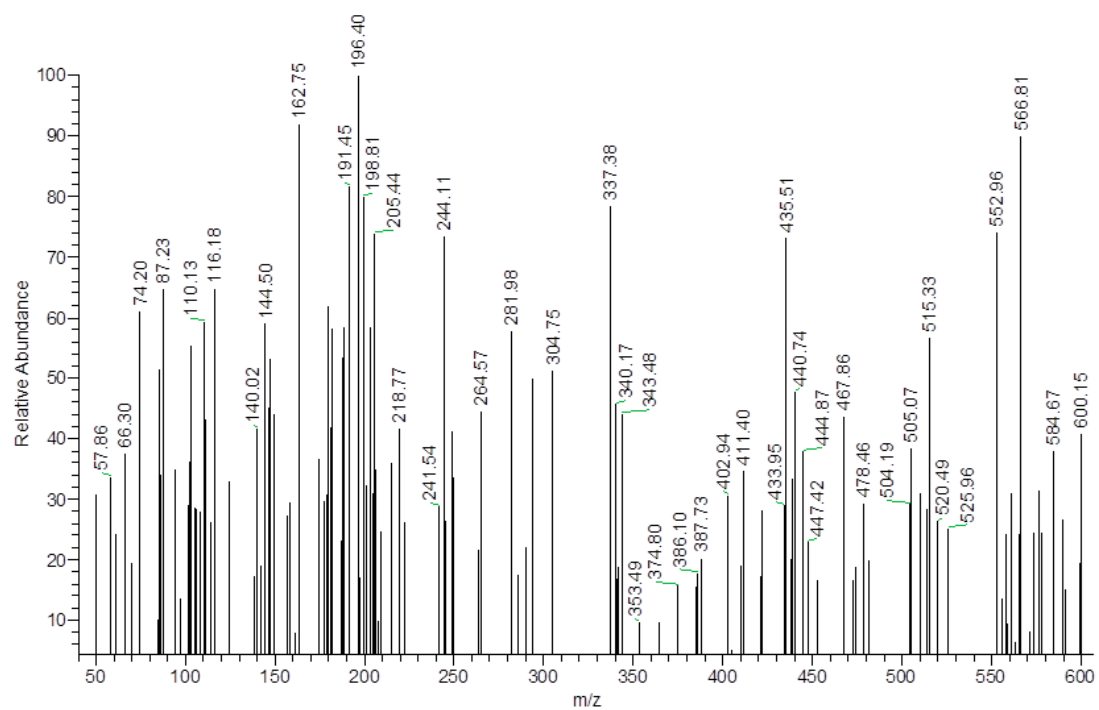

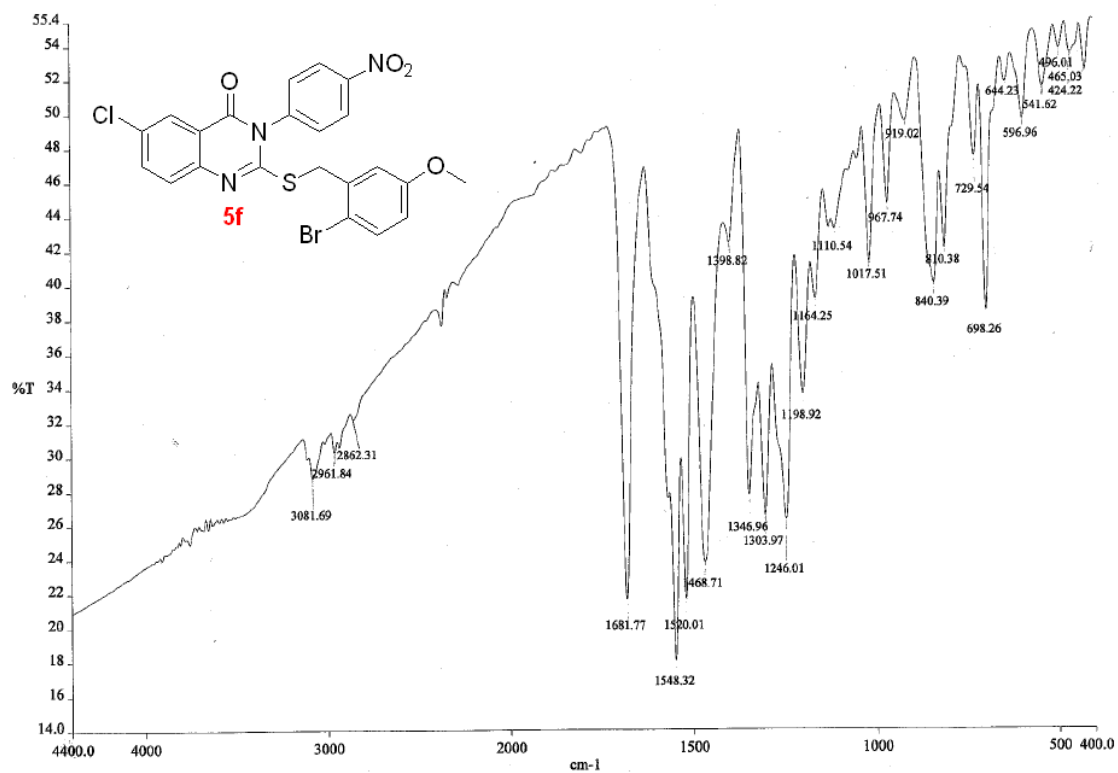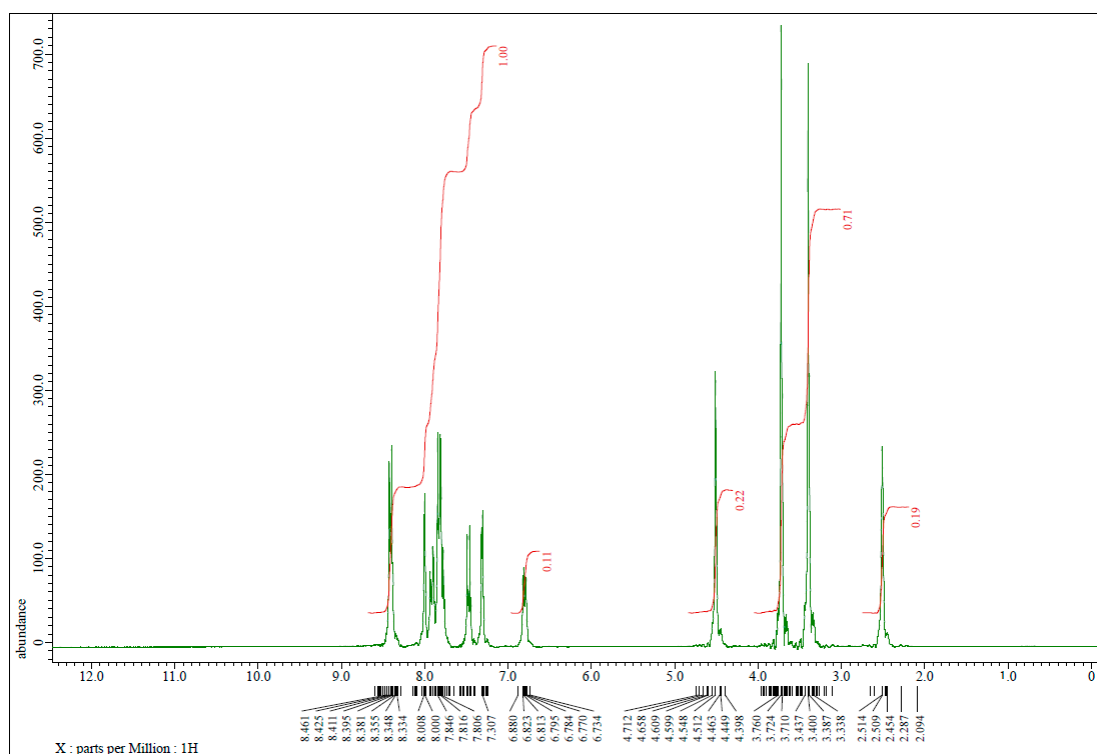

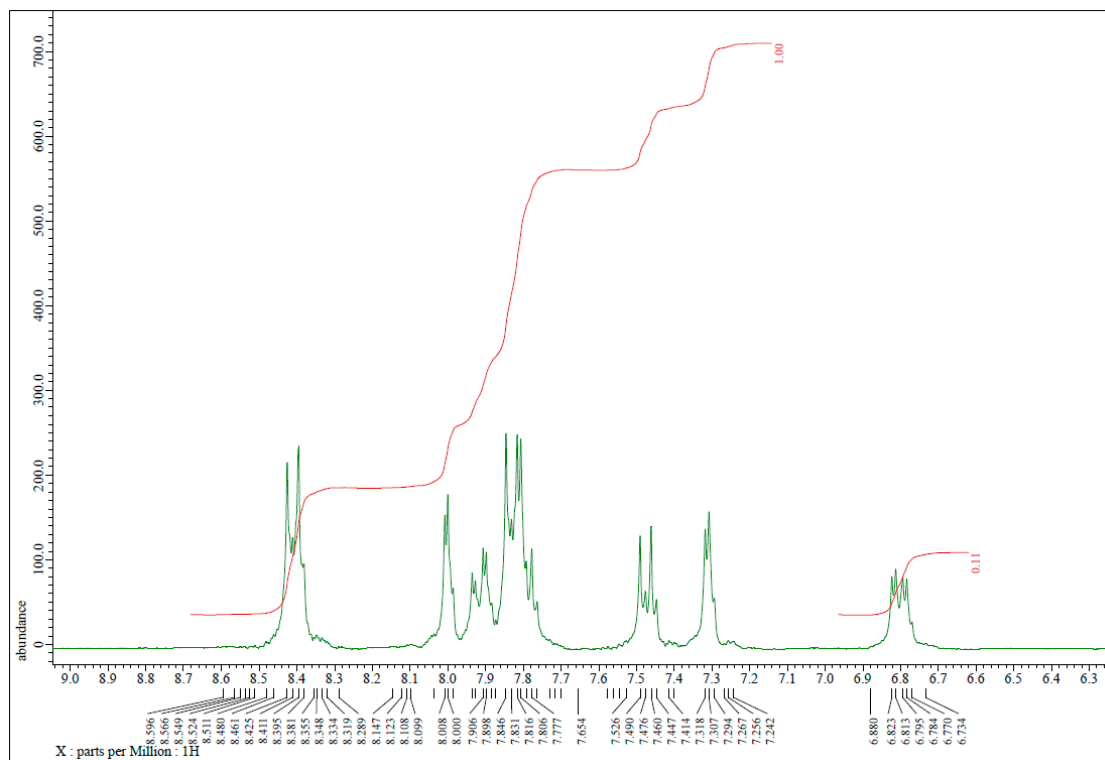

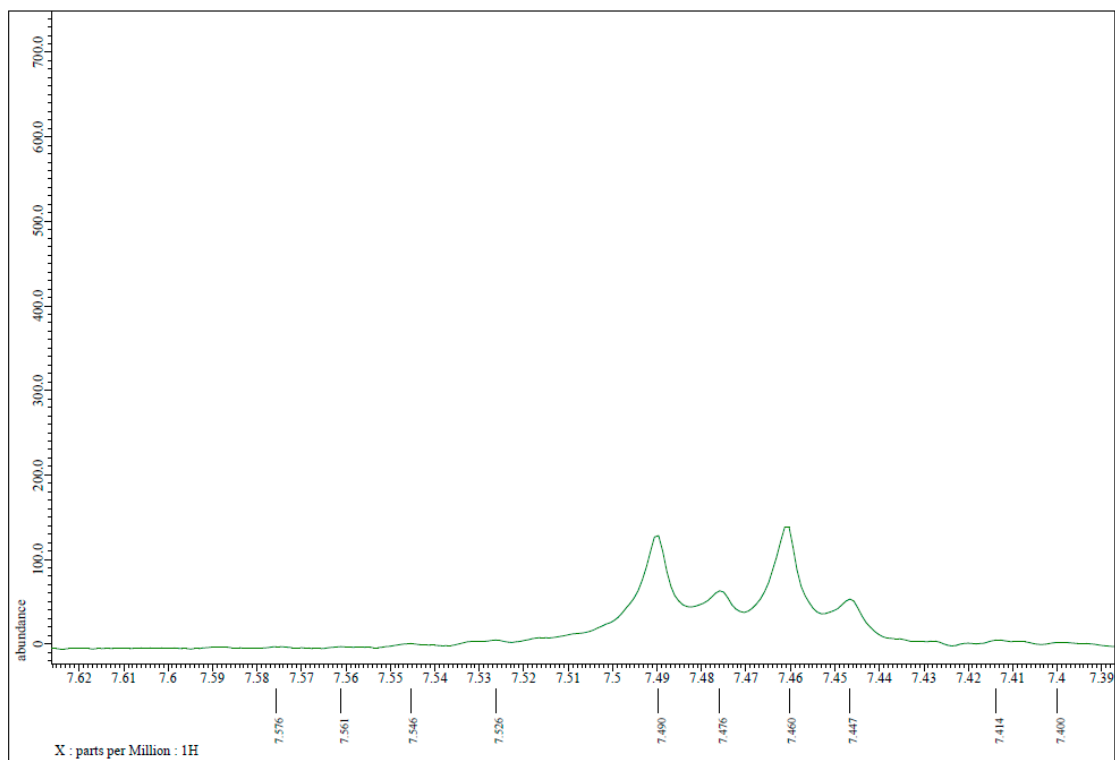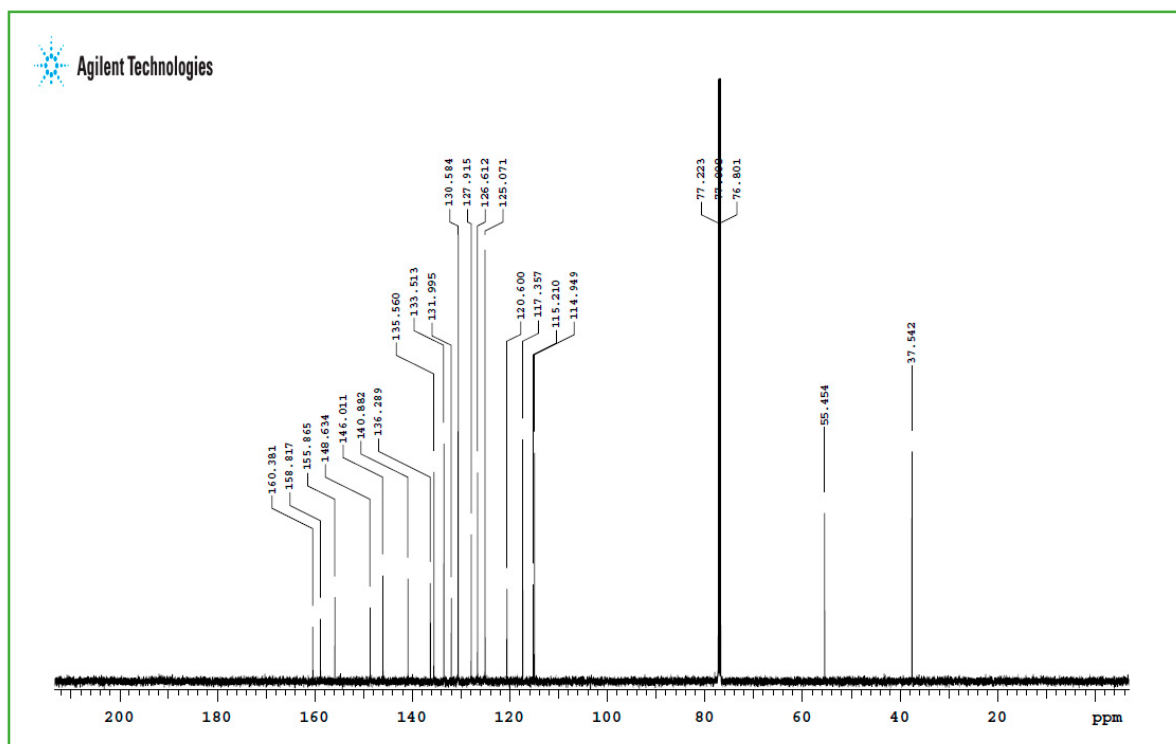

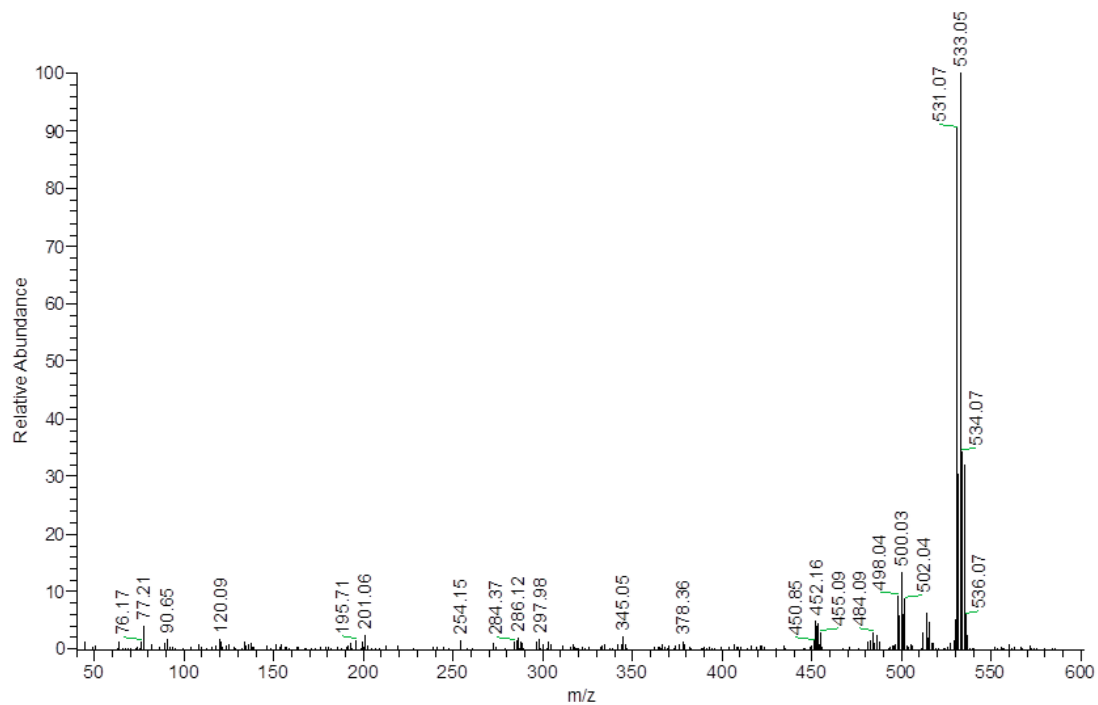

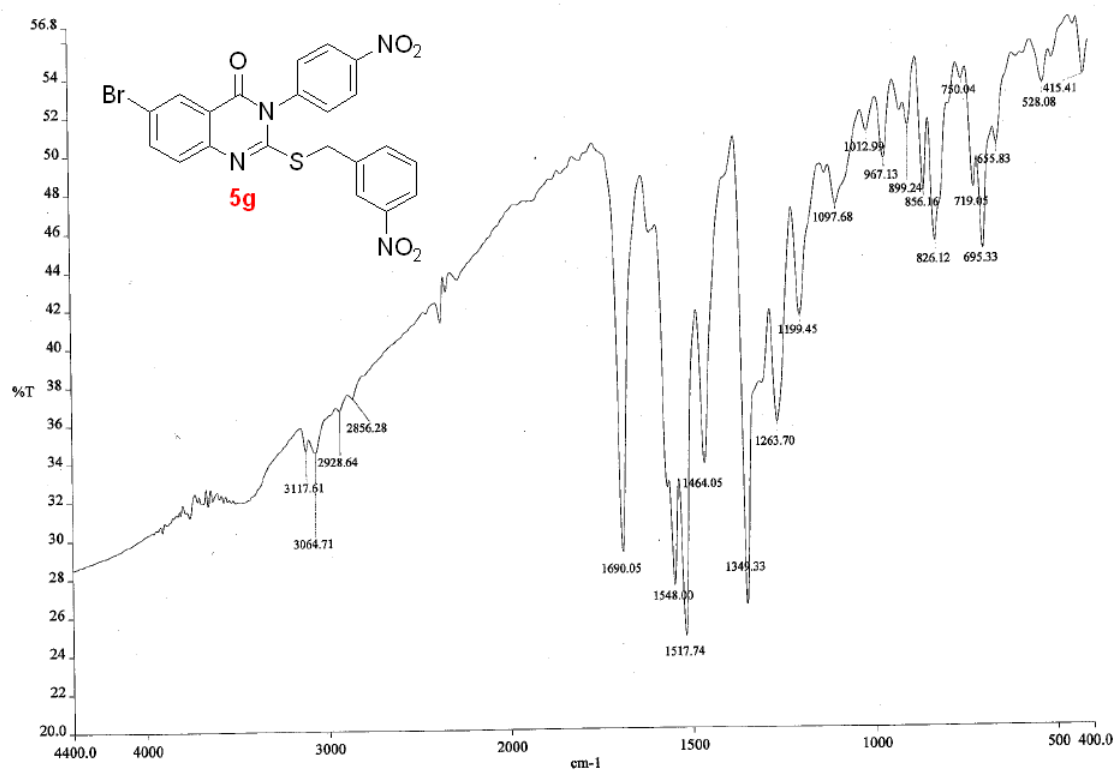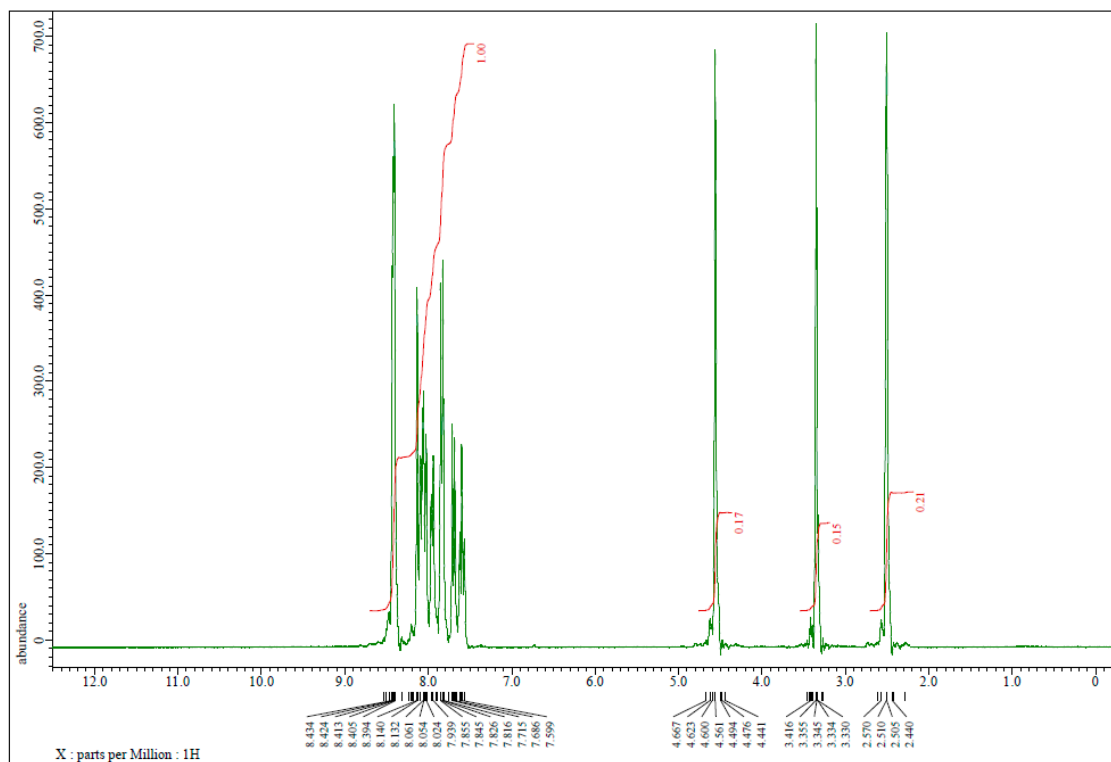

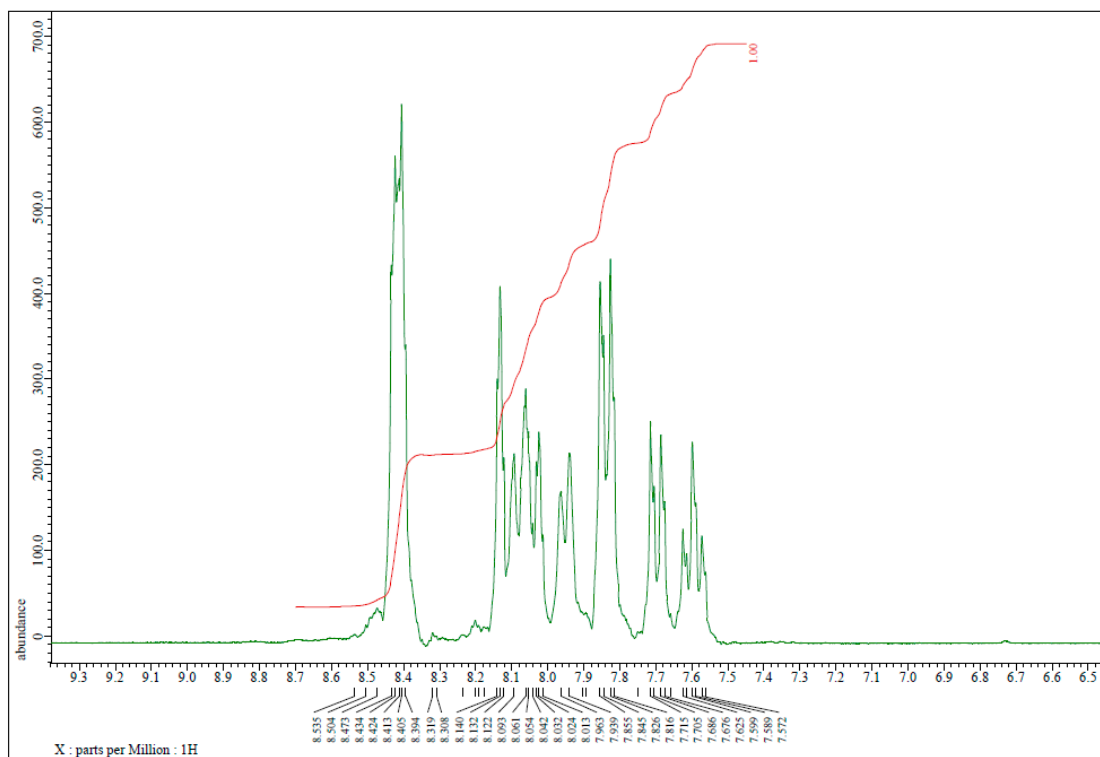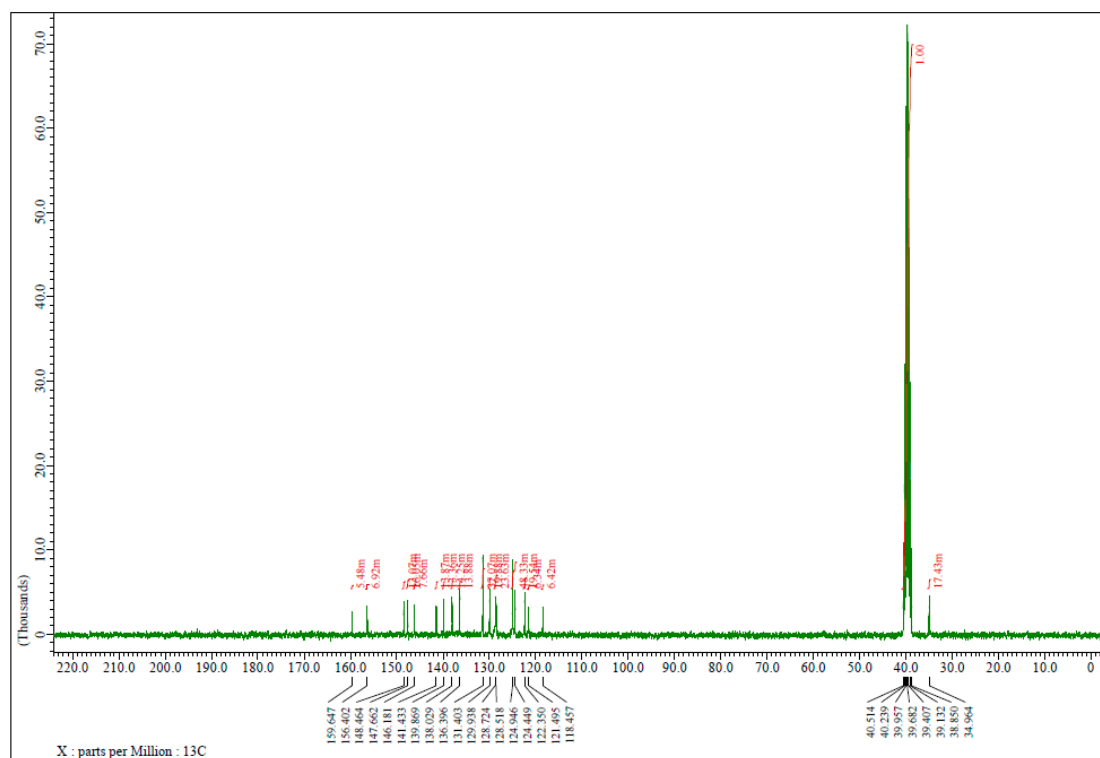

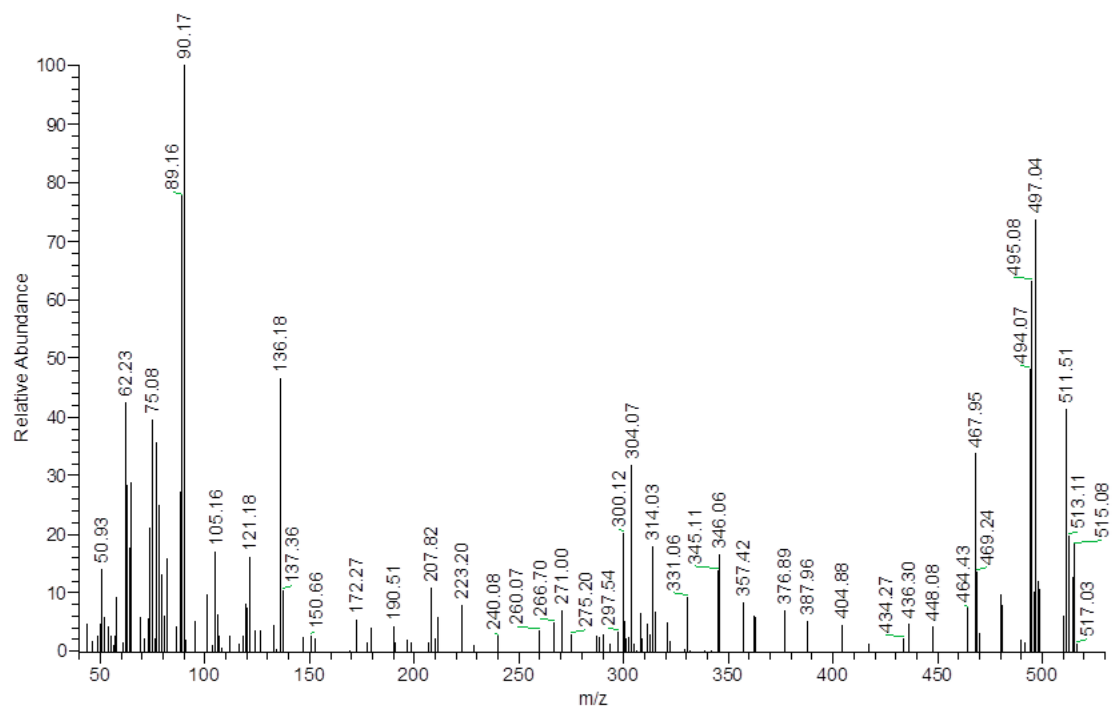

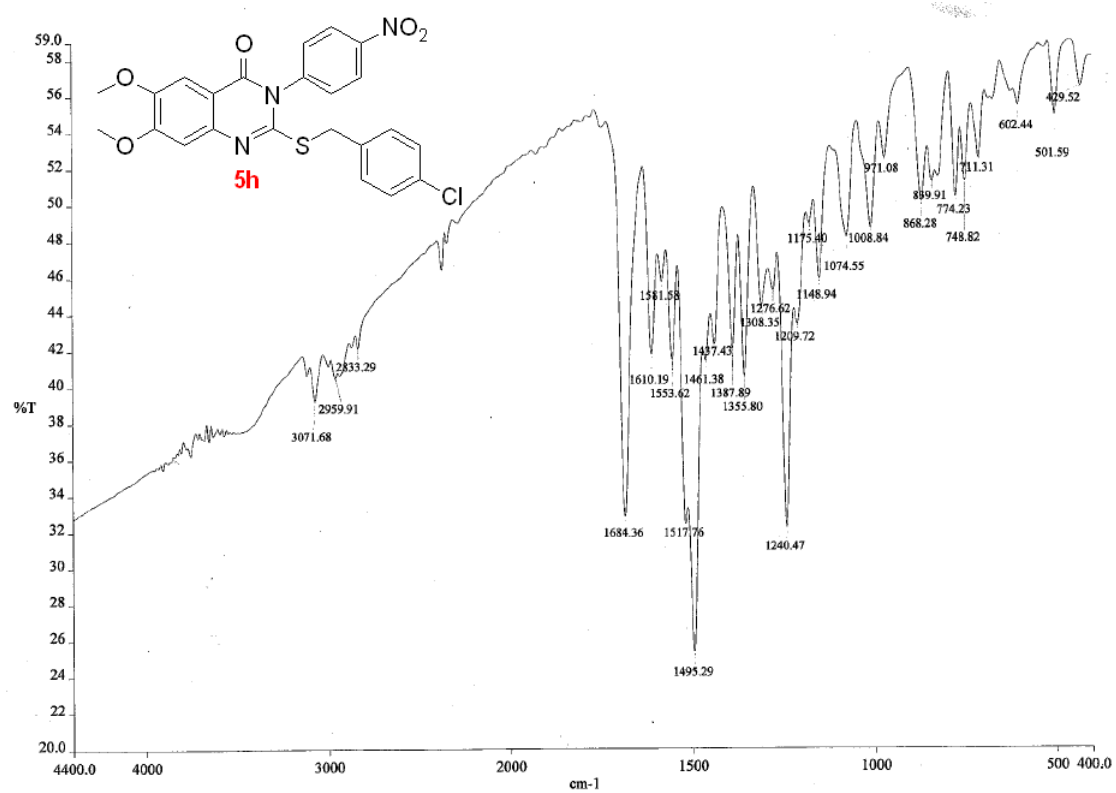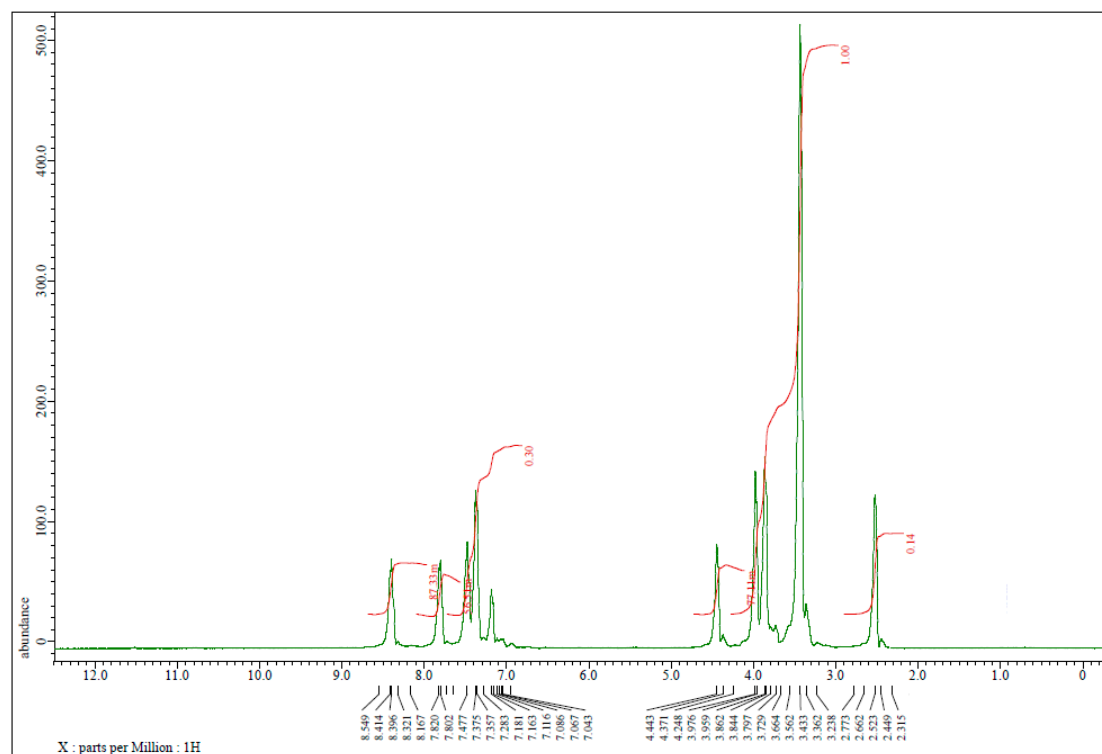

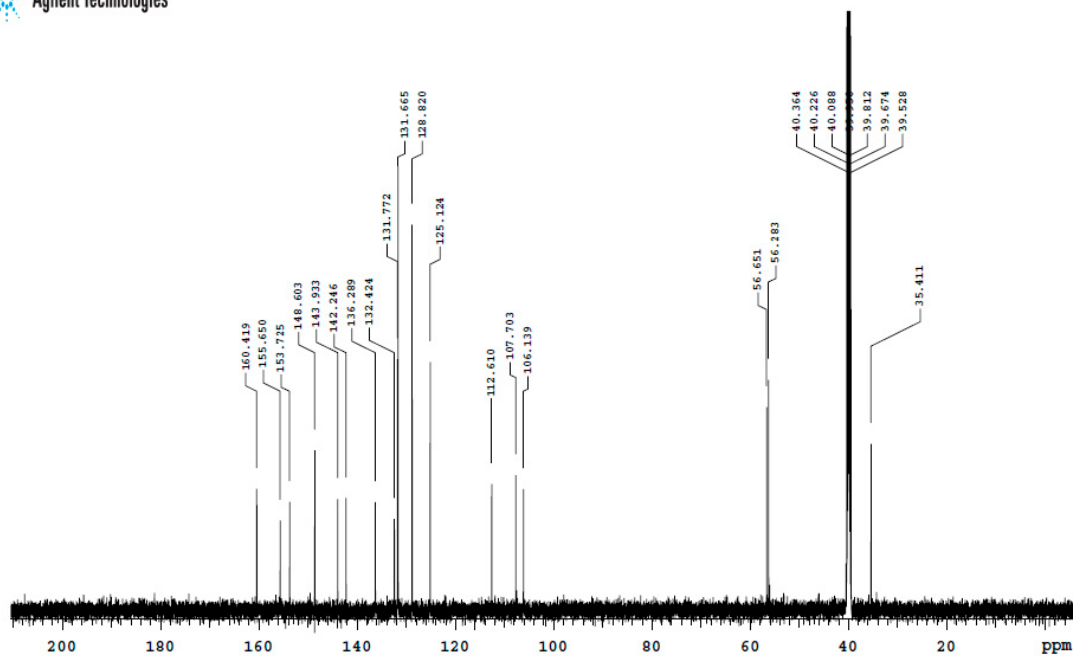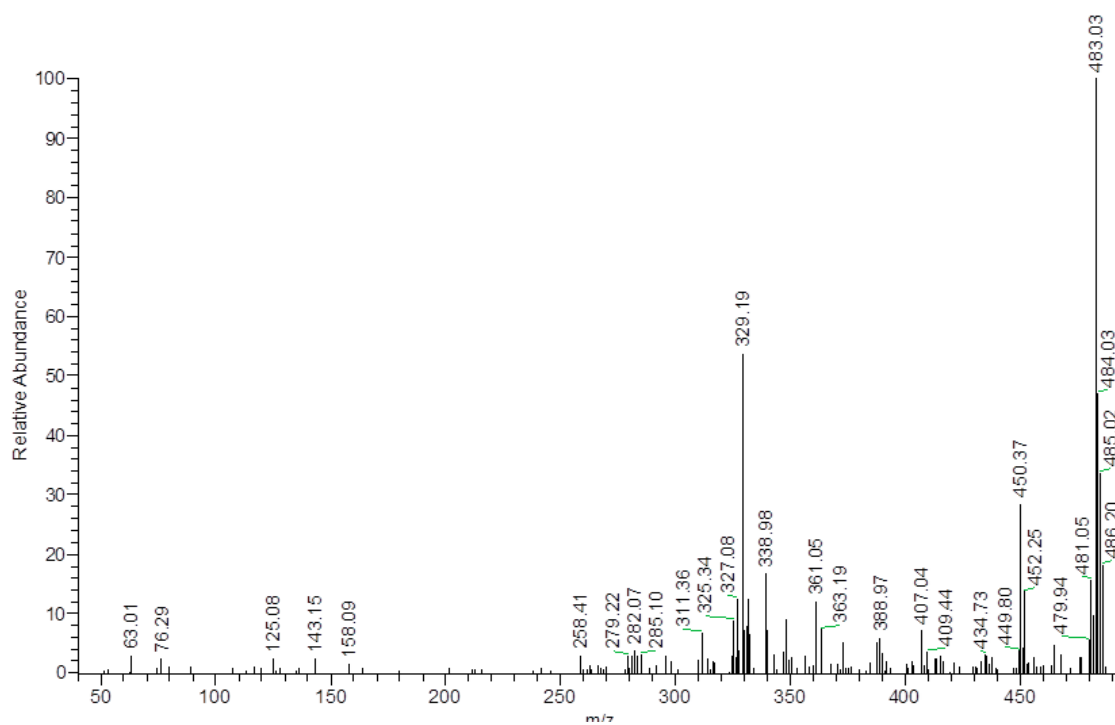

Supplement: Supplementary file 1 [file pharmaceuticals-16-01392-s001.zip › pharmaceuticals-2535181-supplementary/Supplementary Materials/2-Spectroscopic analyses.pdf]
